# Supplementary material for: Inferring high-resolution human mixing patterns for disease modeling
Source: Nat Commun. 2021 Jan 12;12:323. doi: 10.1038/s41467-020-20544-y (PMC7803761; doi:10.1038/s41467-020-20544-y)
Supplement: Supplementary file 1 — Supplementary Information [file 41467_2020_20544_MOESM1_ESM.pdf]

Inferring high-resolution human mixing patterns for disease modeling

Supplementary Information

Dina Mistry[1], Maria Litvinova[2,3], Ana Pastore y Piontti[2], Matteo Chinazzi[2], Laura Fumanelli[4], Marcelo F. C. Gomes[5], Syed A. Haque[2], Quan-Hui Liu[6], Kunpeng Mu[2], Xinyue Xiong[2], M. Elizabeth Halloran[7,8], Ira M. Longini Jr.[9], Stefano Merler[4], Marco Ajelli[10,2], Alessandro Vespignani[2,3]

[1] Institute for Disease Modeling, Bellevue, WA, USA
[2] Laboratory for the Modeling of Biological and Socio-technical Systems, Northeastern University, Boston, MA, USA
[3] ISI Foundation, Turin, Italy
[4] Bruno Kessler Foundation, Trento, Italy
[5] Fundação Oswaldo Cruz, Rio de Janeiro, Brazil
[6] College of Computer Science, Sichuan University, Chengdu, Sichuan, China
[7] Vaccine and Infectious Disease Division, Fred Hutchinson Cancer Research Center, Seattle, WA, USA
[8] Department of Biostatistics, University of Washington, Seattle, WA, USA
[9] Department of Biostatistics, College of Public Health and Health Professions, University of Florida, Gainesville, FL, USA
[10] Department of Epidemiology and Biostatistics, Indiana University School of Public Health, Bloomington, IN, USA

Contents

1 Synthetic populations **3**
1.1 Data sources 3
1.2 Adaptive algorithms for the development of synthetic households 3
1.3 Adaptive algorithms for the development of synthetic schools and workplaces . . 15

2 Contact Matrices **18**
2.1 Contact matrices by setting 18
2.2 Calibration of the overall contact matrix 27
2.3 The overall contact matrix 29
2.4 Assortativity of overall contact matrices 31
2.5 Uncertainty of overall contact matrices 33

3 Modeling the spread of the 2009 H1N1 pandemic influenza **40**
3.1 Calculation of the reproduction number 40
3.2 Calibration of the infection transmission model 40
3.3 Alternative method for the calculation of the reproduction number 45
3.4 Simulations of epidemics resembling the 2009 H1N1 pandemic influenza 46

4 Appendix **58**

1 Synthetic populations

1.1 Data sources

We employ publicly available macro and micro data from national census websites and surveys conducted on household, health and economic conditions in different sample populations around the world. Table 1 lists the sources of the wide array of publicly available macro and micro data we used to construct the synthetic populations in this study.

Country	Country Code	Sources
Australia	AUS	Australian Bureau of Statistics [1]
Canada	CAN	Statistics Canada [2]
		BC Stats [3]
		Finding Quality Childcare: A guide for parents in Canada [4]
China	CHN	China Health and Nutrition Survey [5]
		China Census 2010[6]
		China Statistical Yearbook [7]
India	IND	The 15th Indian Census [8]
		Demographic and Health Surveys (2005) [9]
		Unified District Information System for Education [10, 11]
		All India Survey on Higher Education [12]
Israel	ISR	Israel Census 2008 [13]
Japan	JPN	Official Statistics of Japan[14]
Russia	RUS	Russia Longitudinal Monitoring Survey [15]
		2010 All-Russian Population Census [16]
		Federal State Statistics Service [17]
South Africa	ZAF	Statistics South Africa [18]
		Statistics on Post-School Education and Training in South Africa [19]
		World Health Survey (2003) [20]
		South African Revenue Service [21]
United States of America	USA	Decennial Census of Population and Housing [22]
		Current Population Survey [23]
		American Community Survey [24]
		IPUMS USA [25]

Supplementary Table 1: Data sources for each country and the country code.

1.2 Adaptive algorithms for the development of synthetic households

As an illustration of the procedure described in Materials and Methods, let us take a deeper look at the algorithm used to generate synthetic population of Maharashtra, a large state centrally located in India and home to the city of Mumbai. In order to reconstruct Maharashtra we start with generating the households where the synthetic population resides (see Scheme S1 for a sketch of this algorithm).

The census website hosted by the government of India provides multiple macro level datasets used in this algorithm (see Table 1), the first of which is the count of the number of households by size (or size bracket) in each state or territory. From this count, we generate a multinomial distribution of the first kind for the household sizes $\mathcal{M}(h_s)$ and sample the size h_s^i for the ith household. Next, we work with micro level survey data on households in Maharashtra from the World Health Survey (WHS) to generate a multinomial distribution of the second kind for the age of the head of the household by the household size $\mathcal{M}(a_{hh}|h_s^i)$, and sample the age of the head of the household a_{hh}^i. With both the size and age of the household head determined, we then use the survey data again to create the probability distribution $\mathcal{M}(h_f|a_{hh}^i, h_s^i)$ to sample the household composition h_f^i.

The household composition h_f describes the type of household and generally gives an indication of the relationships between household members. In the case of India, the household

Algorithm 1 Synthetic Households

procedure (build N_H households)
  for $i \leftarrow 1$ **to** N_H **do**
    $h_s^i \leftarrow$ Sample $\mathcal{M}(h_s)$ for household size
    $a_{hh}^i \leftarrow$ Sample $\mathcal{M}(a_{hh}|h_s^i)$ for age of household head conditional on h_s^i
    add a_{hh}^i to household h^i
    $h_f^i \leftarrow$ Sample $\mathcal{M}(h_f|a_{hh}^i, h_s^i)$ for household composition conditional on a_{hh}^i, h_s^i
    if h_f^i includes a spouse for a_{hh}^i **then**
      $a_{sp}^i \leftarrow$ Sample $\mathcal{M}(a_{sp}|a_{hh}^i)$ for spouse age conditional on a_{hh}^i
      add a_{sp} to household h^i
    for r in *relations** **do**
      $a_r^i \leftarrow$ Sample $\mathcal{M}(a_r|a_{hh}^i)$ for age of relation r
      add a_r^i to household h^i
    for r in *relations*† **do**
      $a_r^i \leftarrow$ Sample $\mathcal{M}(a_r|a_{hh}^i)$ for age of relation r
      add a_r^i to household h^i
    while size(household) $< h_s^i$ **do**
      for r in *relations*† **do**
        $n_r^i \leftarrow$ Sample $\mathcal{M}(n_r|a_{hh}^i)$ for the number of relation r in $h_f^i > 1$
        for $j \leftarrow 1$ **to** n_r^i **do**
          $a_r^j \leftarrow$ Sample $\mathcal{M}(a_r|a_{hh}^i)$ for age of relation r
          add a_r^j to household h^i

* Refers to household members with relationship r in reference to the head of the household limited to a maximum count of 1. This may include a mother, father, mother-in-law, or father-in-law. The specific set of relations is indicated by the household composition h_f^i.

†Refers to household members with relationship r in reference to the head of the household. This may include a son, daughter, son-in-law, daughter-in-law, brother, sister, brother-in-law, sister-in-law, aunt, uncle, niece, nephew, or grandchild. In the case of married individuals, it is often best to sample their ages together, where the age of one is chosen conditional on the other as a spouse. The specific set of relations is indicated by the household composition h_f^i.

Scheme S1: Algorithm 1 to build synthetic households

composition listed in the survey data was inadequate to describe the actual relations within each household. As a result, we generated the household composition by aggregating for each household the type of relationship each household member has in reference to the head of the household. This set of relationships can be any mix of: husband or wife, son, daughter, son-in-law, daughter-in-law, brother, sister, brother-in-law, sister-in-law, mother, father, mother-in-law, father-in-law, niece, nephew, grandchild, and other relations. The set of these relations in a household, along with the head of the household constitutes a specific household composition h_f. With h_f^i sampled from $\mathcal{M}(h_f|a_{hh}^i, h_s^i)$ we can move on to filling out the household up to the household size with the specific members and their respective ages with respect to the already determined household characteristics. For example, if h_f^i includes a wife or husband of the head of the household, then we first determine the age of the household head's spouse using $\mathcal{M}(a_{sp}|a_{hh}^i)$, a multinomial distribution generated from the micro survey describing the age of the spouse of the head of the household by the age of the head of the household.

Next we determine the age of a mother, father, mother-in-law, and/or father-in-law of the head of the household for each type of said relation in the household composition. We sample the age of each parent a_p using the survey data to form the multinomial distribution $\mathcal{M}(a_p|a_{hh}^i)$ conditional on the age of the head of the household, and then resample a_p from the census age structure as mentioned above.

For the other types of household members, we first need to determine the number of each relation in the household based on the already defined household characteristics. For example, in the case of children being in the household composition, we calculate from the survey data the multinomial distribution $\mathcal{M}(n_c|a_{hh}^i)$ of the number of children in a household conditional

Age Structure: Census vs. Synthetic Pop. Statistical Tests				
Location	Country	Pearson's r	KS	RMSE
New South Wales	AUS	1.00	0.08	0.00
Northern Territory	AUS	1.00	0.06	0.00
Queensland	AUS	1.00	0.06	0.00
Alberta	CAN	1.00	0.06	0.00
Ontario	CAN	1.00	0.07	0.00
Quebec	CAN	0.99	0.07	0.00
Beijing	CHN	1.00	0.11	0.12
Guizhou	CHN	0.98	0.11	0.41
Sichuan	CHN	1.00	0.11	0.06
Meghalaya	IND	0.99	0.06	0.01
Maharashtra	IND	0.98	0.06	0.01
Rajasthan	IND	0.96	0.06	0.05
Israel	ISR	0.99	0.08	0.00
Tokyo	JPN	1.00	0.07	0.00
Okinawa	JPN	1.00	0.07	0.00
Yamagata	JPN	1.00	0.06	0.00
Moscow	RUS	0.99	0.11	0.00
Chukotka	RUS	0.99	0.11	0.01
St. Petersburg	RUS	0.99	0.08	0.00
Free State	ZAF	1.00	0.06	0.00
Limpopo	ZAF	1.00	0.06	0.00
Western Cape	ZAF	1.00	0.06	0.00
New York	USA	1.00	0.11	0.01
Texas	USA	1.00	0.11	0.00
Utah	USA	1.00	0.17	0.01

Supplementary Table 2: Table of statistical tests comparing the census and synthetic population age distributions for a sample of locations. Country codes are used to refer to the country (refer to table 1 for the country codes).

on the age of the head of the household. We sample the number of children to be added to the household n_c^i and add up to $\min(n_c^i, h_s^i)$ children to ensure the household size is respected. The age of the children are then sampled from the multinomial distribution $\mathcal{M}(a_c|a_{hh}^i)$, again generated from the survey data conditional on the age of the head of the household. The number of other household members and their ages are sampled using a similar process to sample the children. Scheme S1 shows a sketch of this algorithm to build the synthetic population and the households in which they reside.

For each location, the algorithm laid out above needs to be modified to accommodate and take in the available data for the location, whether it be from a micro survey as in the case of locations in India, or from an online census database as in the case of locations in Australia [9, 1].

Using this initial procedure we found that several adjustments were in fact needed to reconstruct a synthetic population whose age structure truly reflects the age structure from the census. First, the sampling process to select the age of children was modified to sample conditional to either the age of the household head, or the age of the spouse of the household head (the multinomial distribution for this is yet again calculated from the survey data). We determine the probability of selecting the age of each child conditional on a_h^i or a_{sp}^i through a random walk until the final synthetic and census age structures pass a goodness of fit test at a level of generally 5-10% significance.

Similarly, the sampling procedure to determine the age of married household members (other than the head and their spouse) is modified to incorporate information we have on the age gaps

between married couples. In particular, in the instance of a married son or daughter and son- or daughter-in-law, we first sample the age of a married son or daughter a^i_{mc} conditional on the age of the household head. Then we sample the age of the married son-or or daughter-in-law conditional on a^i_{mc} using $\mathcal{M}(a_{sp}|a_h = a^i_{mc})$. We make use of the multinomial distribution for the spousal age conditional on the age of the household head here since the number of households with married children tends to be insufficient to generate an unbiased probability distribution, and we expect for the age gaps between spouses to remain consistent based on the ages of the couple, and not on their relation to the household head. A similar procedure is also used to determine the ages of married siblings and their respective spouses.

In all of this, the most challenging, and yet crucial aspect is linking the household characteristics such as the size or the composition to the ages of the household members. A degree of uncertainty remains in determining the exact age of each household member, pronounced by the use of micro survey data on small sample sizes. To avoid favoring the age structure of the sample, and instead rely on the macro data coming from the census, we resample the age of each household member according to the census age structure in a two or three year interval of the initial selected age of said member. With the ages of the household members resampled for all households, the characteristics of the resulting synthetic households are compared with the distributions of summary statistics available from the macro level data using a goodness of fit test at the desired level of significance (generally 5%). For example we compare the age structure of all of the generated households together to the census age structure and report the results of a battery of statistical tests on the two distributions for a sample of subnational locations in Table 2 and Figures 1-2. In addition to this, Figures 3-4 show the correlation of census and synthetic age distributions for this set of subnational locations. In Table 2 we include the results of the Pearson correlation coefficient, the Kolmogorov-Smirnov test, and the root-mean-squared error (RMSE). This procedure of generating a synthetic population is iterated over until a satisfactory fit is reached, incorporating additional joint distributions or limiting factors (estimated from available data) in the procedure with each iteration. The results of these tests for all 277 subnational locations and Israel are reported in the Appendix (see Tables 6-7).

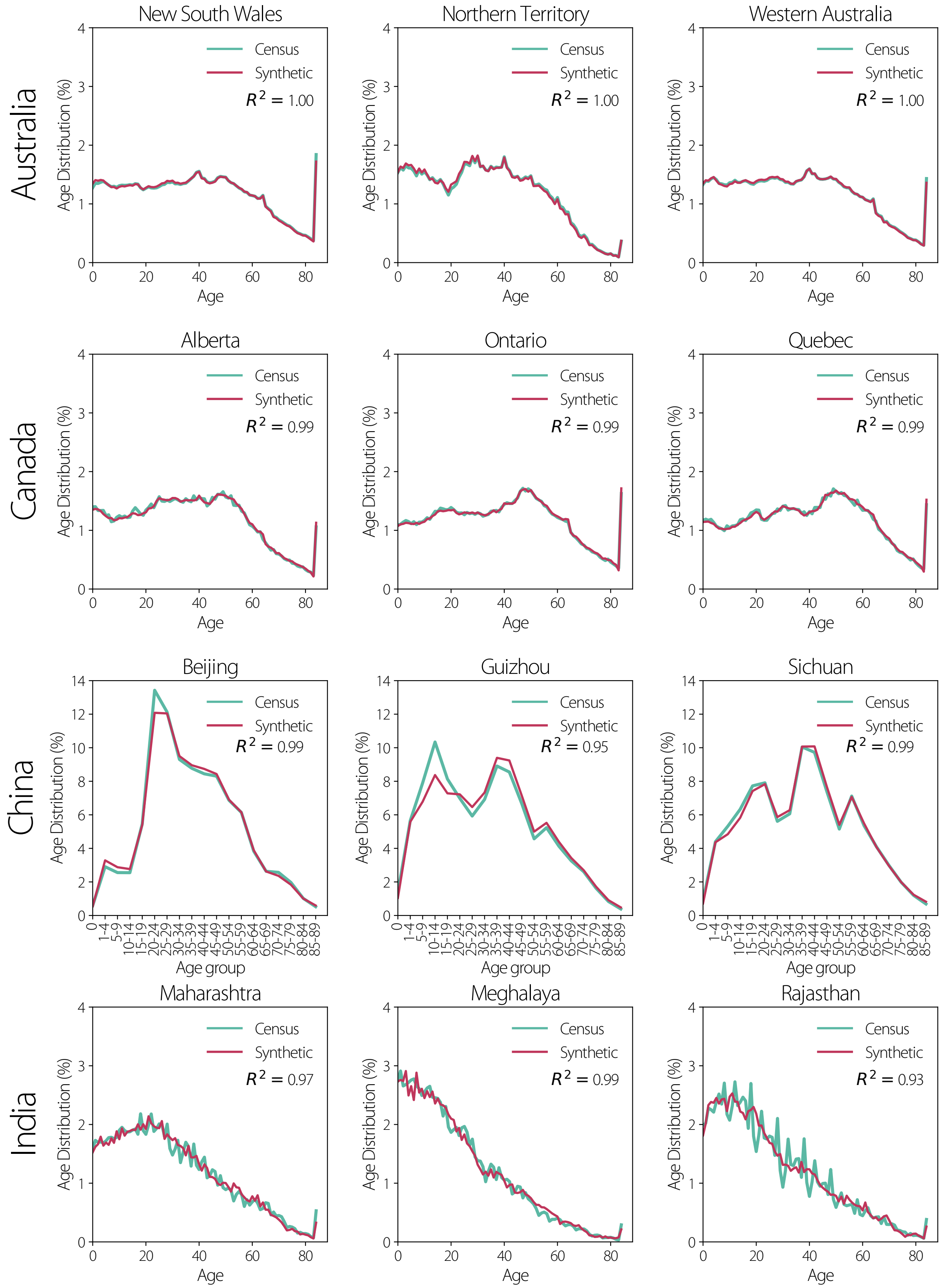

Supplementary Figure 1: **Age Distributions** Comparison of the census and synthetic age distributions for 3 subnational locations in Australia (first row), Canada (second row), China (third row), and India (last row).

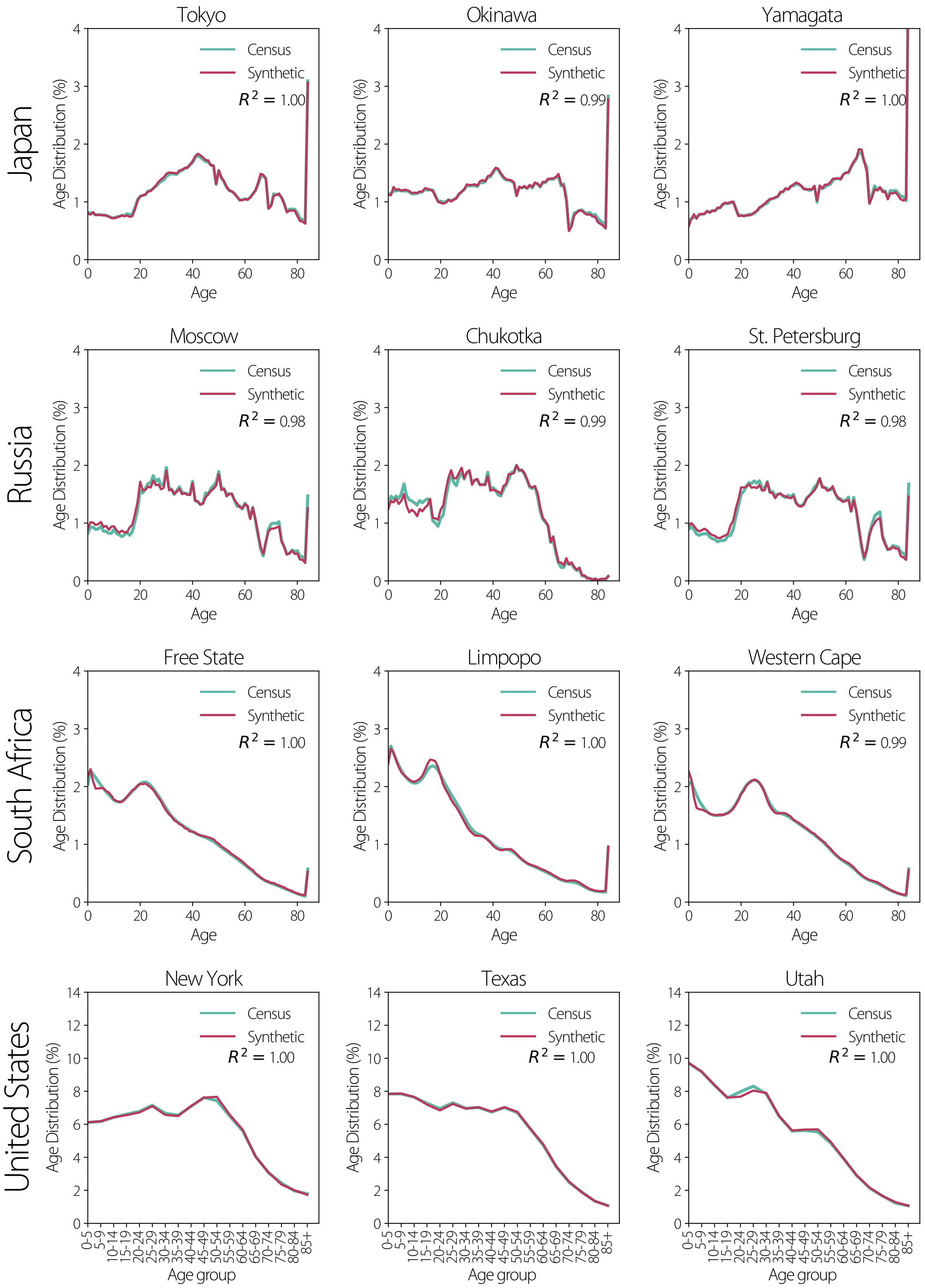

Supplementary Figure 2: **Age Distributions** Comparison of the census and synthetic age distributions for 3 subnational locations in Japan (first row), Russia (second row), South Africa (third row), and the United States (last row).

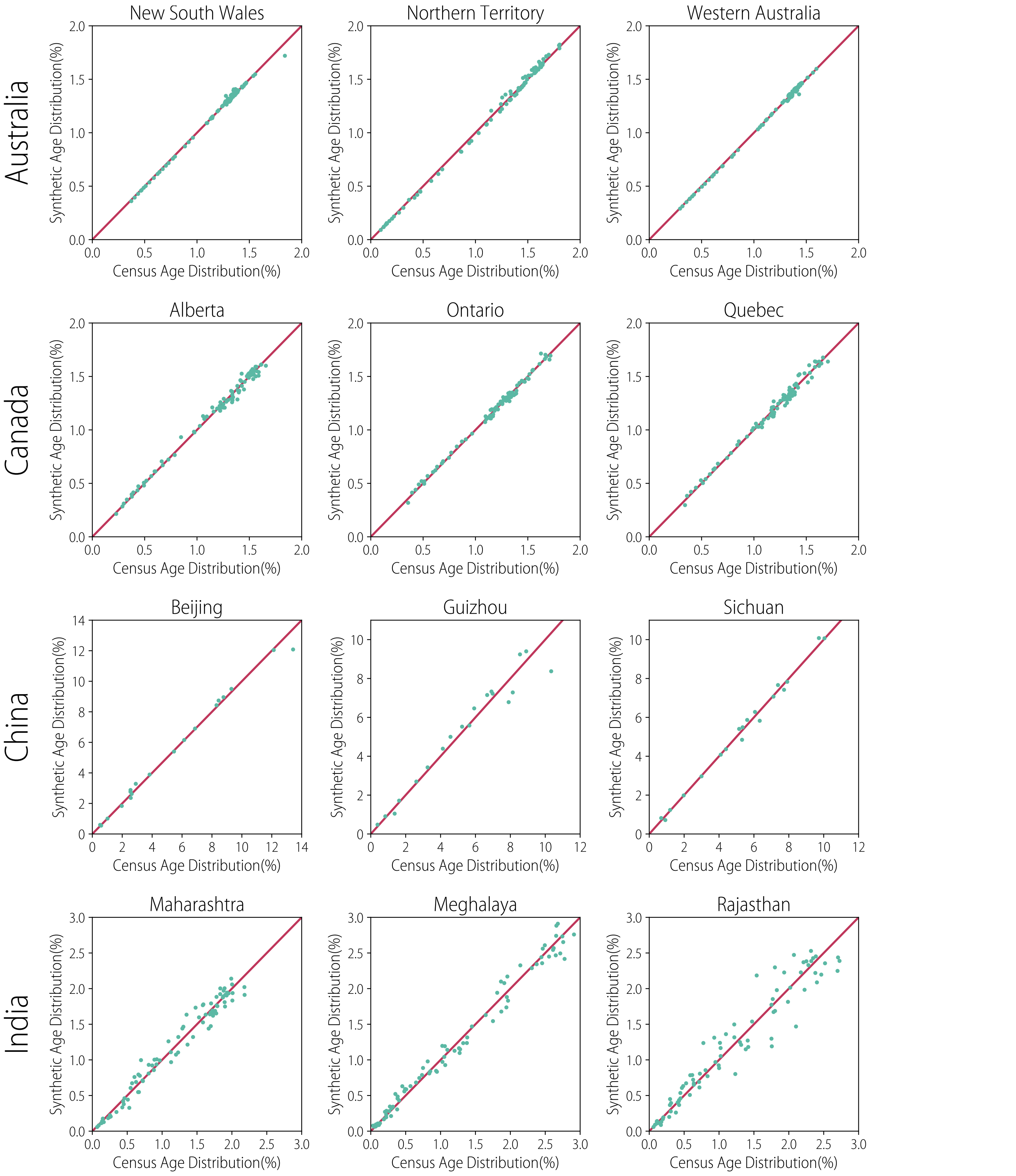

Supplementary Figure 3: **Age Distributions** Correlation of the census and synthetic age distributions for 3 subnational locations in Australia (first row), Canada (second row), China (third row), and India (last row).

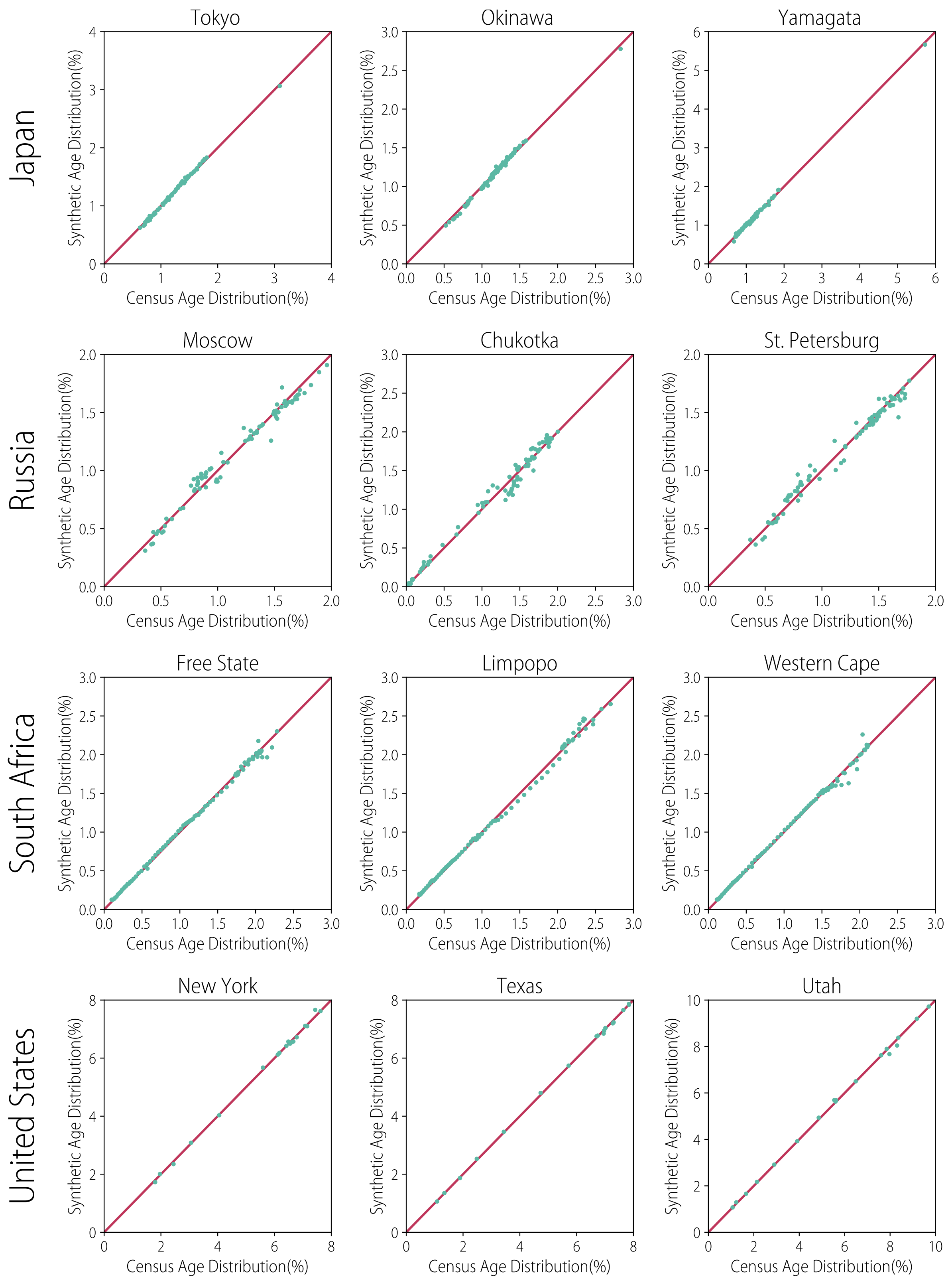

Supplementary Figure 4: **Age Distributions** Correlation of the census and synthetic age distributions for 3 subnational locations in Japan (first row), Russia (second row), South Africa (third row), and the United States (last row).

We can also compare the census and synthetic distributions for the household size. Table 3 reports the goodness of fit between the census and synthetic household size distributions for a sample of subnational locations. Figures 5-6 show a comparison of the census and synthetic household size distributions for this sample of subnational locations. We report the goodness of fit of the census and synthetic household size distributions for all 277 subnational locations and Israel in the Appendix (see Tables 8-9).

Household Size Distributions: Census vs. Synthetic Pop. Statistical Tests

Location	Country	Pearson's r	KS	RMSE
New South Wales	AUS	1.00	0.12	0.01
Northern Territory	AUS	1.00	0.25	0.83
Queensland	AUS	1.00	0.12	0.02
Alberta	CAN	1.00	0.00	0.00
Ontario	CAN	1.00	0.00	0.00
Quebec	CAN	1.00	0.00	0.00
Beijing	CHN	1.00	0.10	0.00
Guizhou	CHN	1.00	0.10	0.00
Sichuan	CHN	1.00	0.10	0.00
Meghalaya	IND	1.00	0.11	0.00
Maharashtra	IND	1.00	0.11	0.00
Rajasthan	IND	1.00	0.11	0.00
Israel	ISR	1.00	0.14	0.16
Tokyo	JPN	0.91	0.20	42.65
Okinawa	JPN	0.99	0.10	2.79
Yamagata	JPN	1.00	0.20	2.12
Moscow	RUS	1.00	0.20	0.00
Chukotka	RUS	1.00	0.20	0.02
St. Petersburg	RUS	1.00	0.10	0.01
Free State	ZAF	1.00	0.10	0.01
Limpopo	ZAF	1.00	0.10	0.02
Western Cape	ZAF	1.00	0.20	0.03
New York	USA	1.00	0.14	0.00
Texas	USA	1.00	0.14	0.00
Utah	USA	1.00	0.14	0.00

Supplementary Table 3: Table of statistical tests comparing the census and synthetic population household size distributions for a sample of subnational locations. Country codes are used to refer to the country name (see Table 1 for the country codes).

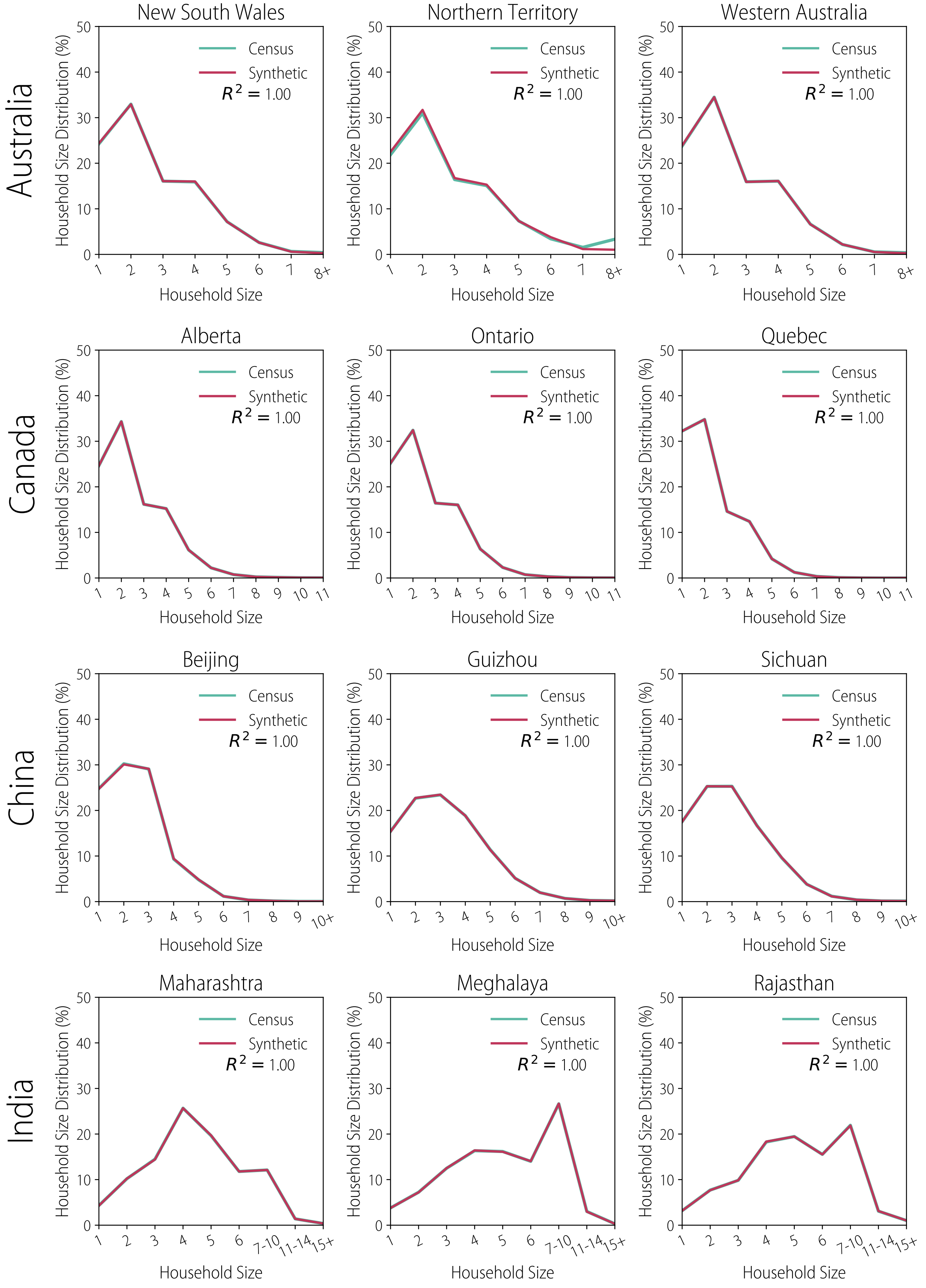

Supplementary Figure 5: **Household Size Distributions** Comparison of the census and synthetic age distributions for 3 subnational locations in Australia (first row), Canada (second row), China (third row), and India (last row).

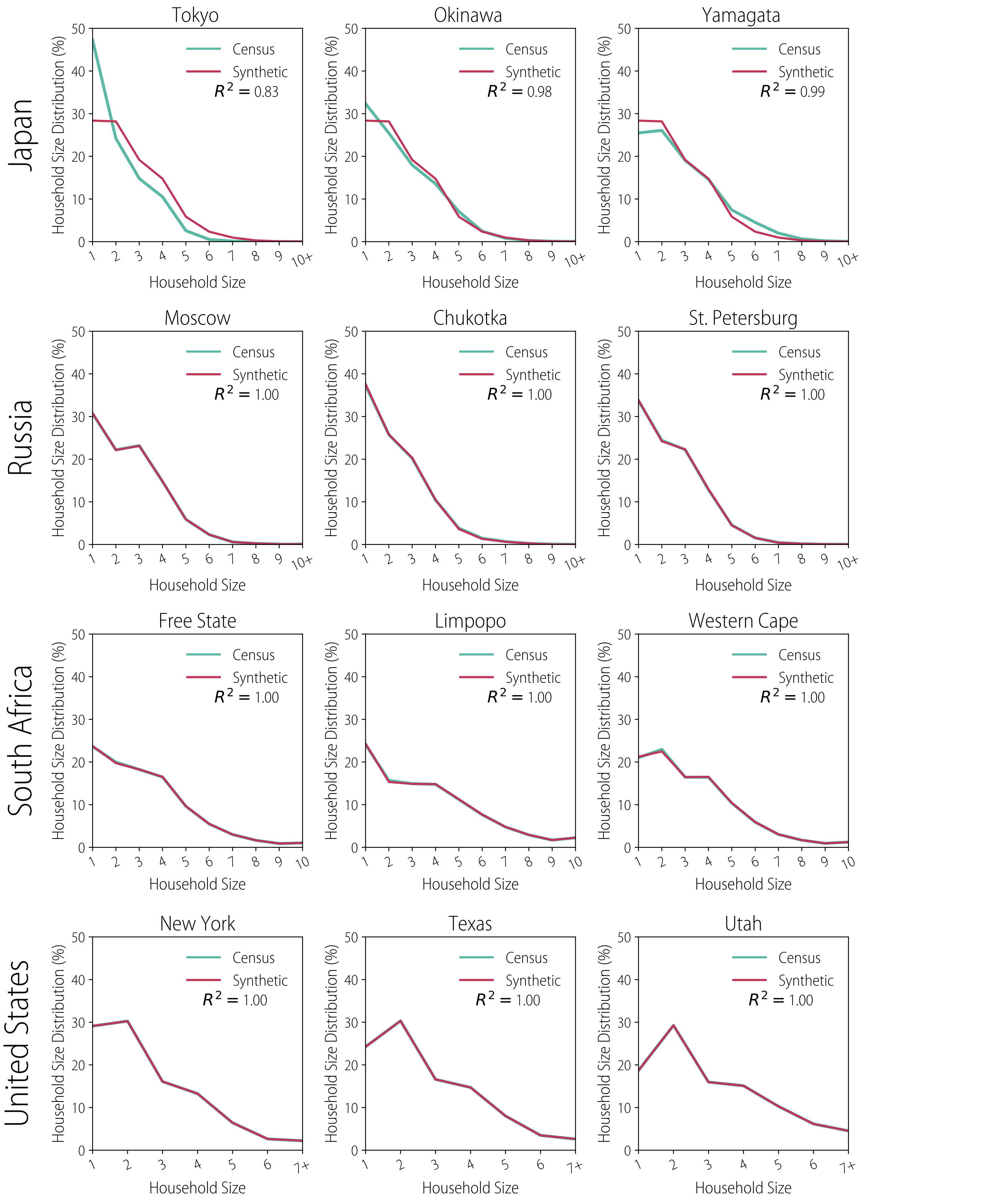

Supplementary Figure 6: **Household Size Distributions** Comparison of the census and synthetic age distributions for 3 subnational locations in Japan (first row), Russia (second row), South Africa (third row), and the United States (last row).

For each country, other sociodemographic distributions were available but not used explicitly in the algorithm generating the synthetic populations. As an example, for the country of India, we also had available to us the census distribution of the number of couples per household. As this data was not used in the generating procedure, we can use this distribution to evaluate the ability of our algorithm to generate realistic synthetic households in that country. Table 4 reports the results of the aforementioned statistical tests between the synthetic and census distributions on the number of couples per household throughout the states and territories of India.

As a further validation, we perform a comparison between the age-profile by household size resulting from the synthetic population and those reported in the data for Russia, which is the only analyzed non-European country for which this data is reported in the census. Fig. 50-54 (in Appendix) highlight an excellent agreement between the data and the synthetic populations.

No. of Couples per Household in India: Census vs. Synethetic Population Statistical Tests

Location	Pearson's r	KS	RMSE
Andaman & Nicobar Islands	0.9989	0.3333	0.0002
Andhra Pradesh	0.9997	0.1667	0.0001
Arunachal Pradesh	0.9940	0.3333	0.0009
Assam	0.9963	0.1667	0.0005
Bihar	0.9990	0.1667	0.0001
Chhattisgarh	0.9956	0.3333	0.0006
Daman & Diu	0.9991	0.1667	0.0001
Goa	0.9987	0.1667	0.0003
Gujarat	0.9961	0.1667	0.0009
Haryana	0.9961	0.1667	0.0007
Himachal Pradesh	0.9997	0.3333	7.2e-5
Jammu & Kashmir	0.9984	0.1667	0.0004
Jharkhand	0.9966	0.1667	0.0004
Karnataka	0.9991	0.1667	0.0001
Kerala	0.9998	0.1667	8.5e-5
Madhya Pradesh	0.9994	0.1667	0.0001
Maharashtra	0.9998	0.1667	7.6e-5
Manipur	0.9951	0.3333	0.0015
Meghalaya	0.9809	0.1667	0.0025
Mizoram	0.9995	0.1667	0.0001
Nagaland	0.9989	0.3333	0.0002
NCT of Delhi	0.9984	0.3333	0.0003
Odisha	0.9995	0.1667	8.3e-5
Puducherry	0.9992	0.1667	0.0001
Punjab	0.9979	0.1667	0.0004
Rajasthan	0.9958	0.1667	0.0006
Sikkim	0.9981	0.1667	0.0002
Tamil Nadu	0.9923	0.3333	0.0030
Tripura	0.9991	0.1667	0.000
Uttar Pradesh	0.9969	0.1667	0.0006
Uttarakhand	0.9991	0.1667	9.9e-5
West Bengal	0.9973	0.1667	0.0004

Supplementary Table 4: Table of statistical tests comparing the census and synthetic population distribution of the no. of couples in per household for locations throughout India.

Algorithm 2 Synthetic Schools, Part I

procedure ASSIGN STUDENTS
  for $i \leftarrow 1$ **to** N **do**
    $a^i \leftarrow$ age(person i)
    $p_s^i \leftarrow$ enrollment rate for age a^i
    $v^i \leftarrow$ Sample $\mathcal{M}(v|p_s^i)$ to determine if a^i is a student
    if ($v^i == \textit{student}$) **then**
      $g^i \leftarrow$ Sample $\mathcal{M}(g|a^i)$ for the grade of a^i
      $s_c^i \leftarrow$ Sample $\mathcal{M}(s_c|g^i)$ for school type conditional on g^i
      $s_s^i \leftarrow$ Sample $\mathcal{M}(s_s|s_c^i)$ for school size dependent† on s_c^i
      if a school of max. size s_s^i and type s_c^i exists **then**
        if size(school) $< s_s^i$ **then**
          add student with a^i to the school
        else
          create a new school and add a^i to the school‡

†In some cases there is no data relating school size to any other characteristics of schools such as the school type. In that case, we sample school size independently.

‡When creating schools, we often find that the last schools created are filled with far fewer students than expected from s_s^i, the sampled school size. These schools are typically on the order of only a handful of students. To adhere to the school size distribution given by the data we do the following. Once all students have been placed in an initial school we redistribute the students in these much smaller than expected schools to other schools of the same type that are much larger in size. This process allows us to avoid over generating extremely small schools while maintaining an approximate match to the school size distribution.

Scheme S2: Algorithm 2 to build synthetic schools

1.3 Adaptive algorithms for the development of synthetic schools and workplaces

With the synthetic population generated in the household setting, a similar procedure is used to assign those individuals to their respective schools and workplaces based on enrollment and employment records. These records detail the enrollment and employment rates by age, institutional sizes and their age structures, as well as the student-to-teacher ratios for different stages of education (i.e., elementary, secondary, tertiary, etc.). Returning to the example of Maharashtra, India, here we outline the procedure to reconstruct the school environment. For locations throughout India there exists a wealth of information on the educational units and the number of attendees by age and grade of school for each state and territory (barring remote and isolated communities such as the Sentinelese of the Andaman & Nicobar Islands, for whom less data is available) available through the Unified District Information System for Education (U-DISE) database [10, 11]. For example, U-DISE report on Maharashtra provides a table of student enrollment by grade conditional on the age of the students. We use this to produce a multinomial distribution $\mathcal{M}(g|a^i)$ to describe the probability a student of age a^i is in grade g. The same report also provides a table of student enrollment by grade and school type. The U-DISE reports reveal a surprising mix of school types which students attend: primary, primary with upper primary, primary with upper primary and secondary and higher secondary, upper primary only, upper primary with secondary and higher secondary, secondary only, secondary with higher secondary, higher secondary only. From this we form the multinomial distribution $\mathcal{M}(s_c|g)$ which describes the probability for a student in grade g to attend a school of type s_c. We then combine the two multivariate joint distributions to produce a multinomial distribution of enrollment in school type s_c by age a^i, $\mathcal{M}(s_c|a^i)$. A third table from the report provides the number of schools with enrollment size s_s, which we use to form the independent multinomial distribution of school sizes $\mathcal{M}(s_s)$. In addition to this the census of India website offers for each location the enrollment numbers by age, which we use to construct a simple probability $\mathcal{P}(a)$ of school enrollment for each age a^i.

Algorithm 3 Synthetic Workplaces, Part I

procedure GET WORKERS w
  for $i \leftarrow 1$ **to** N **do**
    $a^i \leftarrow$ age(person i)
    $p_w^i \leftarrow$ employment rate for age a^i
    $w^i \leftarrow$ Sample $\mathcal{M}(w|p_w^i)$ to determine if a^i is a worker
    if $(w^i == worker)$ **then**
      add a_i to set of all workers w

Scheme S3: Algorithm 3 to get the set of all workers

Algorithm 4 Synthetic Schools, Part II

procedure ASSIGN TEACHERS TO N_S SCHOOLS
  for $s \leftarrow 1$ **to** N_S **do**
    $r^s \leftarrow$ Sample $\mathcal{M}(r|s_c)$ for the student-teacher ratio r^s conditional on s_c
    $n_t \leftarrow$ **ceil**(s_s/r_s) for the number of teachers at school$s$
    for $i \leftarrow 1$ **to** n_t **do**
      $a_t^i \leftarrow$ Choose teacher from set of all workers above min. age of teachers $w(a > a_{t_{min}})$
      add teacher with a_t^i to school s
      subtract 1 from count of workers of age a^i

Scheme S4: Algorithm 4 to assign teachers to schools from workers

Once the data needed to assign students to schools is prepared, the algorithm for generating schools then goes as follows. For each individual in the synthetic population we use $\mathcal{P}(a)$ to sample whether they are enrolled in school conditional on their age a^i. We use $\mathcal{M}(s_c|a^i)$ to assign the student to a school of type s_c, and then add the student to a specific school of type s_c with size s_s sampled using $\mathcal{M}(s_s)$. Once a school of type s_c has reached its maximum size s_s, we open a new school of this type for students to attend and continue adding new schools as needed. Scheme S2 shows a sketch of this algorithm to build synthetic schools.

Having gone through the synthetic population and assigning them to the school as students, we next assign teachers to each school. We use governmental information on the student-to-teacher ratios depending on school size and school type (i.e, elementary, secondary, tertiary, etc.) to sample the number of teachers for each of the generated schools. First, we go through the synthetic population again and use employment records provided by the census to sample the probability for an individual of age a_i to be employed. If employed and above an age threshold for teachers [10, 11], then the individual is selected to be a teacher at one of the generated schools as needed (see Schemes S3 and S4 for a sketch of this process).

Finally, for all other individuals in the synthetic population we continue to use employment records to sample the probability of their employment. We then use data on firm or workplace sizes to sample the size of the workplace s_w for each employed individual and assign them to a workplace of that size (see Scheme S5 for a sketch of the algorithm). We report the goodness of fit between the census and synthetic enrollment and employment rates by age for all subnational locations in this study, in the Appendix (see Tables 10,11, and 12,13, respectively).

Employed and partially employed students and partially employed workers

In the procedure of reconstructing a synthetic population individuals are generally treated as either enrolled in school as students, or employed as workers. In reality, these two roles may overlap for a number of individuals as people may be students and workers at the same time. From the data available to us, this phenomenon appears to be relevant at least for some countries and locations, in particular for populations throughout Australia, Canada, and the United States

Algorithm 5 Synthetic Workplaces, Part II

```
procedure ASSIGN NON-TEACHING WORKERS
    for i in w* do
        a^i ← age(worker i)
        w_s^i ← Sample M(w_s) for workplace size† w_s^i
        if a workplace of max. size w_s^i exists then
            if size(workplace) < w_s^i then
                add worker with a^i to the workplace
            else
                create a new workplace of max. size w_s^i and add a^i to the workplace
```

Scheme S5: Algorithm 5 to build synthetic workplaces

[1, 2, 23].

For these three countries we developed a modified procedure to account for the contact with individuals who engage in both activities in a part-time status.

First, our algorithm to generate schools and workplaces samples for each individual whether they are a student, worker, student and worker, or inactive and thus unengaged in these activities.

We assign all individuals sampled to be both students and workers to both schools and workplaces as if that is their only activity and they are full-time in that status. Once we have assigned all individuals to the school and workplace setting, we remove a fraction of individuals $f_{K,i}$ of age i from the setting K for $K \epsilon [S, W]$ to reflect the reduced overall amount of time part-time students or workers spend in each environment.

From the data available to us part-time students and part-time workers spend on average half the amount of time engaged in their activity compared with those who are full-time students or full-time workers, thus their presence in the environment warrants their inclusion in the setting. Thus the fraction $f_{K,i}$ can be expressed as follows,

$$f_{K,i} = 1 - \frac{p_{K,i}^{FT} + \frac{1}{2} p_{K,i}^{PT}}{p_{K,i}^{FT} + p_{K,i}^{PT}} \tag{1}$$

where $p_{K,i}^{FT}$ and $p_{K,i}^{PT}$ is the percentage of individuals of age i with full-time status and part-time in setting K, respectively. We multiply the percentage of those with part-time status by a factor of $\frac{1}{2}$ in the numerator to reflect the amount of time they spend in the setting K relative to full-time colleagues. Then, for each school or workplace network we remove individuals of age i from the network with probability $f_{K,i}$. From the new schools and workplaces with reduced networks we then recalculate the setting specific contact matrix in these two settings and use these matrices to describe the age-specific contact patterns in the school and work environment. We then replace the original setting matrices with the newly constructed ones in the formulation of the matrix describing the overall number of contacts between different ages (see next Section).

2 Contact Matrices

2.1 Contact matrices by setting

Figures 7-13 depict the contact matrices by setting for several locations throughout the different countries in this study, at both the national and subnational resolution.

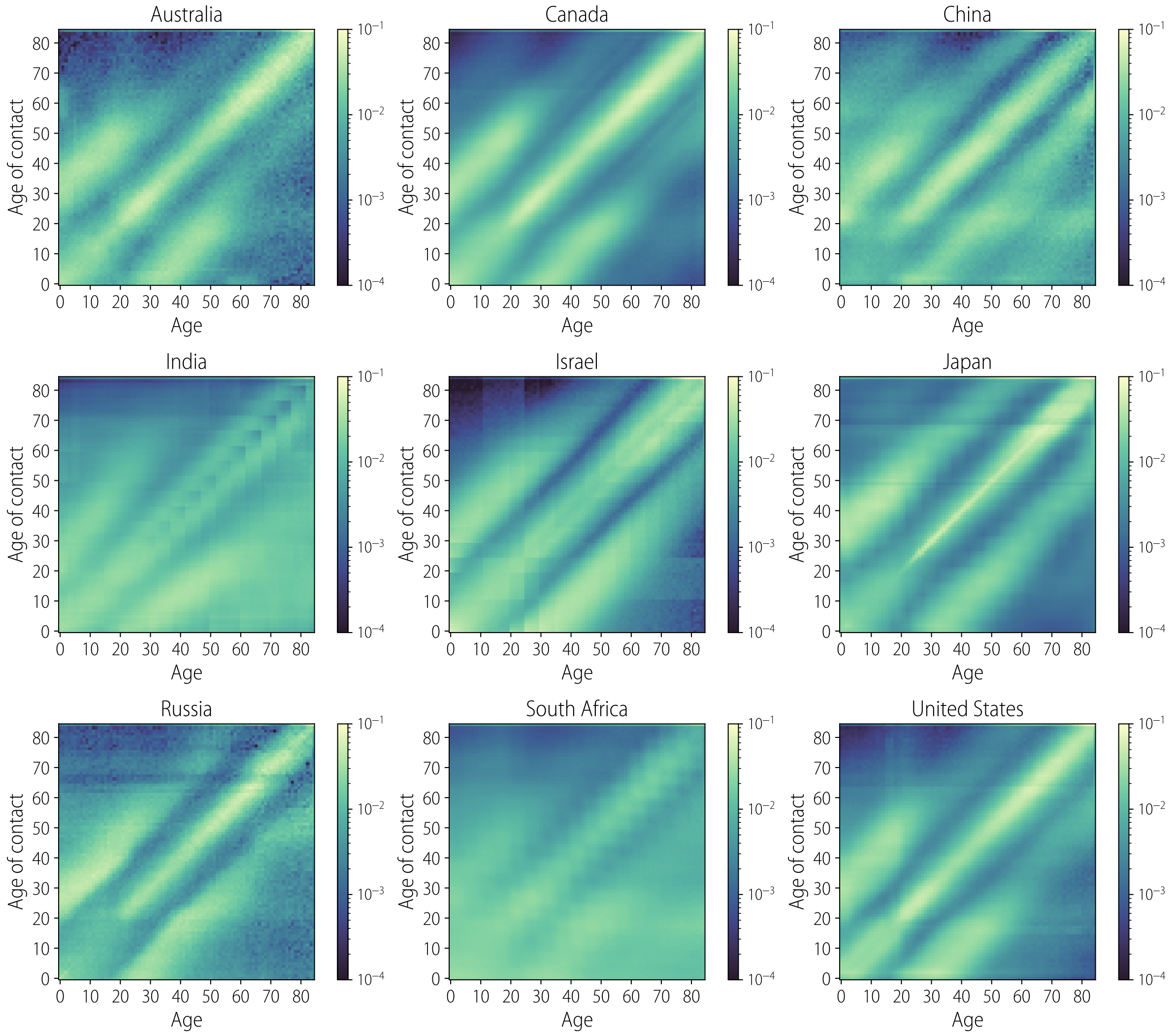

Supplementary Figure 7: **Age mixing patterns in Households** Each heatmap represents the average frequency of contact between an individual of a given age (x-axis) and their possible contacts (y-axis) in each country at the national scale.

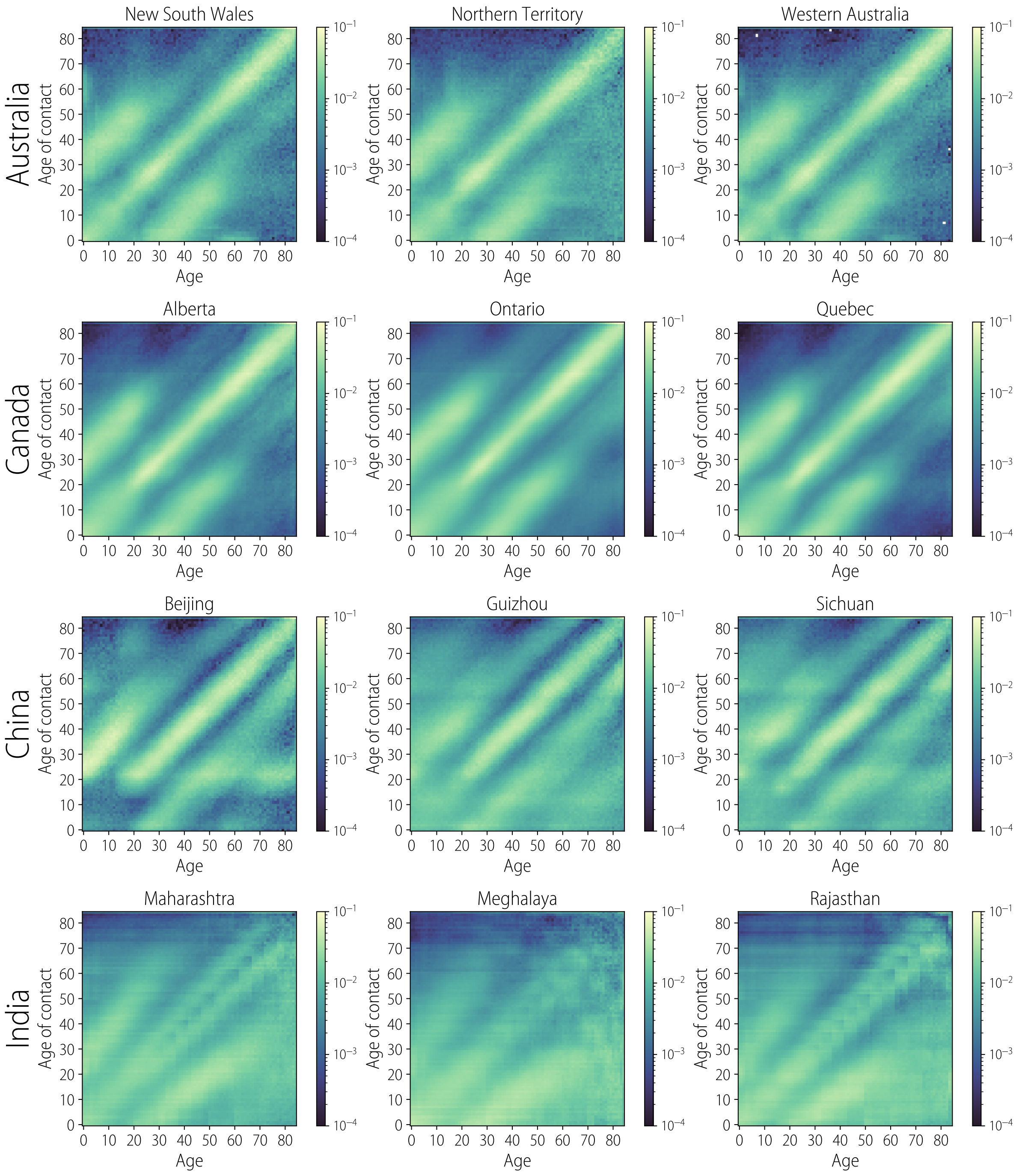

Supplementary Figure 8: **Age mixing patterns in Households** Each heatmap represents the overall average frequency of adequate contacts for influenza transmission by age in 3 subnational locations in Australia (first row), Canada (second row), China (third row), and India (last row).

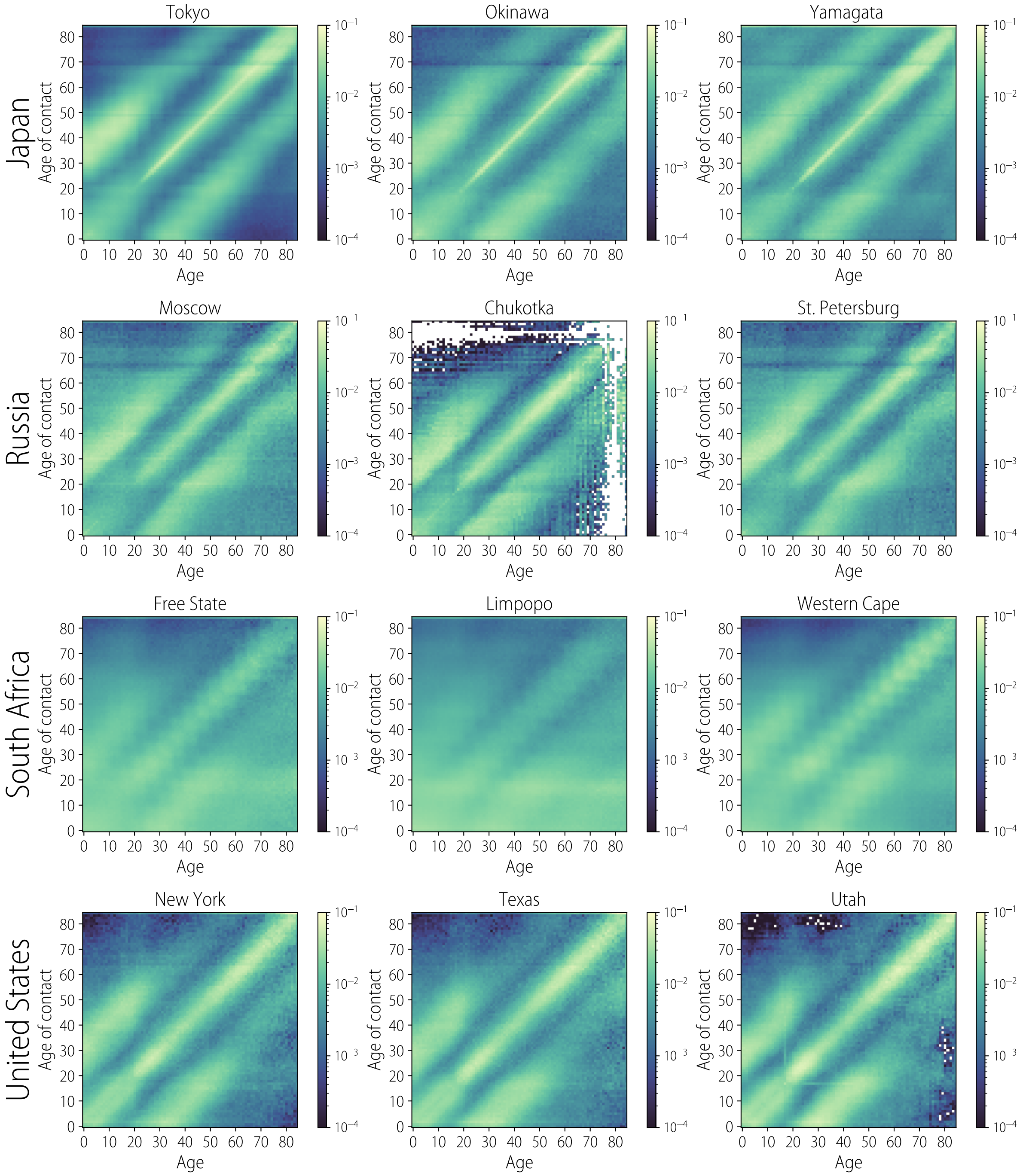

Supplementary Figure 9: **Age mixing patterns in Households** Each heatmap represents the overall average frequency of adequate contacts for influenza transmission by age in 3 subnational locations Japan (first row), Russia (second row), South Africa (third row), and the United States (last row).

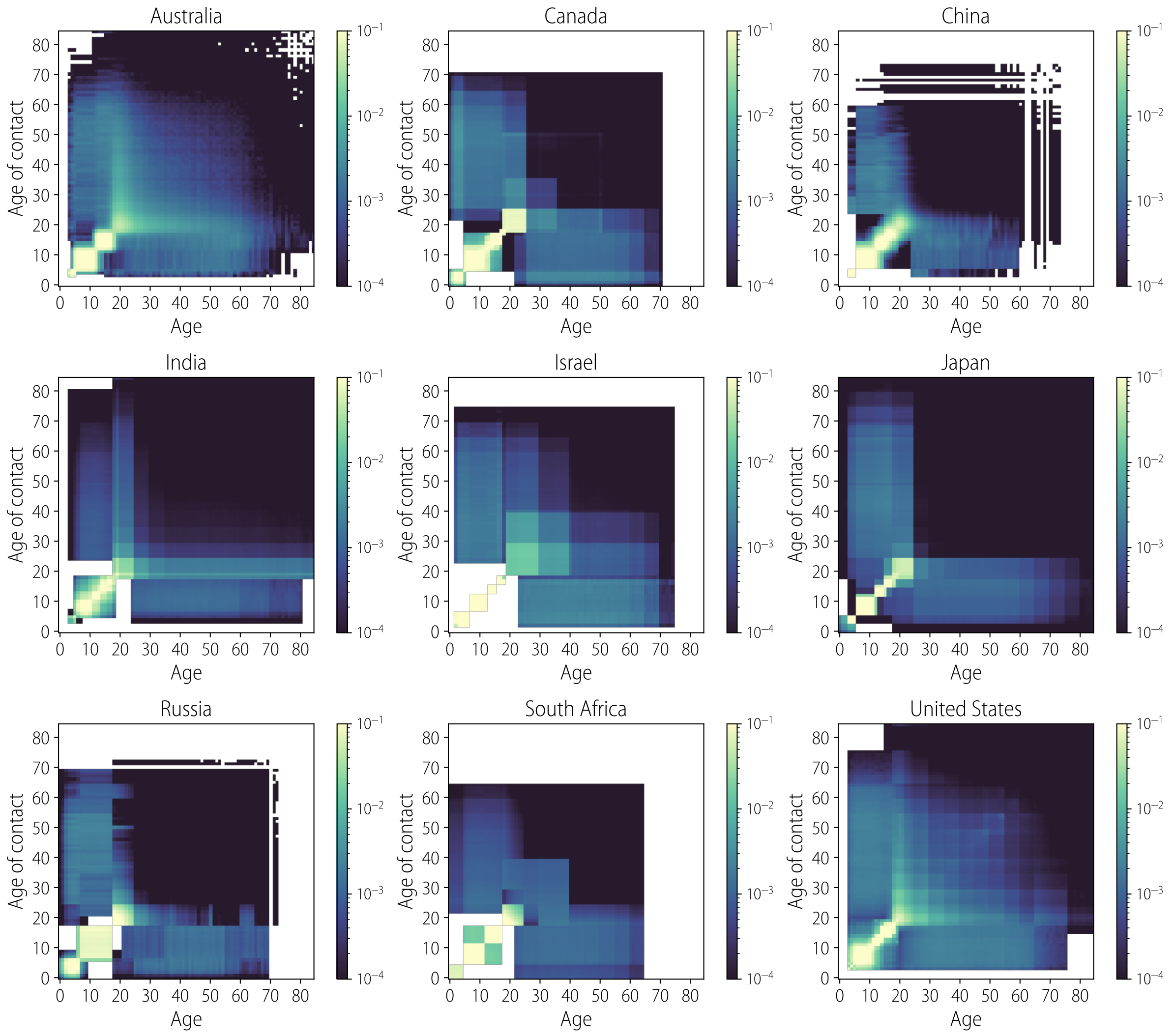

Supplementary Figure 10: **Age mixing patterns in Schools** Each heatmap represents the average frequency of contact between an individual of a given age (x-axis) and their possible contacts (y-axis) in each country at the national scale.

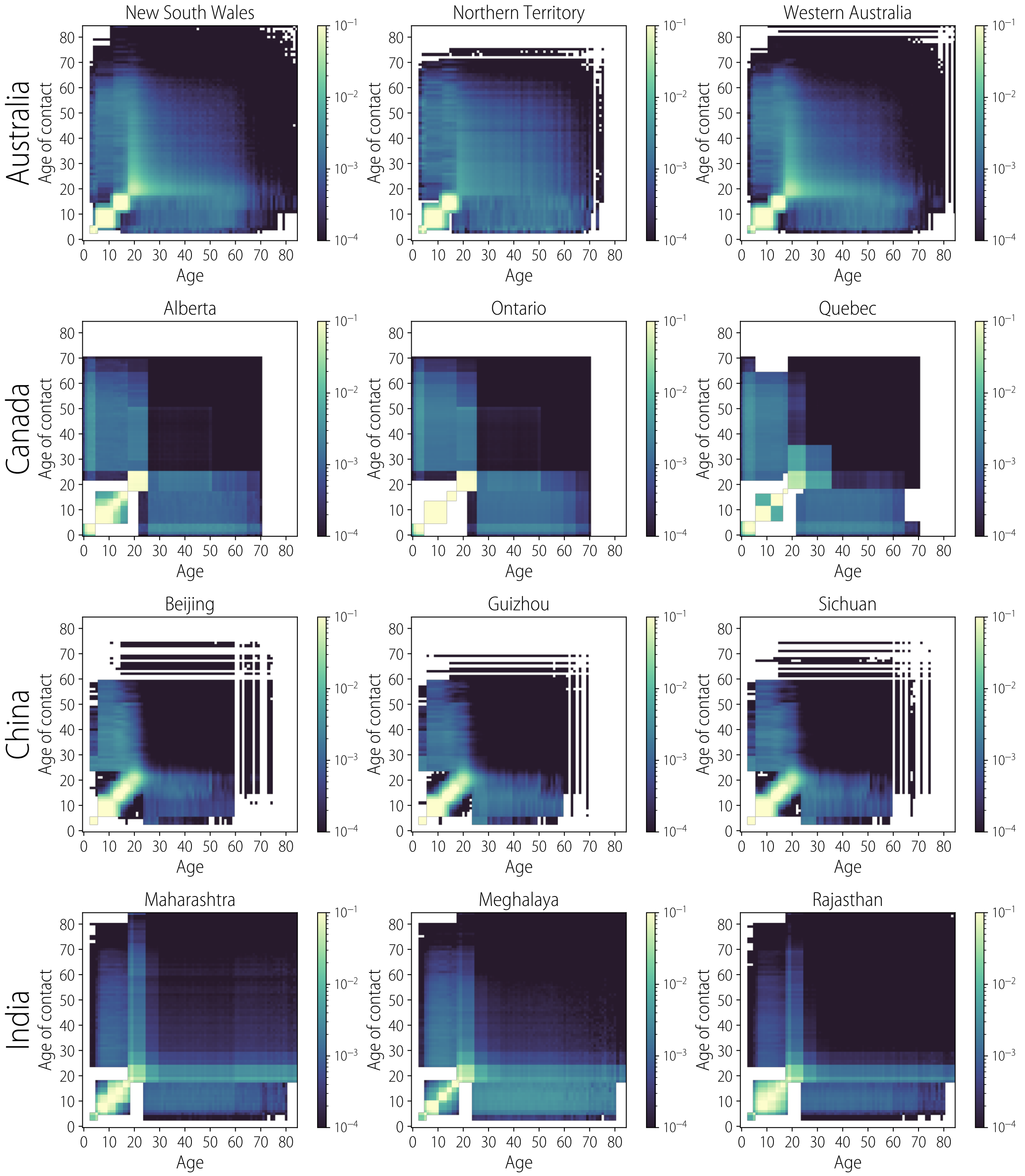

Supplementary Figure 11: **Age mixing patterns in Schools** Each heatmap represents the overall average frequency of adequate contacts for influenza transmission by age in 3 subnational locations in Australia (first row), Canada (second row), China (third row), and India (last row).

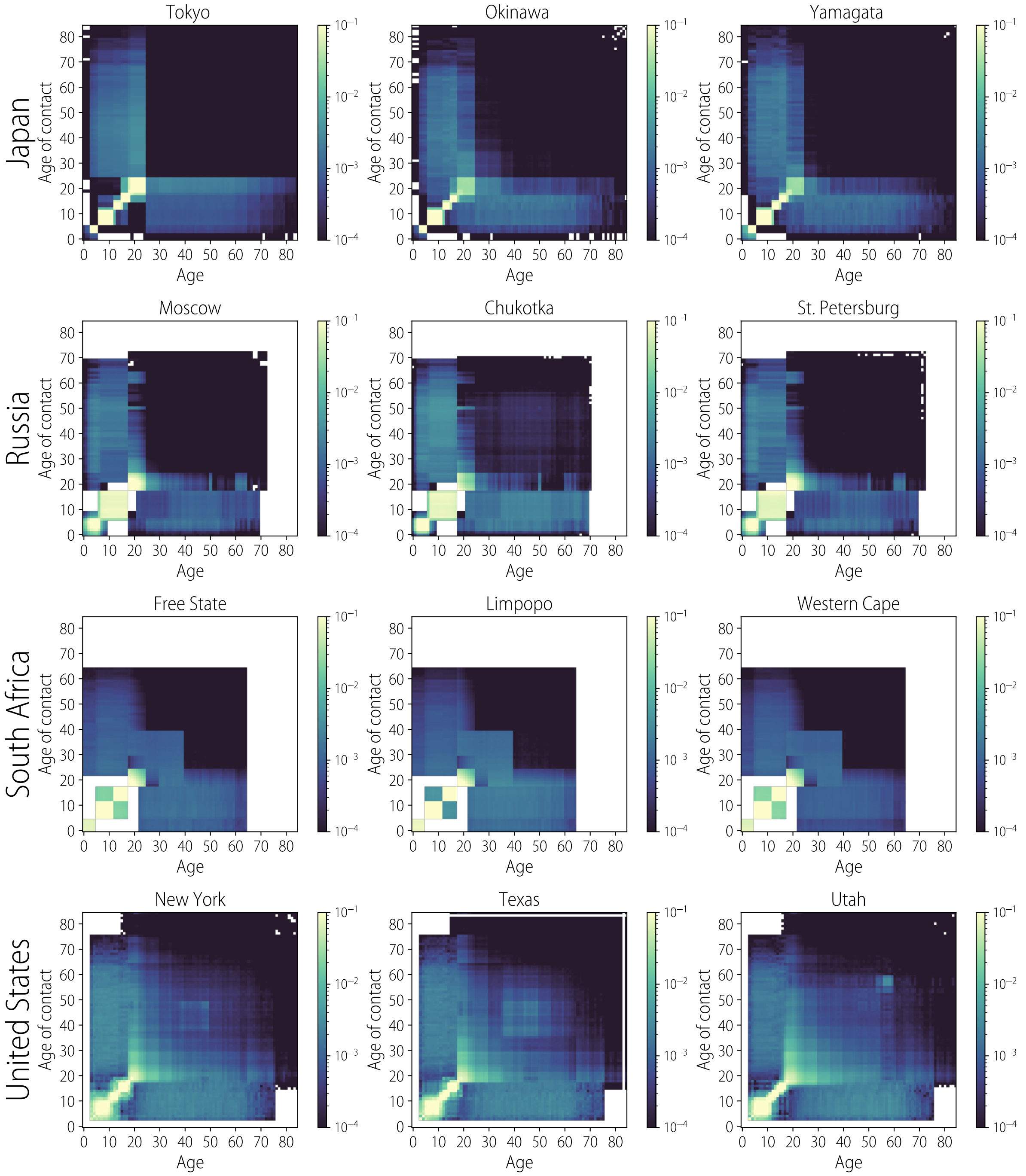

Supplementary Figure 12: **Age mixing patterns in Schools** Each heatmap represents the overall average frequency of adequate contacts for influenza transmission by age in 3 subnational locations Japan (first row), Russia (second row), South Africa (third row), and the United States (last row).

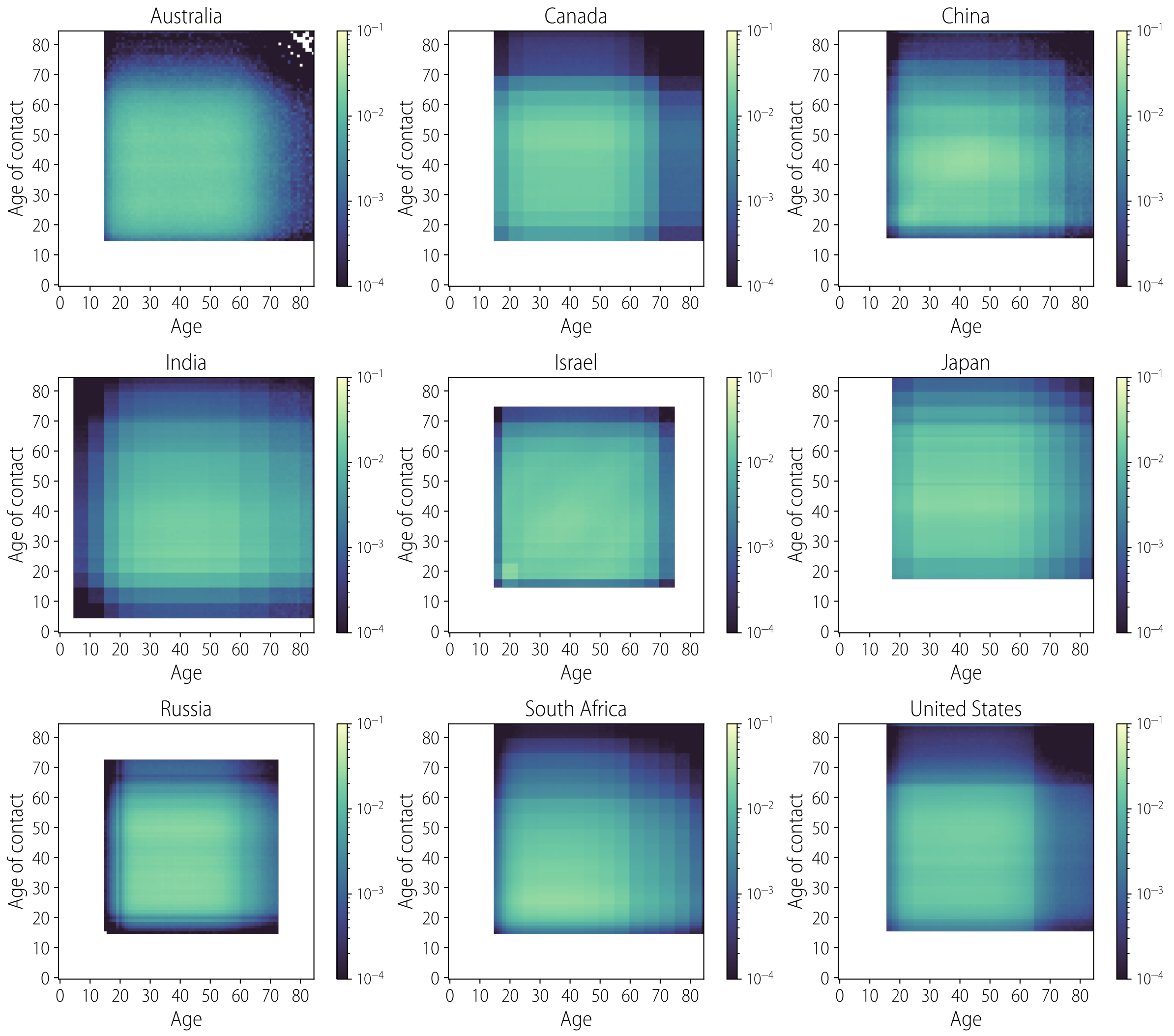

Supplementary Figure 13: **Age mixing patterns in Workplaces** Each heatmap represents the average frequency of contact between an individual of a given age (x-axis) and their possible contacts (y-axis) in each country at the national scale.

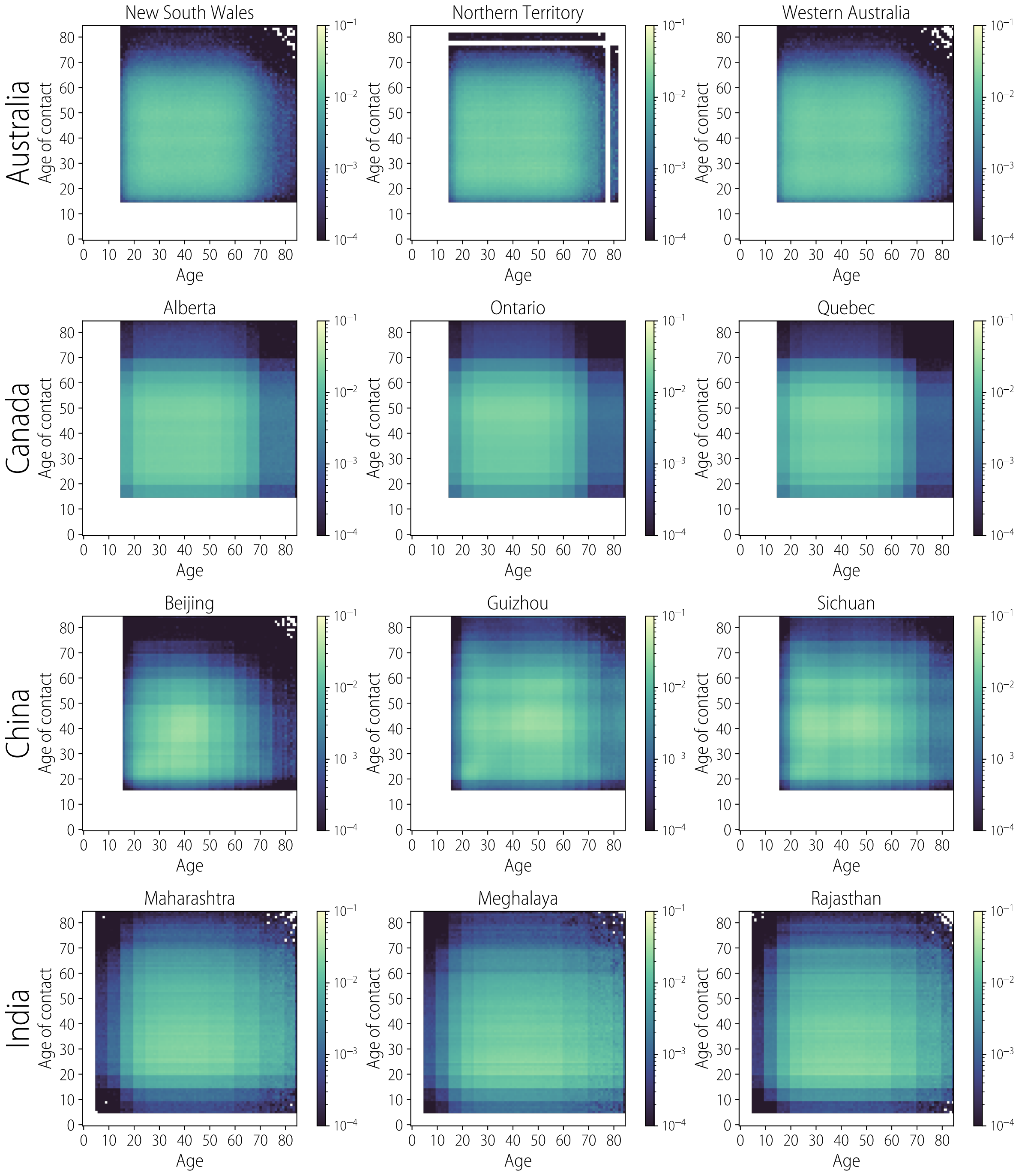

Supplementary Figure 14: **Age mixing patterns in Workplaces** Each heatmap represents the overall average frequency of adequate contacts for influenza transmission by age in 3 subnational locations in Australia (first row), Canada (second row), China (third row), and India (last row).

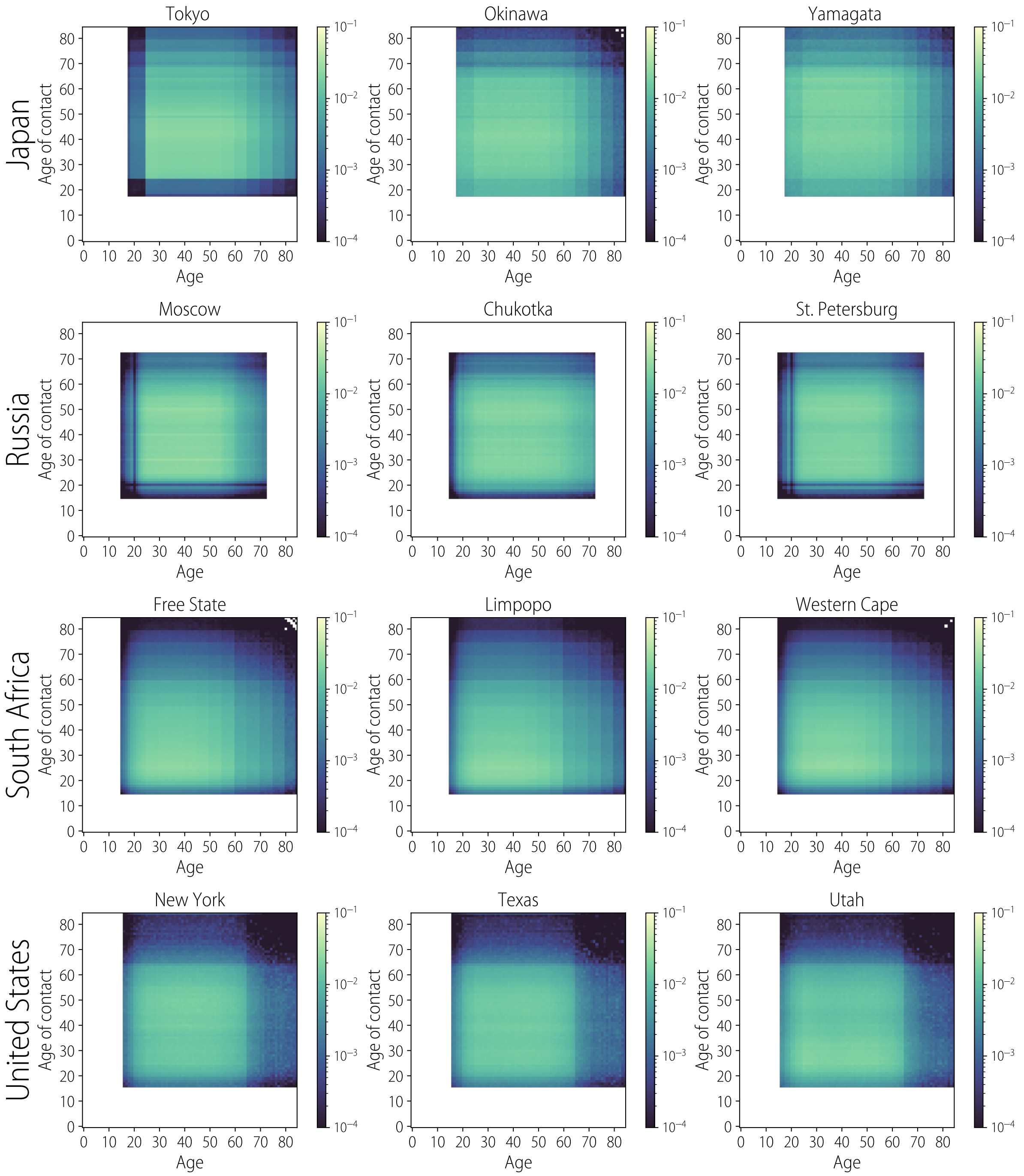

Supplementary Figure 15: **Age mixing patterns in Workplaces** Each heatmap represents the overall average frequency of adequate contacts for influenza transmission by age in 3 subnational locations Japan (first row), Russia (second row), South Africa (third row), and the United States (last row).

2.2 Calibration of the overall contact matrix

We define a matrix of effective contacts for influenza transmission (the overall contact matrix) based on the relative contribution of the households, school, workplace, and general community social setting to the overall frequency of contacts based on diary-based contact survey data. We propose a weighted linear combination of the derived matrices in these four settings, calibrated to match the empirically estimated contact matrices from two contact diary survey studies [26, 27]. Specifically, we use the following seven locations throughout Western Europe and Russia: Finland,

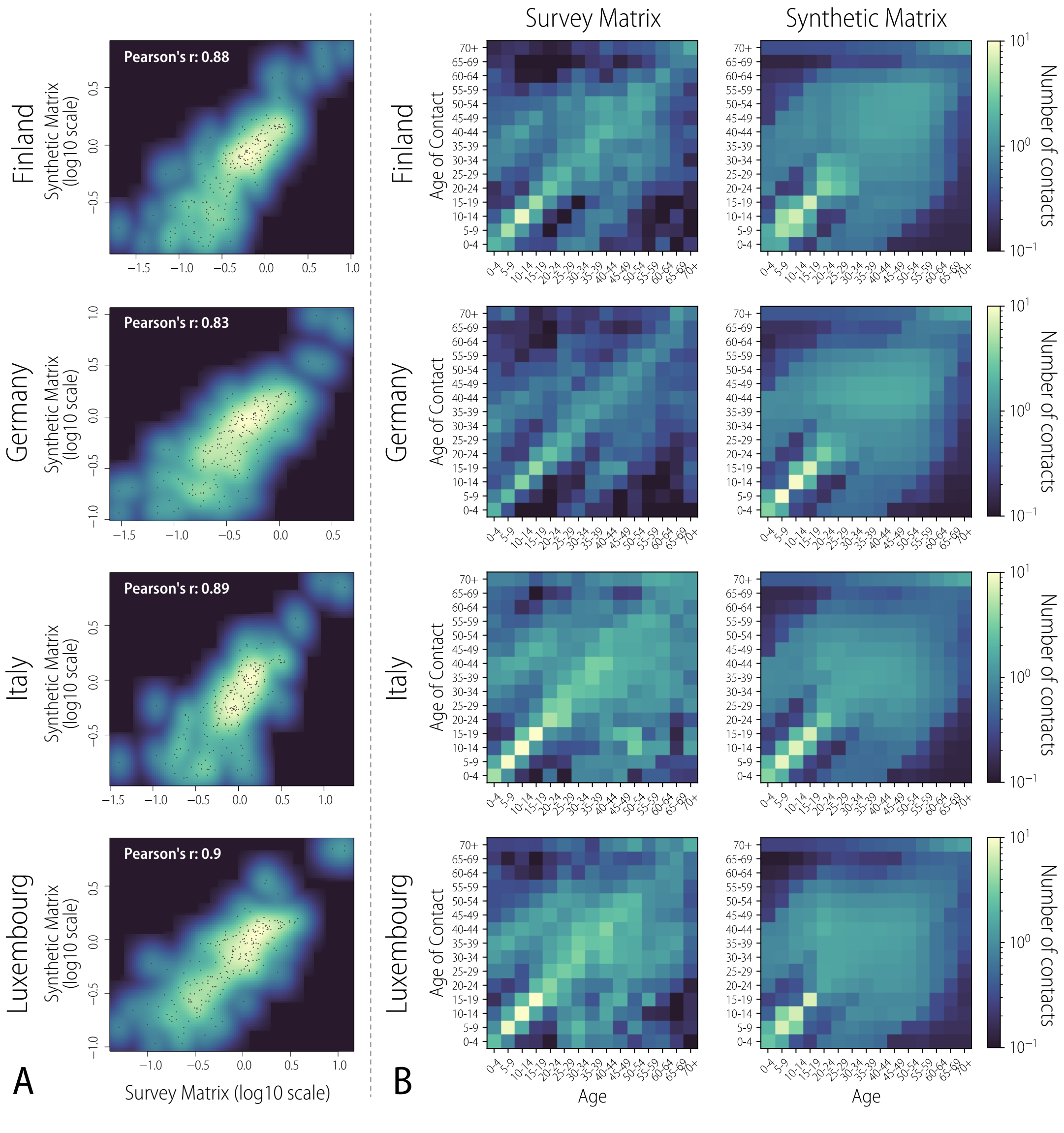

Supplementary Figure 16: **Comparison between the synthetic and diary-based contact matrices. A** Density plots showing the correlation of diary-based contact matrices for four out of seven locations (Finland, Germany, Italy, and Luxembourg) and their respective synthetic contact matrices. The points represent the actual values of the survey and synthetic contact matrices. The linear correlation between the elements of each survey matrix and the corresponding elements of the synthetic matrix is reported in terms of Pearson correlation coefficient, whose values are reported in each plot. **B** Heatmaps representing the diary-based contact matrices and the overall synthetic contact matrices.

Germany, Italy, Luxembourg, The Netherlands, the United Kingdom, and the Tomsk Oblast region of Russia. We perform a multiple linear regression to calibrate the weights w_K of the synthetic setting contact matrices M_{ij}^K such that their linear combination matches the overall contact matrix for all seven locations coming from the survey studies. The weights calibrated in this way are estimated to be [4.11 (standard deviation, sd: 0.41), 11.41 (sd: 0.27), 8.07 (sd: 0.52), 2.79 (sd: 0.48)] for the household, school, workplace, and general community setting.

Figures 16A and 17A show the correlation between the resulting synthetic matrices for the seven locations used in calibration and the available empirical matrices for the same locations. We find significant (p-value < 0.001) Pearson correlations for all seven location, with values ranging from 0.83 to 0.91 for the six European countries and 0.64 for Tomsk Oblast, Russia (see Fig. 16A and 17A). Figures 16B and 17B show a visual comparison between the synthetic and survey matrices, which highlights that the synthetic contact matrices are able to capture some specific features of each location such as contact patterns at school and the relative intensity of the main diagonals.

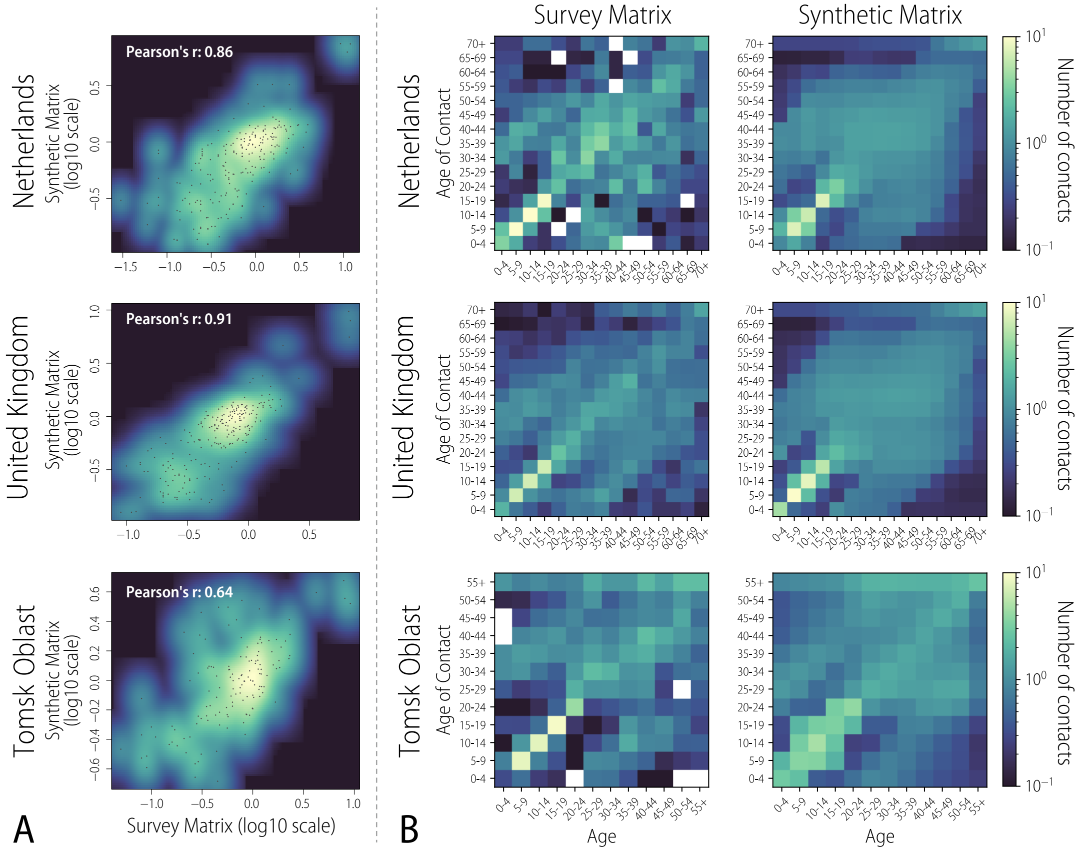

Supplementary Figure 17: **Comparison between the synthetic and diary-based contact matrices (continuation). A** Density plots showing the correlation of diary-based contact matrices for the remaining three out of seven locations (The Netherlands, UK, and Tomsk Oblast of Russia) and their respective synthetic contact matrices. The points represent the actual values of the survey and synthetic contact matrices. The linear correlation between the elements of each survey matrix and the corresponding elements of the synthetic matrix is reported in terms of Pearson correlation coefficient, whose values are reported in each plot. **B** Heatmaps representing the diary-based contact matrices and the overall synthetic contact matrices.

2.3 The overall contact matrix

Figures 18-20 show the calibrated Flu matrices for several locations throughout the different countries in our study, at both the national and subnational resolution.

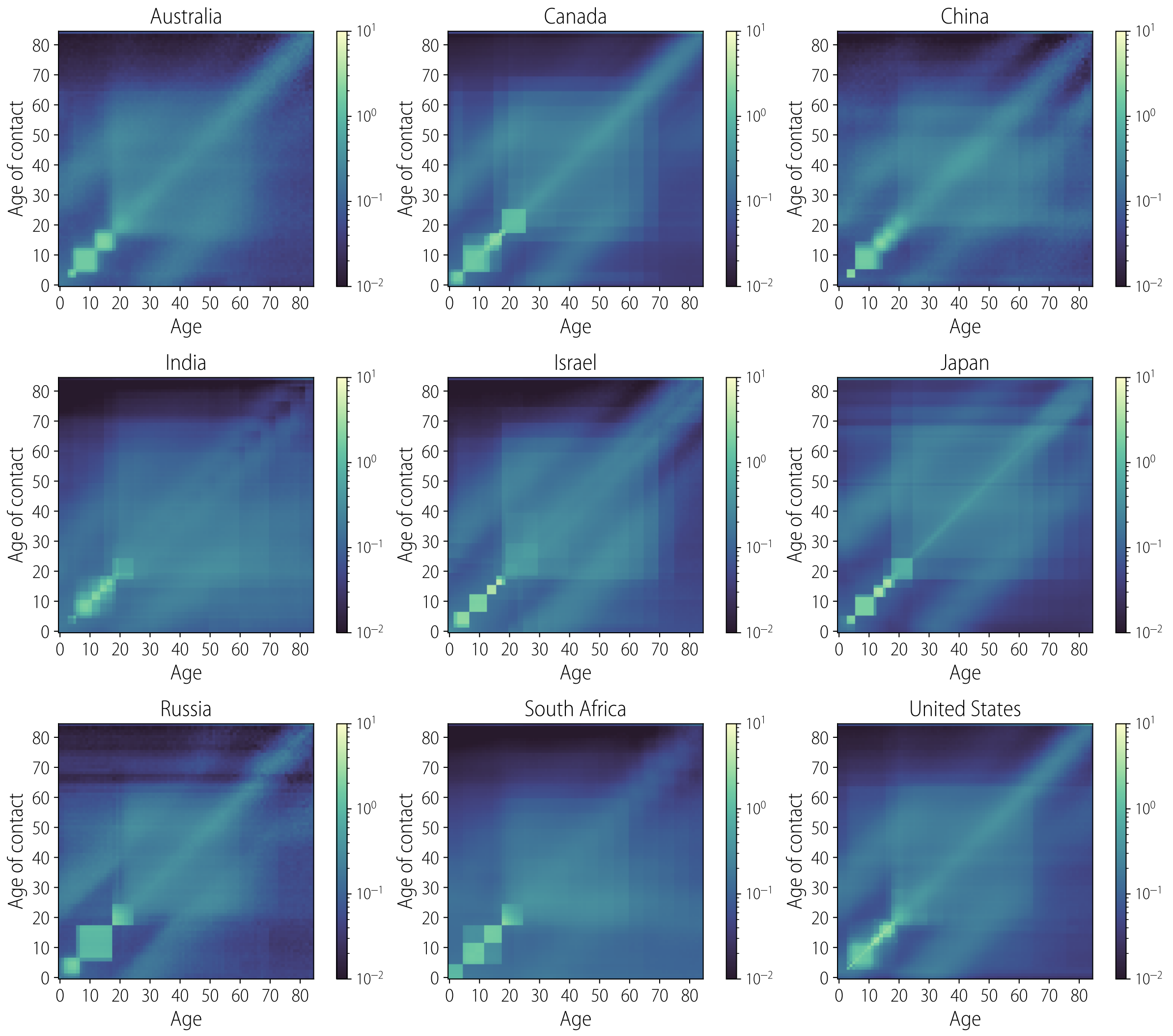

Supplementary Figure 18: **Overall contact matrices.** Each heatmap represents the overall average frequency of adequate contacts for influenza transmission by age in each country at the national scale.

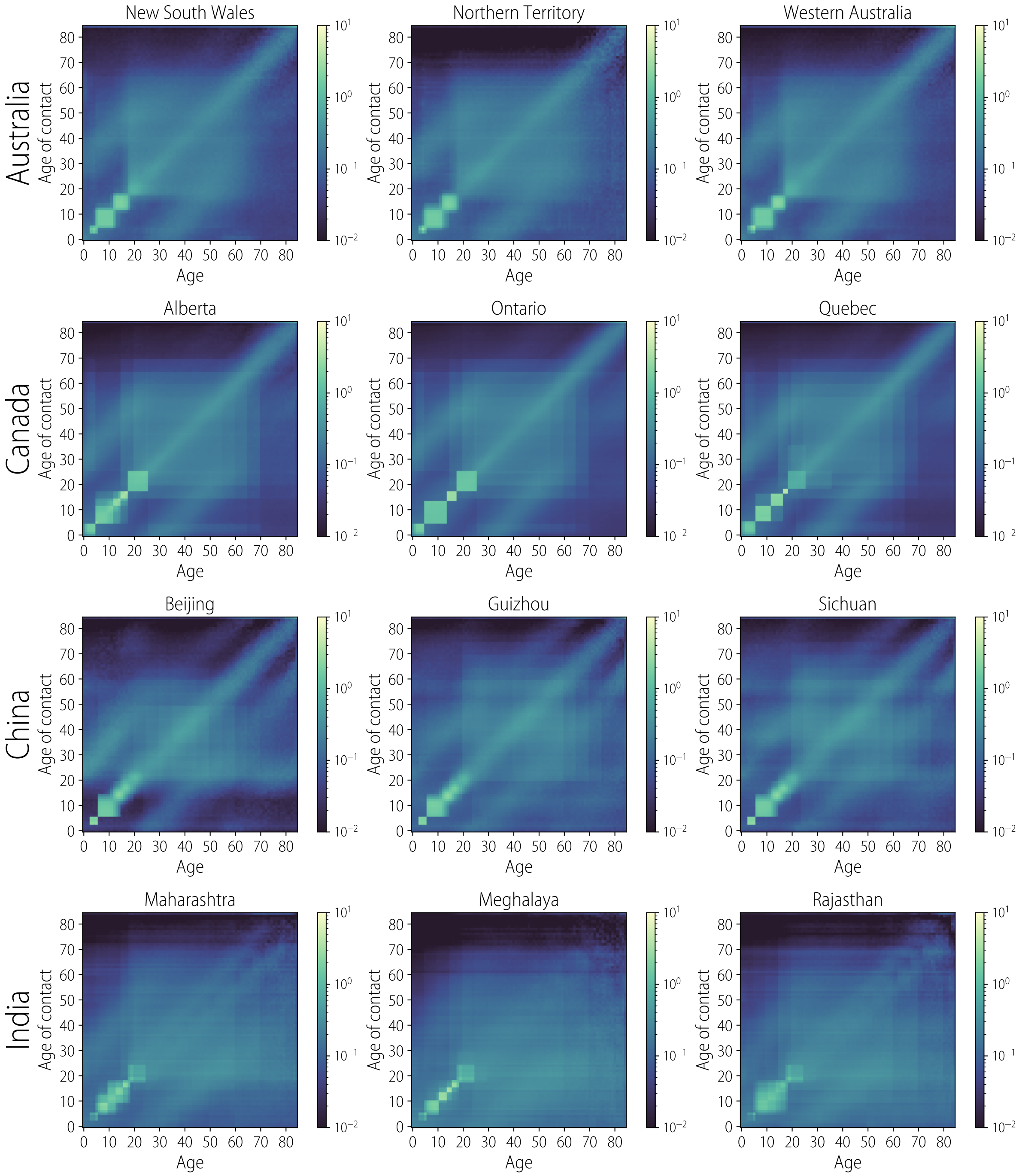

Supplementary Figure 19: **Overall contact matrices.** Each heatmap represents the overall average frequency of adequate contacts for influenza transmission by age in 3 subnational locations in Australia (first row), Canada (second row), China (third row), and India (last row).

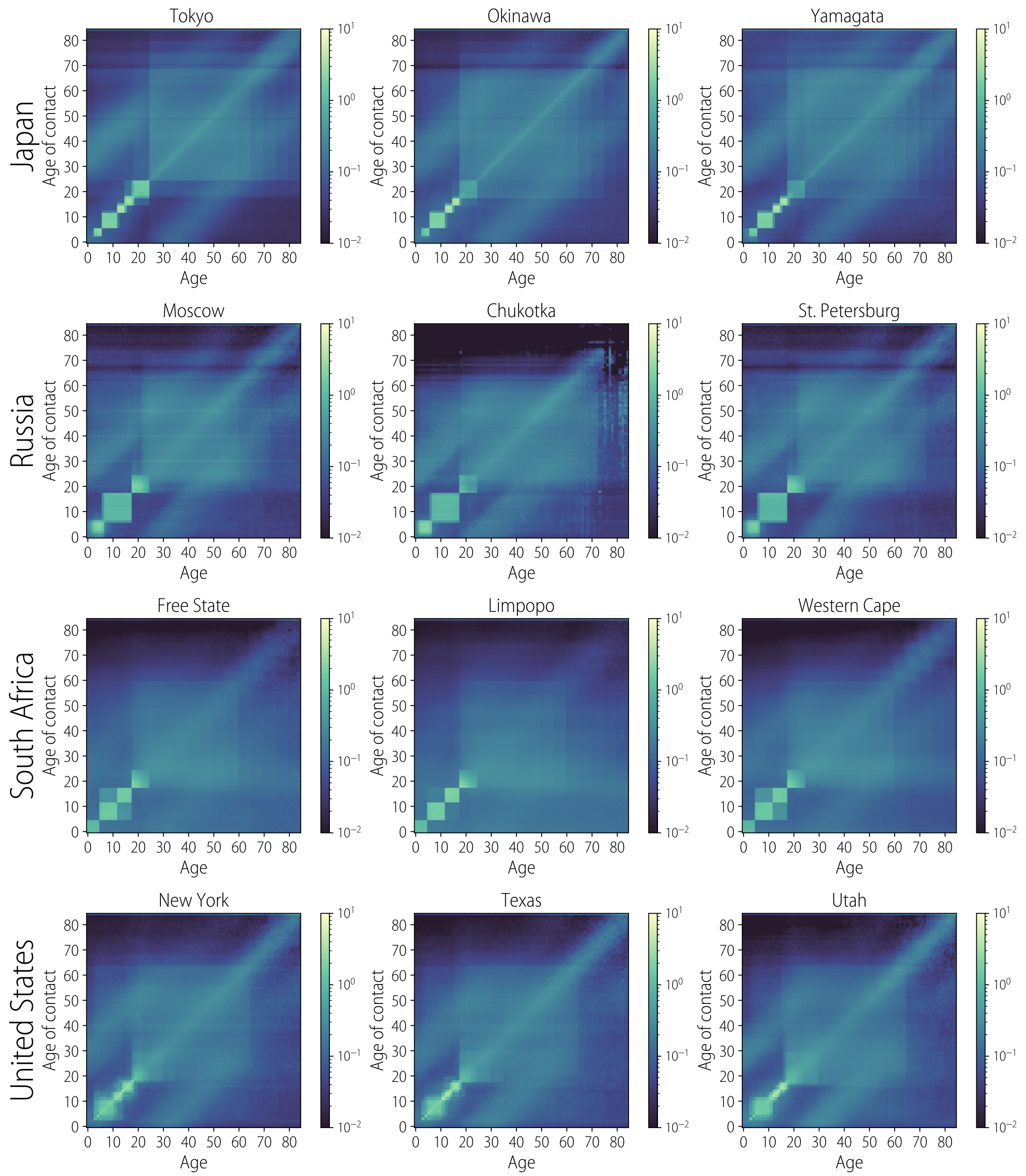

Supplementary Figure 20: **Overall contact matrices.** Each heatmap represents the overall average frequency of adequate contacts for influenza transmission by age in 3 subnational locations Japan (first row), Russia (second row), South Africa (third row), and the United States (last row).

2.4 Assortativity of overall contact matrices

To compare the contact patterns in each location we can also consider a measure of assortativity (i.e., the preference for contact with others of the same age). Assortativity by age of the contact matrix is of special importance in infectious disease modeling as it is classically associated with

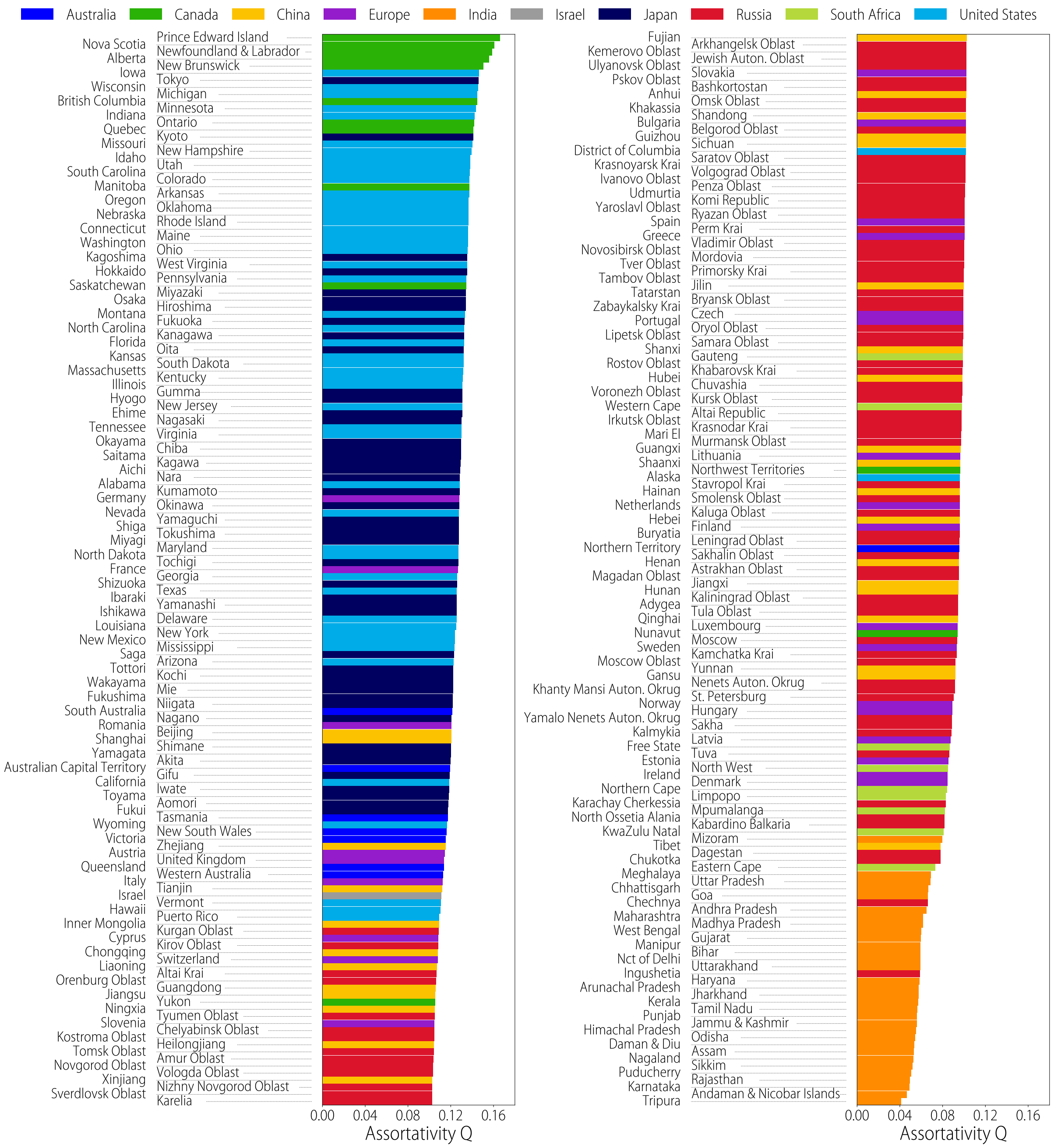

Supplementary Figure 21: Assortativity of the Flu matrix for all locations ordered from lowest to highest. The matrix is aggregated to 5 year age brackets to measure the assortativity as the preference for contact with others in the same age bracket. The last two age brackets representing individuals aged 80-83 years, and 84 years and older, respectively, come as a result of limitations in census age distribution data.

R_0 (i.e., larger assortativity by age entails a larger values of R_0) [28].

Here we aggregate the matrices to 5 year age brackets to measure the assortativity as the preference for contact with others in the same age bracket, with the last two age brackets representing individuals age 80-83 years old and 84 years and older, respectively. As a measure of

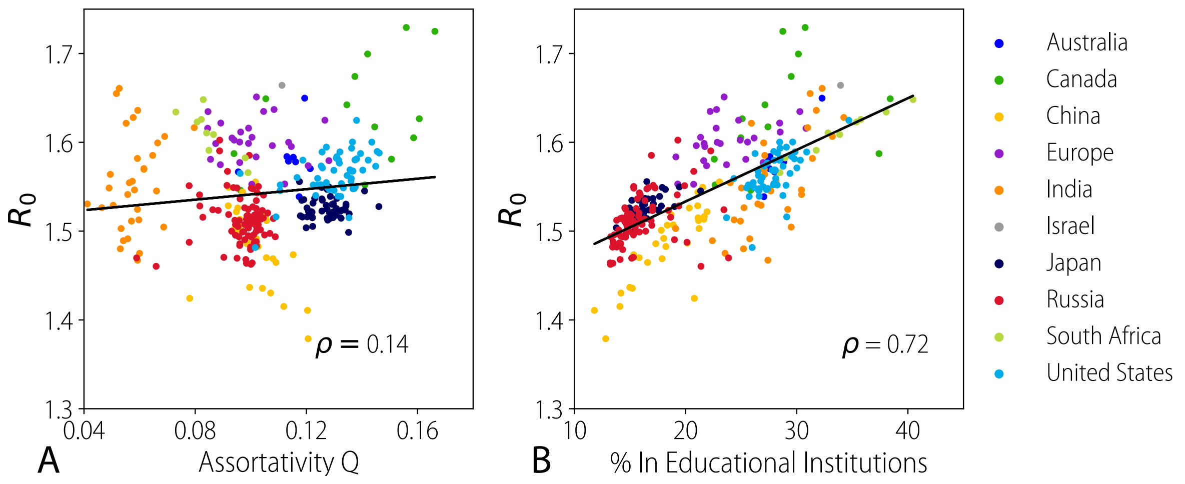

Supplementary Figure 22: **A** Scatter plot of the reproduction number, R_0 and assortativity index, Q. ρ represents the Pearson correlation. The black line represents the linear model best fitting the data. To obtain the plot, matrices are aggregated to 5 year age brackets (except for the last two age groups that are individuals aged 80-83 years, and 84 years and older, respectively). Moreover, we chose the transmission rate such as $R_0 = 1.6$ in the UK; then we kept the same transmission rate in all the other locations as calculated the resulting value of R_0 through the next generation approach. **B** As A, but for reproduction number, R_0 and share of individuals attending educational institutions.

assortativity, we use the assortativty index Q, defined as

$$Q = \frac{Tr(\widetilde{M}) - 1}{n_a - 1} \tag{2}$$

where $\widetilde{M_{ij}} = \frac{M_{ij}}{\sum_j M_{ij}}$, the fraction of contacts that age group i has with age group j, and n_a is the number of age groups to which M is aggregated [29].

Figure 21 shows the assortativity of the overall contact matrix for all locations in our study, where locations are ordered by lowest to highest assortativity. Although a clearcutting pattern does not emerge, our analysis suggests that Western Countries (Canada, Australia, and the US in particular) show a more assortative patterns than less developed locations such as India, and South Africa. Locations characterized by a single school cycle (e.g., for primary and middle school) such as Russia and several Eastern European countries show a low level of assortativity as well.

As expected by the classic theory of mathematical epidemiology [28], we find that the assortativity of the contact matrix is positively correlated with R_0 (Fig. 22A). However, such a correlation is not as strong as it may have been expected: Pearson correlation coefficient 0.14, p-value = 0.015. In fact, the share of population that attends an educational institution is a much better predictor of R_0 than the assortativity (Pearson correlation coefficient 0.72, p-value < 0.0001 - see Fig. 22B). These two results combined show the role of the contact patterns and population demography in determining location specific R_0 values.

2.5 Uncertainty of overall contact matrices

As the data used to create the matrices comes not only from censuses, but also from surveys, a certain degree of uncertainty is present. The uncertainty comes from sampling data either from

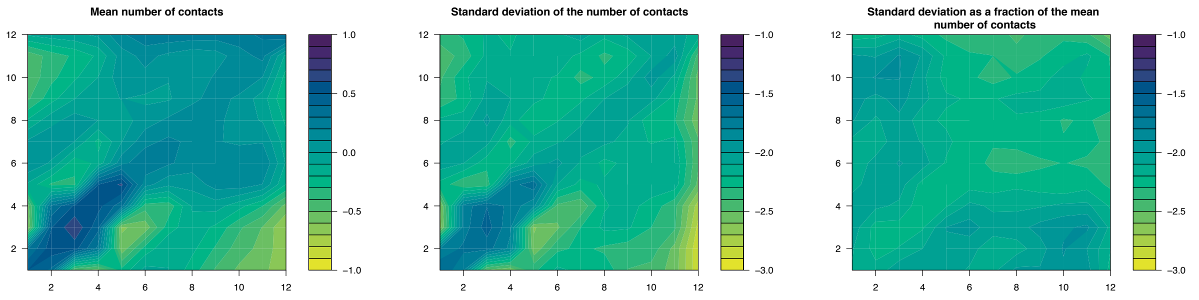

Supplementary Figure 23: **Uncertainty on the overall contact matrix for Tomsk region, Russia.** **A** Each cell value represents the mean (log_{10}) of the number of contacts that an individual of age i has with individuals of age j estimated from 100 realizations of the synthetic population. **B** Same as A, but each cell represents a standard deviation (log_{10}). **C** Same as A, but each cell represents a ratio of standard deviation over the mean of the number of contacts (log_{10}).

conditional (multinomial) distributions created from survey data (e.g. China, India, Russia) or from sampling directly from the survey conditional on a set of census-based characteristics (e.g. partially US households). However, it is important to note, that the synthetic populations created to produce the contact matrices has the same or comparable size as the real population of each specific location. This allowed us to sample the survey-based distributions exhaustively. Moreover, we weighted our sampling according to distributions available from the census data for each particular subpopulation. As a result, the level of uncertainty compared with the differences in the number of contacts between age groups is negligible.

To illustrate the total level of uncertainty associated with the creation of the overall matrices,

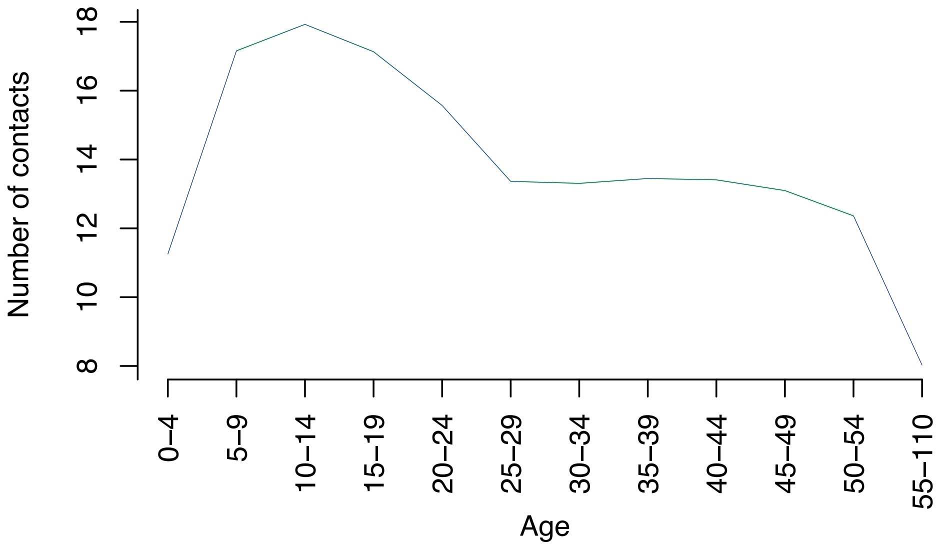

Supplementary Figure 24: **Average number of contacts by age group in Tomsk region, Russia.** The solid line shows the mean of the distribution of the average number of contacts as resulting from 100 realization of the synthetic population. The light green area represent quantiles 0.025 and 0.975 of this distribution. Note that the green area is barely visible unless the figure is considerably magnified.

we have generated 100 realizations of the same synthetic population for one of the locations and calculated the overall matrix for each realization. As an example, we used Tomsk Oblast, Russia as for this region we have direct access to all original diaries collected in the diary-based contact survey [27]. The higher level of uncertainty was introduced to the household matrices (see Fig. 25) due to the higher number of sampled characteristics. However, the standard deviation of the number of contacts between age groups in such an exercise is extremely small and varies between 10^{-3} and 10^{-1} (the matrices of the mean number of contacts from the overall matrix, the standard deviation and their relation are presented in Fig. 23). When looking at the mean number of contacts that individuals of each age group has, the 95% of the distributions is negligible (and almost invisible in the visualization given in Fig. 24))

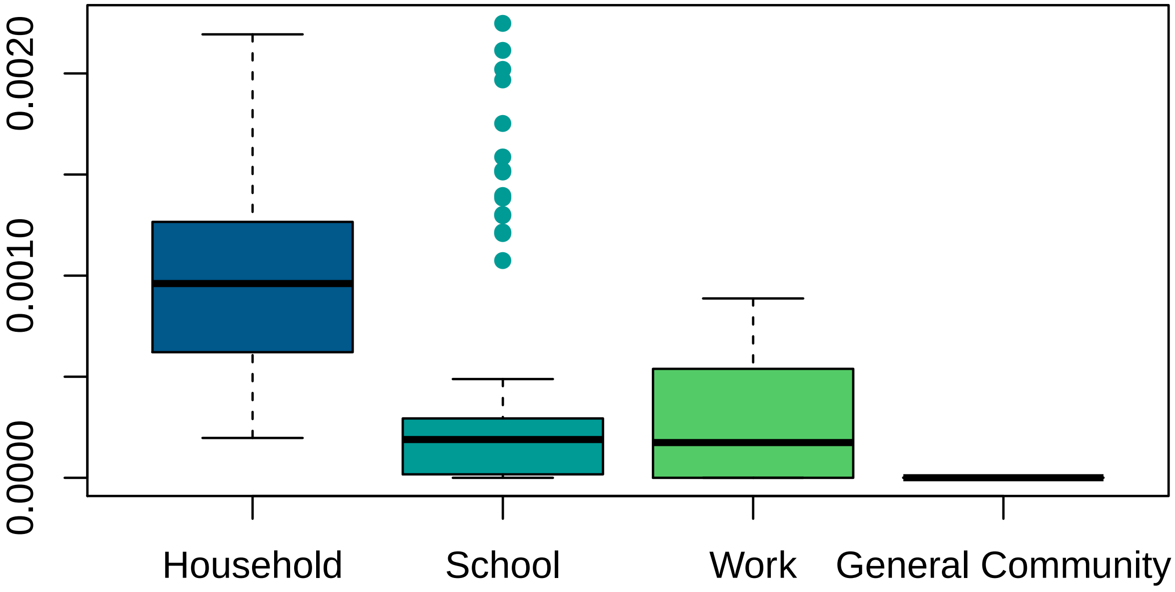

Supplementary Figure 25: **Uncertainty of the number of contacts by age by setting for Tomsk region, Russia.** The boxplot shows the distribution of the standard deviation of the synthetic setting-specific contact matrices based on 100 replications of the 207,006 households and approximately 515 thousand synthetic individuals. In the boxplot, the middle line corresponds to the median, the lower and upper hinges correspond to the first and third quartiles, the upper whisker extends from the hinge to the largest value no further than 1.5IQR from the hinge (where IQR is the inter-quartile range) and the lower whisker extends from the hinge to the smallest value at most 1.5IQR of the hinge, while data beyond the end of the whiskers are outlying points that are plotted individually.

Results obtained so far considered a population with comparable size of the actual population. Now we want to explore whether, if considering a population of a size comparable to contact survey studies, we obtain a similar variability between survey-based contact matrices and the synthetic ones.

We continue to focus on Tomsk region of Russia. To define the uncertainty in the survey-based contact matrix, we used a standard bootstrap procedure [30]. Specifically, we sampled 503 diaries (the actual size of the original survey [27]) proportional to the number of individual by age group according to the census data and estimate the resulting contact matrix. We then repeat this procedure 100 times.

For the synthetic contact matrix, we cannot generate a synthetic population as little as 500 individuals as this would destroy the characteristics of the actual population (e.g., school size, workplace size). Therefore, we sampled 503 (synthetic) individuals proportional to the number of individual by age group according to the census data and record all their characteristics (e.g., the age of all its synthetic household members, of its synthetic work colleagues). Then, we repeat this sampling 1,000 times.

We performed a bootstrap sampling of the synthetic population of Tomsk region from the population age structure (Census data) to select the number of individuals equal to the number

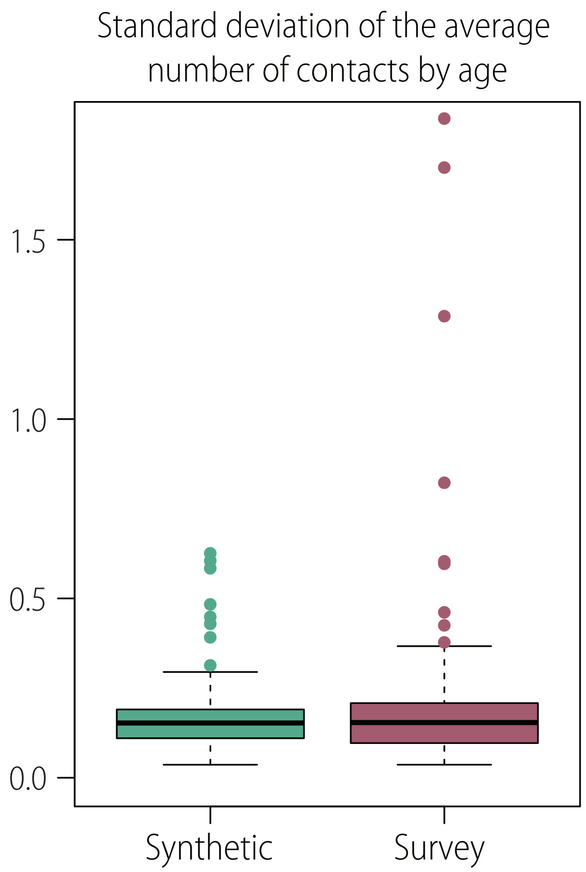

Supplementary Figure 26: **Uncertainty of the number of contacts by age for Tomsk region, Russia.** The boxplot shows the distribution of the standard deviation of the synthetic and survey-based overall contact matrices. The contact matrices are based on 1,000 replications of the bootstrap sampling of 503 individuals both for the synthetic and survey-based contact matrices. In the boxplot, the middle line corresponds to the median, the lower and upper hinges correspond to the first and third quartiles, the upper whisker extends from the hinge to the largest value no further than 1.5IQR from the hinge (where IQR is the inter-quartile range) and the lower whisker extends from the hinge to the smallest value at most 1.5IQR of the hinge, while data beyond the end of the whiskers are outlying points that are plotted individually.

of participants of the survey. This procedure resulted in 1,000 realizations of the subsample of the synthetic population, each consisting of 503 individuals. For each realization we then calculated the setting-specific matrices and estimated the overall matrix by using the procedure described in Section 2.2 and the weights w_K estimated therein.

Fig. 26 presents the distribution of the standard deviation (σ_{ijk}) of the average number of contacts that an individual of age group i has with individuals of age group j, where $k \in \{0, 1000\}$ is the number of the bootstrap realization. The resulting distribution of the variability shows high similarity. Moreover, we found comparable results also when analyzing the uncertainty between survey and synthetic estimations for each cell of the contact matrix (Fig. 27).

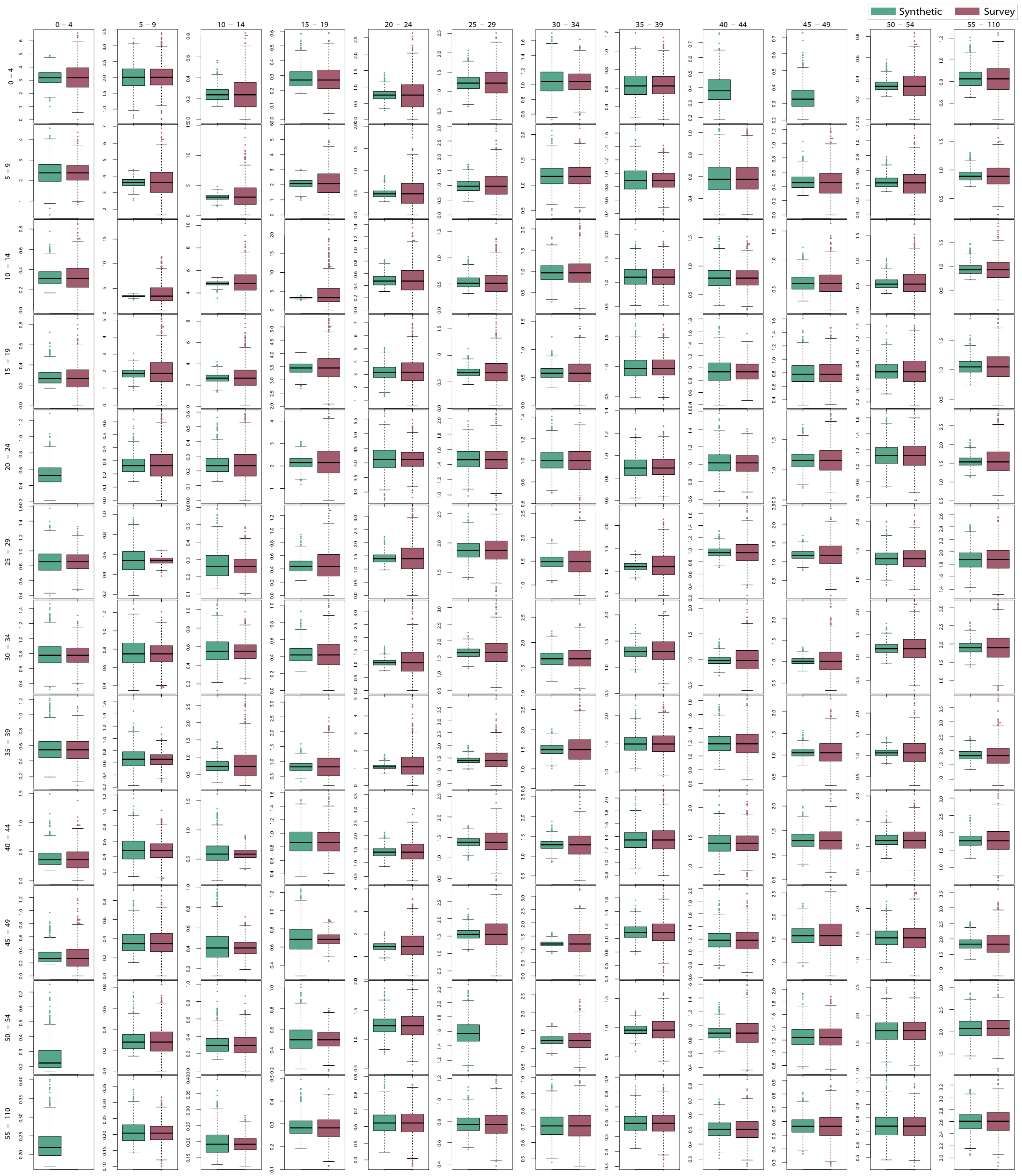

Synthetic
Survey
0 – 4
5 – 9
10 – 14
15 – 19
20 – 24
25 – 29
30 – 34
35 – 39
40 – 44
45 – 49
50 – 54
55 – 110

Supplementary Figure 27 *(previous page)*: **Uncertainty of the number of contacts between age groups for Tomsk region, Russia.** Each panel of the figure shows the boxplot (percentile 2.5, 25, 50, 75, and 97.5) of the distribution of the average number of contacts that of individuals of age i have with individuals of age j. The distributions are obtained by considering 1,000 bootstrap sampling of 503 study participants for the survey-based contact matrix and of 503 synthetic individuals for the synthetic contact matrix. In order to focus on the uncertainty of estimates, rather than comparing medians for each cell, we renormalized the survey bootstrap estimates for each matrix element so that their median match the median of the synthetic bootstrap estimates. The resulting total mean number of contacts is 5,004. Where the boxplot for the survey-based contact matrix is not shown, it means that 0 contacts were recorded in the survey among those age groups. In the boxplot, the middle line corresponds to the median, the lower and upper hinges correspond to the first and third quartiles, the upper whisker extends from the hinge to the largest value no further than 1.5IQR from the hinge (where IQR is the inter-quartile range) and the lower whisker extends from the hinge to the smallest value at most 1.5IQR of the hinge, while data beyond the end of the whiskers are outlying points that are plotted individually.

3 Modeling the spread of the 2009 H1N1 pandemic influenza

In the main text, we presented a general age-structured SIR model. However, one of the main feature of the 2009 H1N1 influenza pandemic was a larger susceptibility to infection of young individuals than adults and the elderly [31, 32, 33]. We introduce the age-specific susceptibility to infection in the age-structured as follows.

First, we remind the notation used for the age-structured SIR model:

$$\begin{aligned} \dot{S}_i &= -\lambda_i S_i \\ \dot{I}_i &= \lambda_i S_i - \gamma I_i \\ \dot{R}_i &= \gamma I_i \end{aligned} \tag{3}$$

where S_i is the number of susceptible individuals of age i, I_i is the number of infected individuals of age i, R_i is the number of recovered or removed individuals of age i; γ^{-1} is the infectious periods; and λ_i represents the force of infection to which an individual of age i is exposed to other infected individuals.

Second, we modify λ_i to introduce the age-specific susceptibility to infection as follows:

$$\lambda_i = \beta \chi_i \sum_j M_{ij} \frac{I_j}{N_j}$$

where β is the transmissibility of the infection, N_i is the total number of individuals of age i, χ_i is the age-specific susceptibility to infection of individuals of age i, and M_{ij} measures the average number of contacts for an individual of age i with all of their contacts of age j.

We assume a simple two-step function for the age-specific susceptibility to infection:

$$\chi_i = \begin{cases} 1 \text{ for } i < 18 \\ \chi \text{ for } i \geq 18, \end{cases}$$

where the specific value χ is obtained by calibrating the model on the seroprevalence data from the 2009 H1N1 pandemic (see Sec. 3.2).

3.1 Calculation of the reproduction number

The basic reproduction number R_0, representing number of cases generated by a typical index case in a fully susceptible population. This definition can be extended to the effective reproduction number R_{Eff} that represents the number of cases generated by a typical index case in a partially immune population [34].

We use the next-generation matrix approach [35] to calculate R_0 for the age-structured model defined by Eq. (3).

$$R_0 = \frac{\beta}{\gamma} \rho(Q) \tag{4}$$

where $\rho(Q)$ is the spectral radius of matrix Q, whose element are given by $Q_{ij} = \chi_i M_{ji}$. Note that in the case of the generic age-structured model, Equation (4) simply becomes $R_0 = \frac{\beta}{\gamma} \rho(M)$, as $\chi_i = 1$ for all i.

3.2 Calibration of the infection transmission model

The infection transmission model regulated by Eq. (3) has two "unknown" location-specific parameters, namely the transmission rate β and the susceptibility to infection by age χ_i. The set of parameters to be estimated for each location is thus $\Theta = (\beta, \chi)$. The posterior distribution of Θ

is estimated through Markov chain Monte Carlo (MCMC) sampling of the binomial likelihoods ($\mathcal{L}$) of the age-specific prevalence of H1N1 antibodies observed in the five locations for which serological survey in the general population were conducted before and after the end of the 2009 H1N1 influenza pandemic, namely Israel [36], Italy [37], Japan [38, 39], the United Kingdom [40], and the United States [41].

The likelihood function is defined as

$$\mathcal{L}(n, r|\Theta) = \prod_{g \in G} \frac{n_g!}{r_g!(n_g - r_g)!} (\alpha_g(\Theta))^{r_g} (1 - \alpha_g(\Theta))^{n_g - r_g}$$

where G is the set of age groups considered in the serosurvey data; n_g is the number of individuals in the g-th age group in the dataset; r_g is the number of seropositive individuals in the g-th age group in the dataset; $\alpha_g(\Theta)$ is the fraction of removed individuals at the end of the pandemic in the g-th age group as resulting from model simulation performed by using parameter set Θ.

The age-specific fraction of immune (removed) individuals prior the pandemic is set in the model according to the location-specific pre-pandemic seropositive rates [36, 37, 38, 39, 40, 41], and simulations are initialized with a fraction of 10^{-5} infectious individuals in the population of each location. The posterior distribution of Θ is determined using random-walk Metropolis-Hastings sampling [42]. We performed 12,000 simulations and considered a burn-in period of 2,000 iterations. We assume no a priori knowledge on model parameters (i.e., flat prior distributions). Convergence was checked by considering different starting points and by visual inspection. The trace plots of the estimated parameters (β and χ_i) as well as the values of the likelihood, of R_{Eff}, and of the final attack rate are shown in Fig. 28, 29, 30, 31, and 32.

The posterior distributions of the effective reproductive number and of the susceptibility to infection of adults are reported in Fig. 33A and B. The estimated R_{Eff} is relatively stable between locations, with averages ranging form 1.31 to 1.51 (see Tab. 5). The estimated values

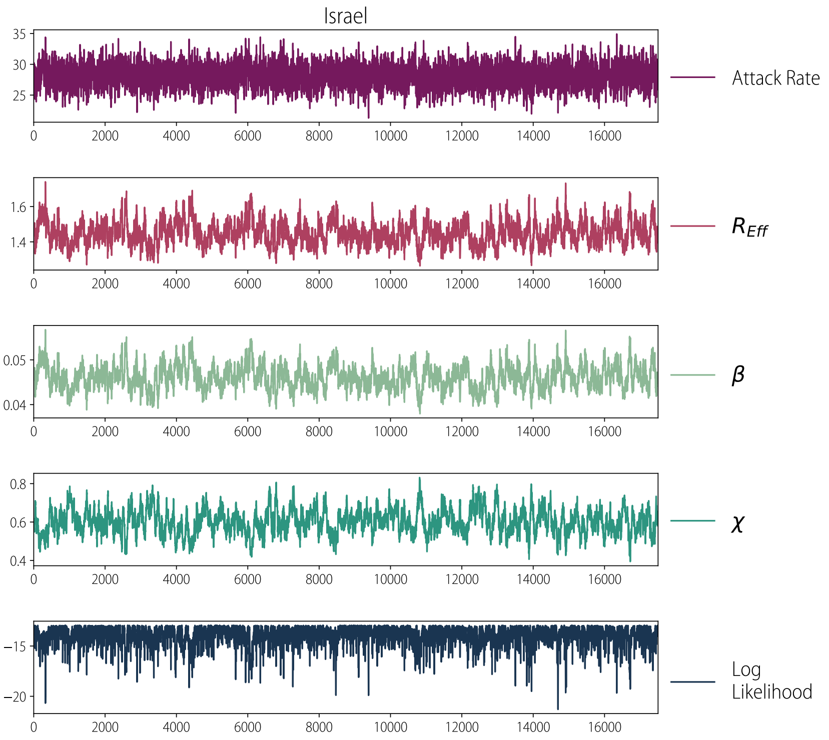

Supplementary Figure 28: Trace plots for Israel.

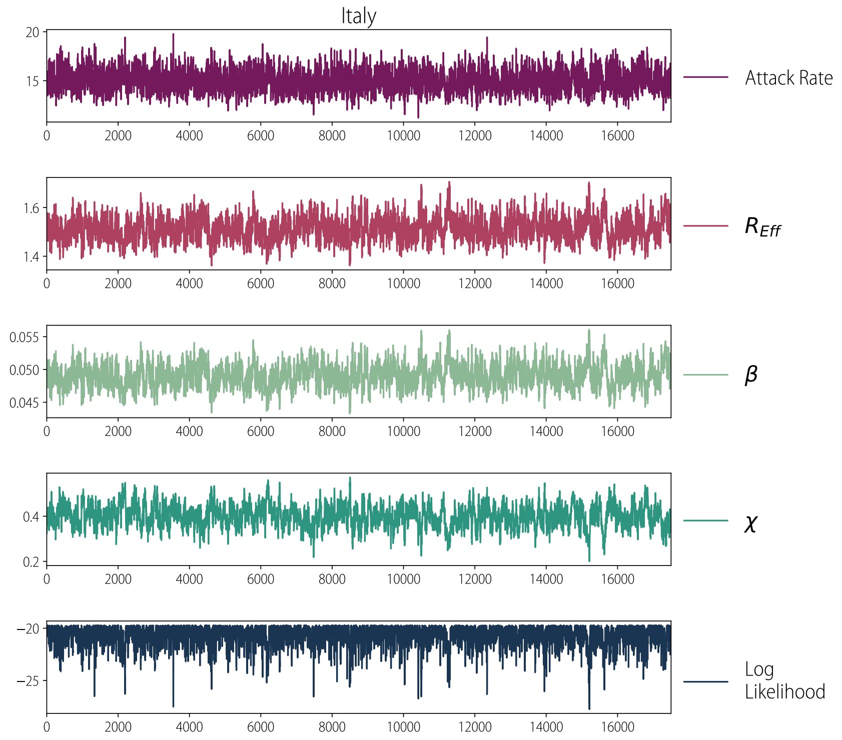

Supplementary Figure 29: Trace plots for Italy.

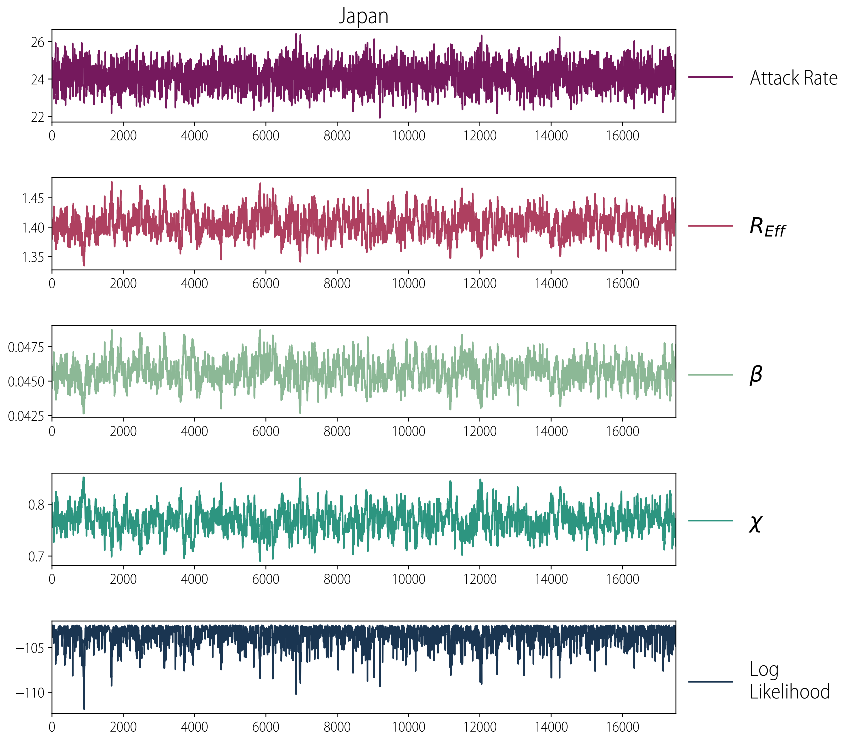

Supplementary Figure 30: Trace plots for Japan.

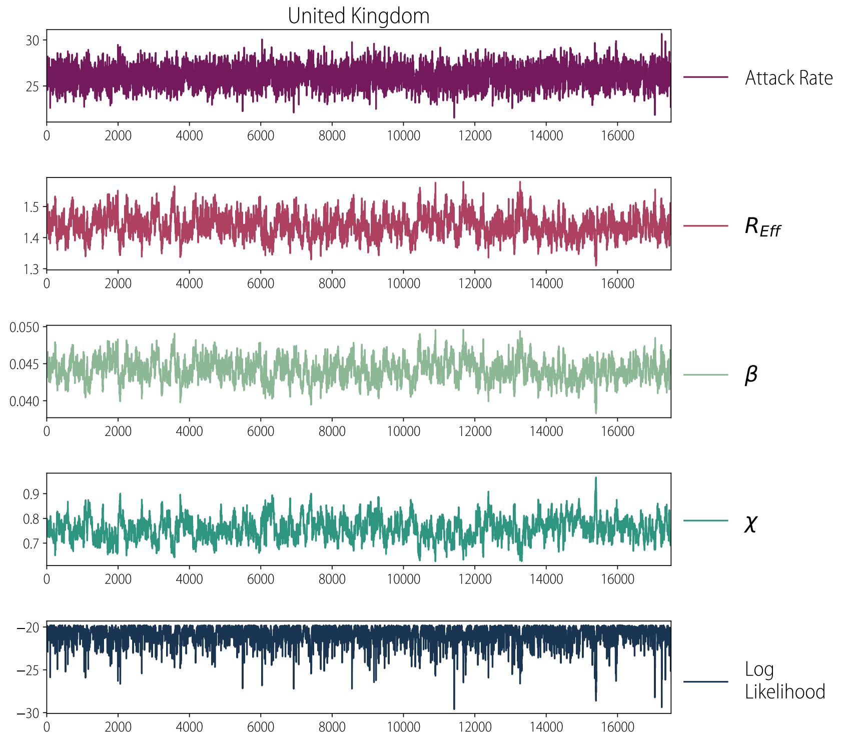

Supplementary Figure 31: Trace plots for the UK.

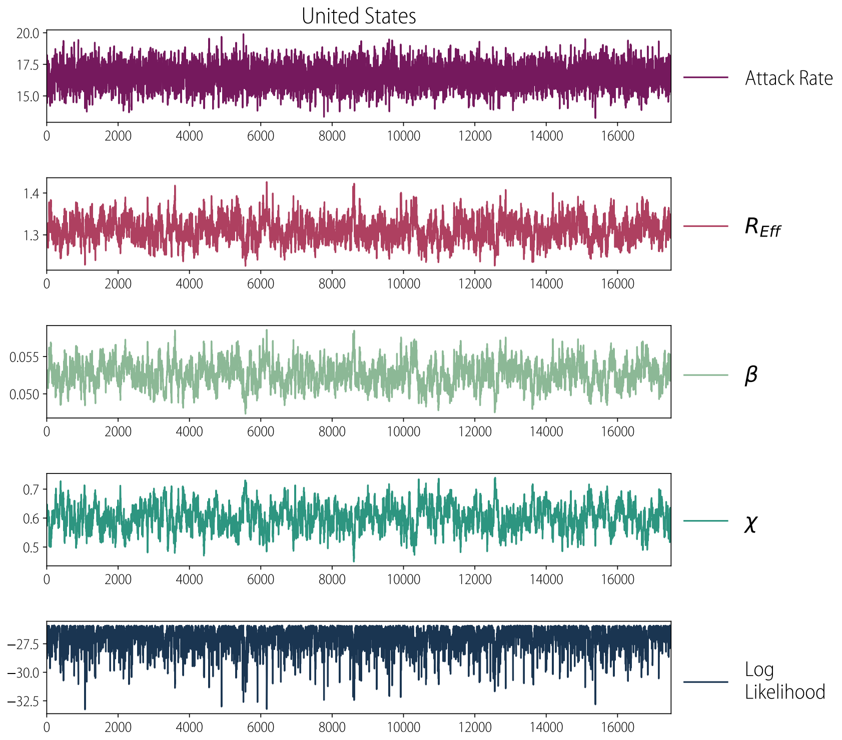

Supplementary Figure 32: Trace plots for the US.

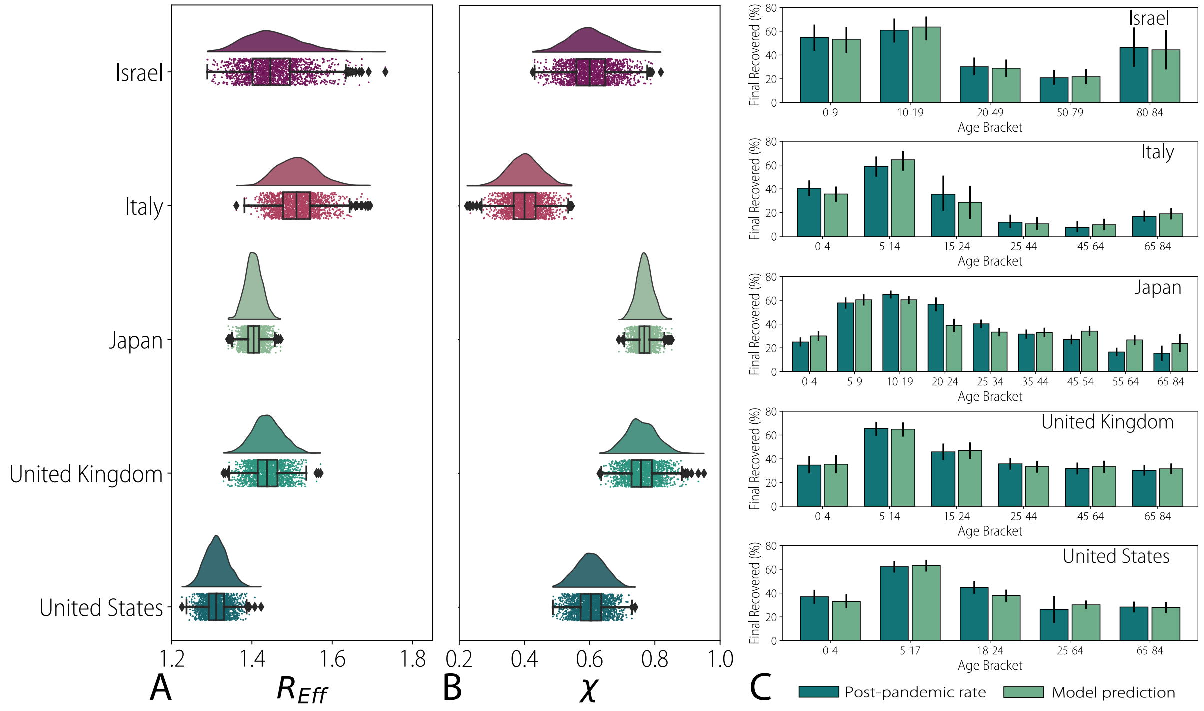

Supplementary Figure 33: **Indirect validation of the inferred age-mixing patterns.** **A** Posterior distributions of the effective reproduction number (R_{Eff}) in the five locations with suitable seroprevalence data. The distribution of results is from 17,500 simulations for each location. The boxplots show the percentile 2.5, 25, 50, 75, and 97.5 of the distribution.**B** Posterior distributions for the susceptibility to infection of adults (individuals aged 18 years or more) with respect to children (χ). The distribution of results is from 17,500 simulations for each location. The boxplots show the percentile 2.5, 25, 50, 75, and 97.5 of the distribution.**C** Comparison between the post-pandemic fraction of seropositve individuals by age as observed in the data and the posterior fraction of removed individuals by age as estimated by the model for the five analyzed locations. The confidence interval shown on the top of each histogram bar is the 95% confidence interval.

of R_{Eff} and of the susceptibility to infection of adults with respect to children are in agreement with previous studies [32, 33, 37, 43, 44, 45, 46].

The age-specific seroprevalence rates by age as obtained by simulating the calibrated model compare well with the observed data (Fig. 33C), suggesting that the synthetic contact matrices

Country	R_{Eff} (95%CI)	χ (95%CI)
Israel	1.45 (1.33–1.59)	0.60 (0.47–0.74)
Italy	1.51 (1.43–1.62)	0.40 (0.30–0.50)
Japan	1.40 (1.37–1.46)	0.77 (0.72–0.82)
UK	1.44 (1.37–1.52)	0.76 (0.68–0.85)
USA	1.31 (1.26–1.37)	0.60 (0.52–0.69)

Supplementary Table 5: Data sources for each country and the country code.

developed in this study are able to capture the age-related infection patterns of the 2009 H1N1 influenza pandemic. In all locations, the seroprevalence data indicate that school-aged individuals are the most infected, however the age related patterns are otherwise different reflecting the different populations and their respective behaviors. For example, relative to Italy, adults in the United Kingdom age 25 years and older show higher post-pandemic rates, while the post-pandemic rates for school-aged individuals show less than 5% difference between the two countries.

3.3 Alternative method for the calculation of the reproduction number

In Sec. 3.1, we introduced the next-generation matrix approach to calculate the reproduction number. However, alternative methods can be found in the literature. In particular, R_0 can be estimated from the initial exponential growth and the distribution of the generation time [47]. Specifically, in the case of a SIR model, the following equation can be used:

$$R_0 = 1 + r\tau_g \tag{5}$$

where r is the initial exponential growth of the epidemic and τ_g is the generation time or serial interval (which, in the case of a SIR model, corresponds to the average duration of the infectious period) [47].

Figure 34 shows that in our simulations the values of R_0 found by applying the two different methods are well in agreement. Therefore, without loss of generality, in all the analyses presented in this paper we always use the next-generation matrix approach to calculate the reproduction number.

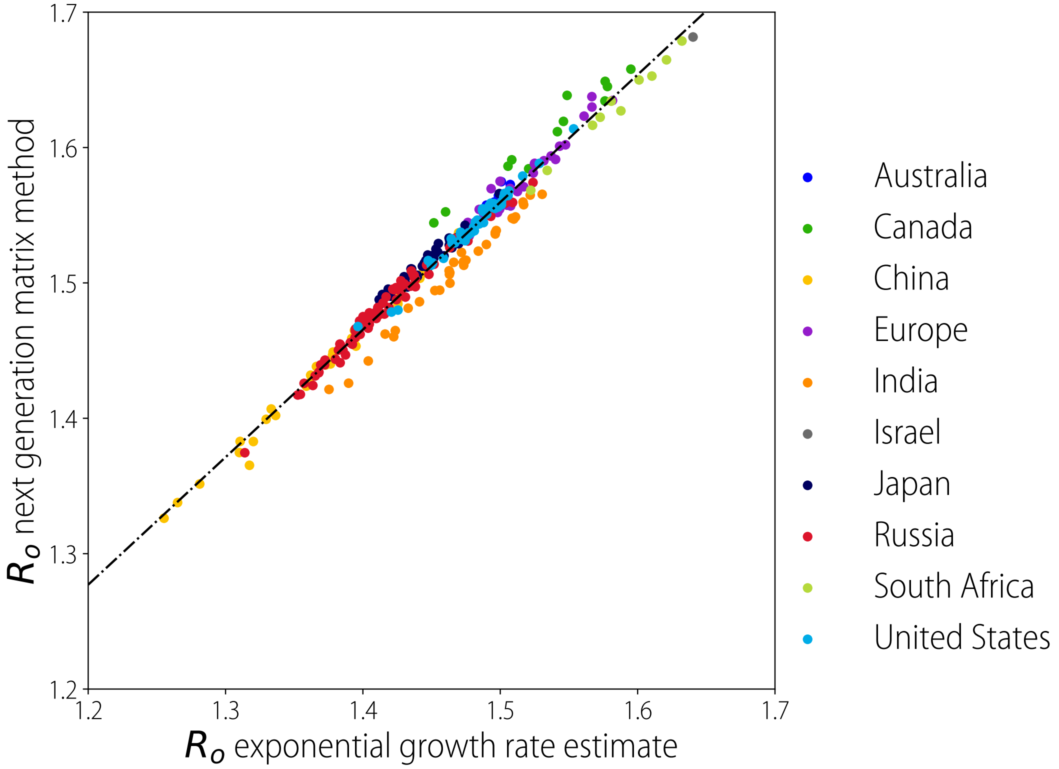

Supplementary Figure 34: Comparison of the reproduction number as obtained from the next-generation matrix approach and as derived from the analysis of the exponential growth of the simulated epidemics.

3.4 Simulations of epidemics resembling the 2009 H1N1 pandemic influenza

We conduct the same analysis reported in the main text to estimate the impact of population mixing patterns and demography on the spread of an epidemic resembling the 2009 H1N1 pandemic influenza. In particular, we calculate the average transmission rate and susceptibility to infection of adults withe respect to children in the five available countries. Then, we use these average values in all locations to project the dynamics of an hypothetical epidemic in each location.

We find a large variability across the different locations both in terms of R_0 and final attack rate (Fig. 35A). Although there is an evident pattern of increase in the attack rate for larger values of R_0, it is also clear that the reproduction number is not the only determinant of such a trend. In fact, we estimate that the epidemic attack rate is strongly (negative) correlated with the average age of the population (Fig. 35B) and strongly (positive) correlated with the share of the population attending educational institutions (Fig. 35C). Such patterns are more evident than for the analysis presented in the main text as the inclusion of an age-specific susceptibility to infection enhance the impact of the socio-demographic characteristics of the population on the epidemic spread.

Figures 36 and 37 show the attack rates by age for a sample of the locations in our study. In all locations school aged individuals exhibit the highest attack rates by age, lending support to the school setting as an important environment for transmission of an epidemic sharing features similar to those observed in the 2009 H1N1 pandemic. However, the analysis also highlights between- and within- country differences in the attack rates by age both in absolute terms and in the pattern by age. Figures 38 and 39 show the in all locations, the large prevalence of infection in school-age individuals is ascribable to contacts in the school setting where individuals have a high frequency of contacts.

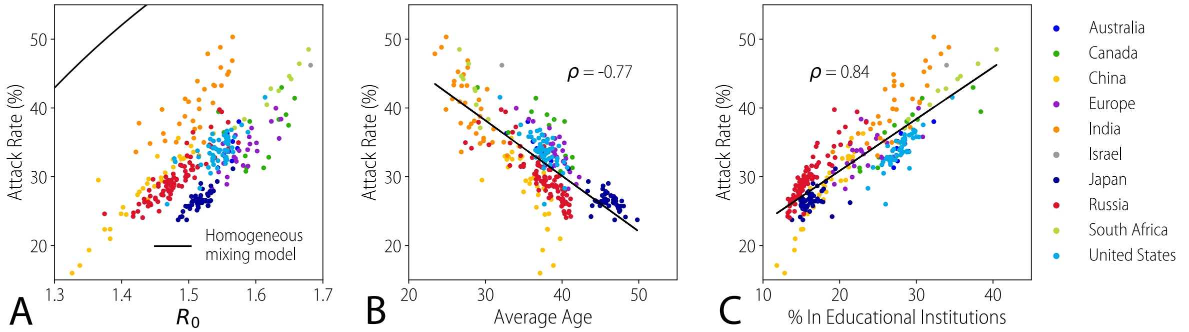

Supplementary Figure 35: **A** Scatter plot of the attack rate and the reproduction number R_0 from an age-structured SIR model using the contact matrix for each subnational location. The parameters β and χ are fixed in all locations are equal to the average values computed over the posterior distributions obtained for the five locations included in the analysis of 2009 H1N1 influenza pandemic seroprevalence data. The black line shows the results of the classic random mixing SIR model (no age groups) **B** Scatter plot of attack rates and average age in each location. The black line represents the best fitting linear model demonstrating a negative linear correlation between attack rates and average age of the population. **C**) Scatter plot of attack rates and percentage of the population attending educational institutions in each location. The black line represents the best fitting linear model.

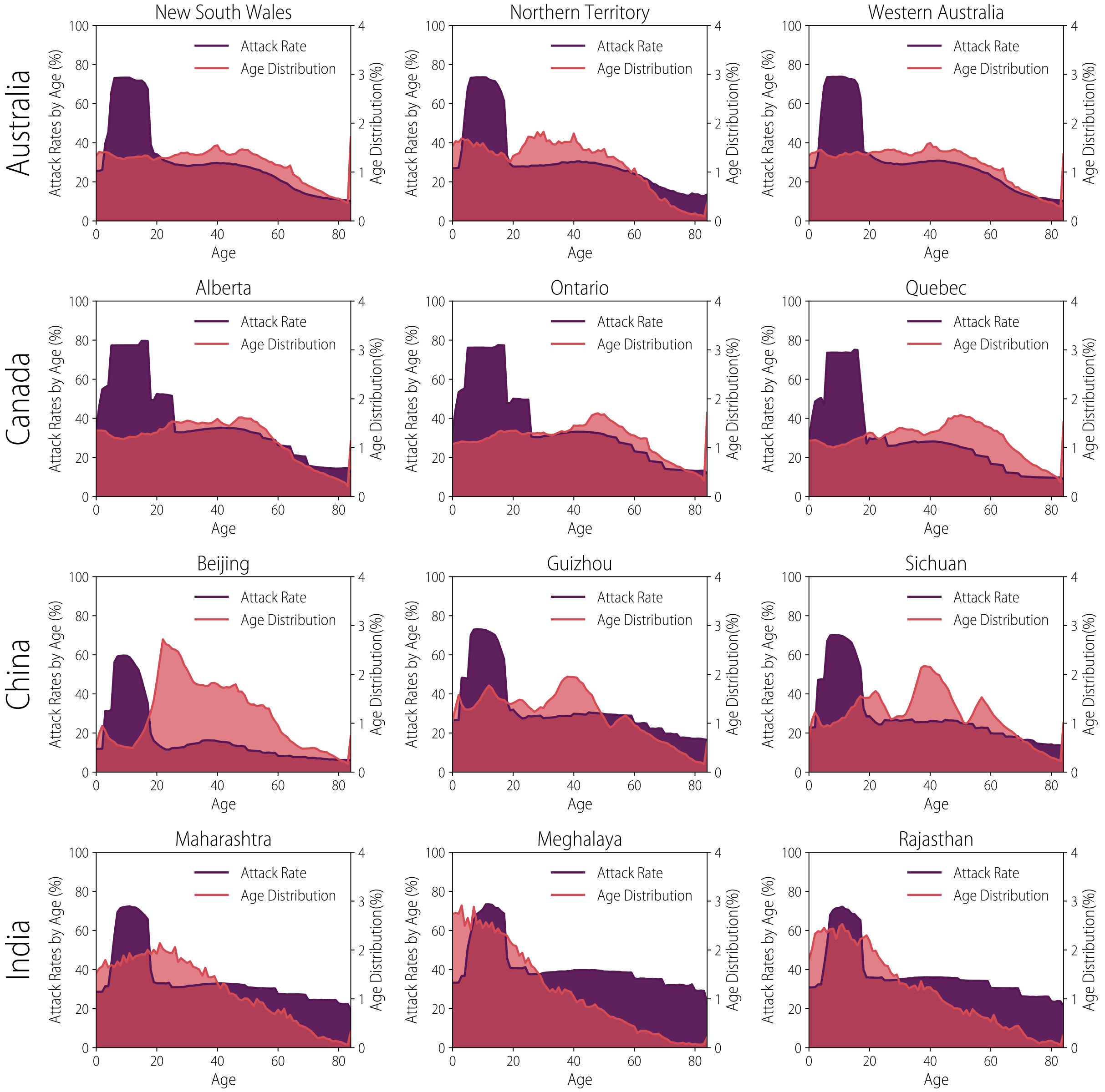

Supplementary Figure 36: Estimated attack rates by age for a subset of locations. The parameters β and χ are fixed in all locations are equal to the average values computed over the posterior distributions obtained for the five locations included in the analysis of 2009 H1N1 influenza pandemic seroprevalence data. As for comparison, the age structure of the population in each specific location is shown as well.

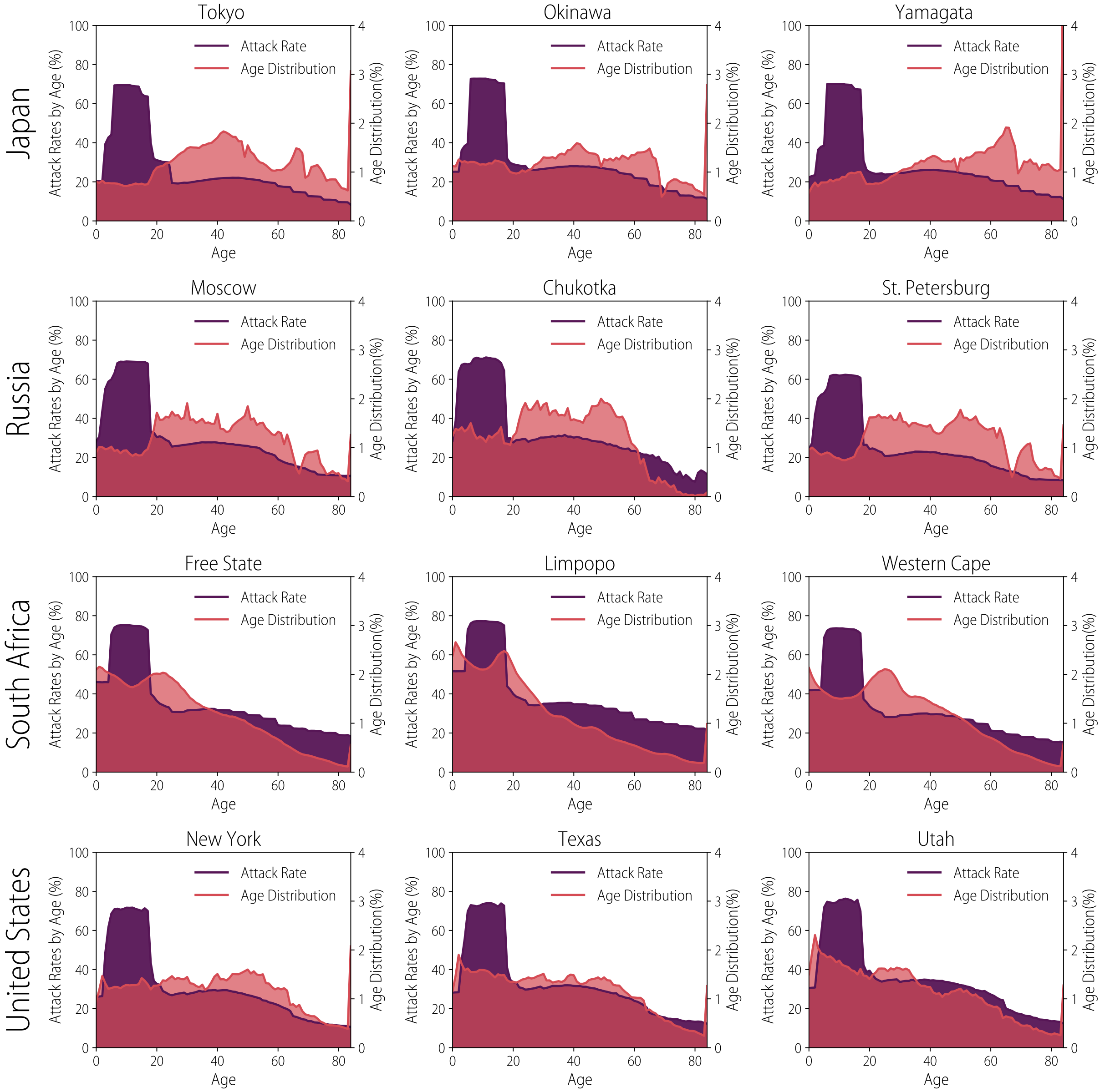

Supplementary Figure 37: Estimated attack rates by age for a subset of locations. The parameters β and χ are fixed in all locations are equal to the average values computed over the posterior distributions obtained for the five locations included in the analysis of 2009 H1N1 influenza pandemic seroprevalence data. As for comparison, the age structure of the population in each specific location is shown as well.

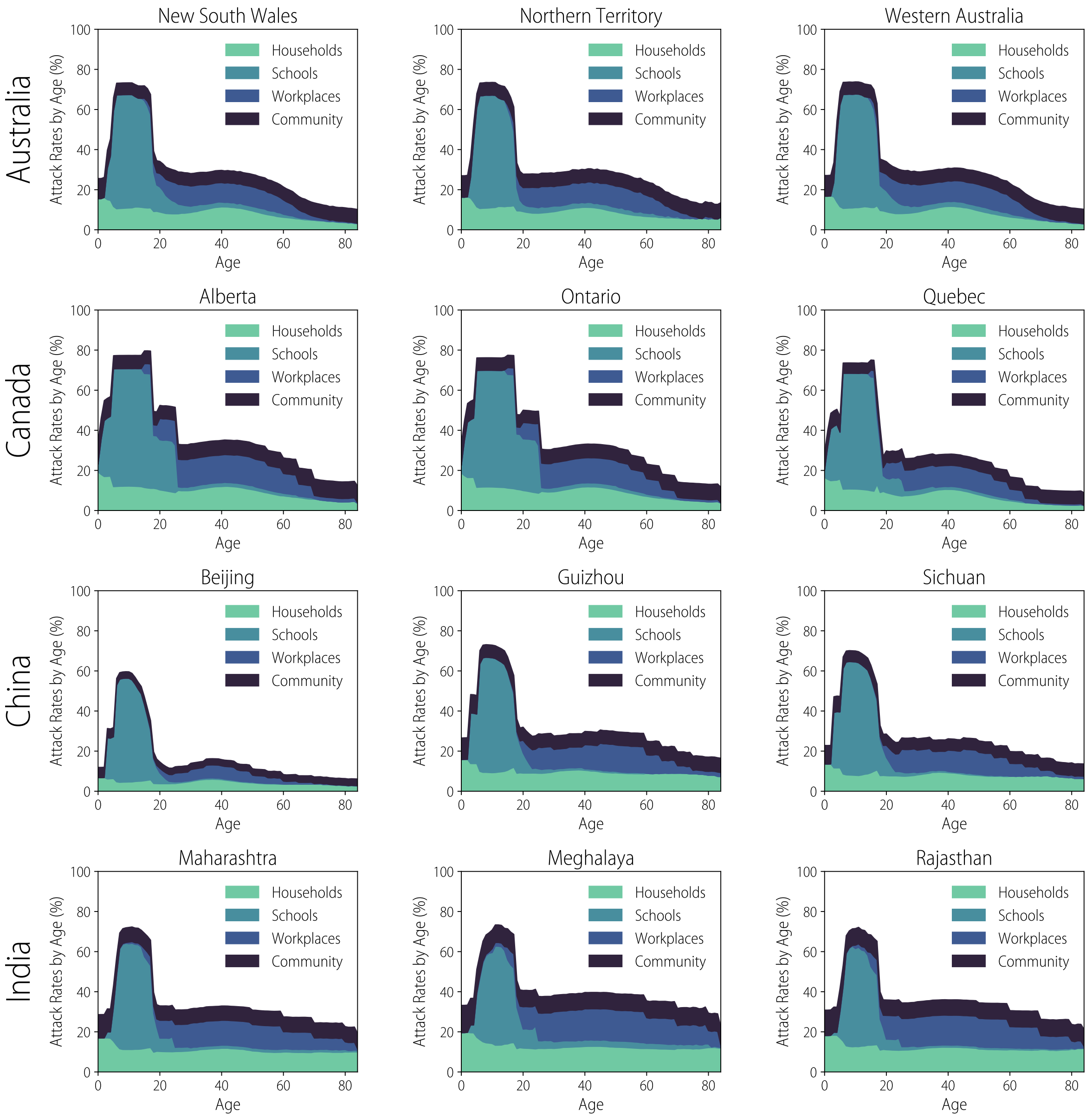

Supplementary Figure 38: Estimated attack rate by age disaggregated by the social setting where the infection took place for a subset of locations. The parameters β and χ are fixed in all locations are equal to the average values computed over the posterior distributions obtained for the five locations included in the analysis of 2009 H1N1 influenza pandemic seroprevalence data.

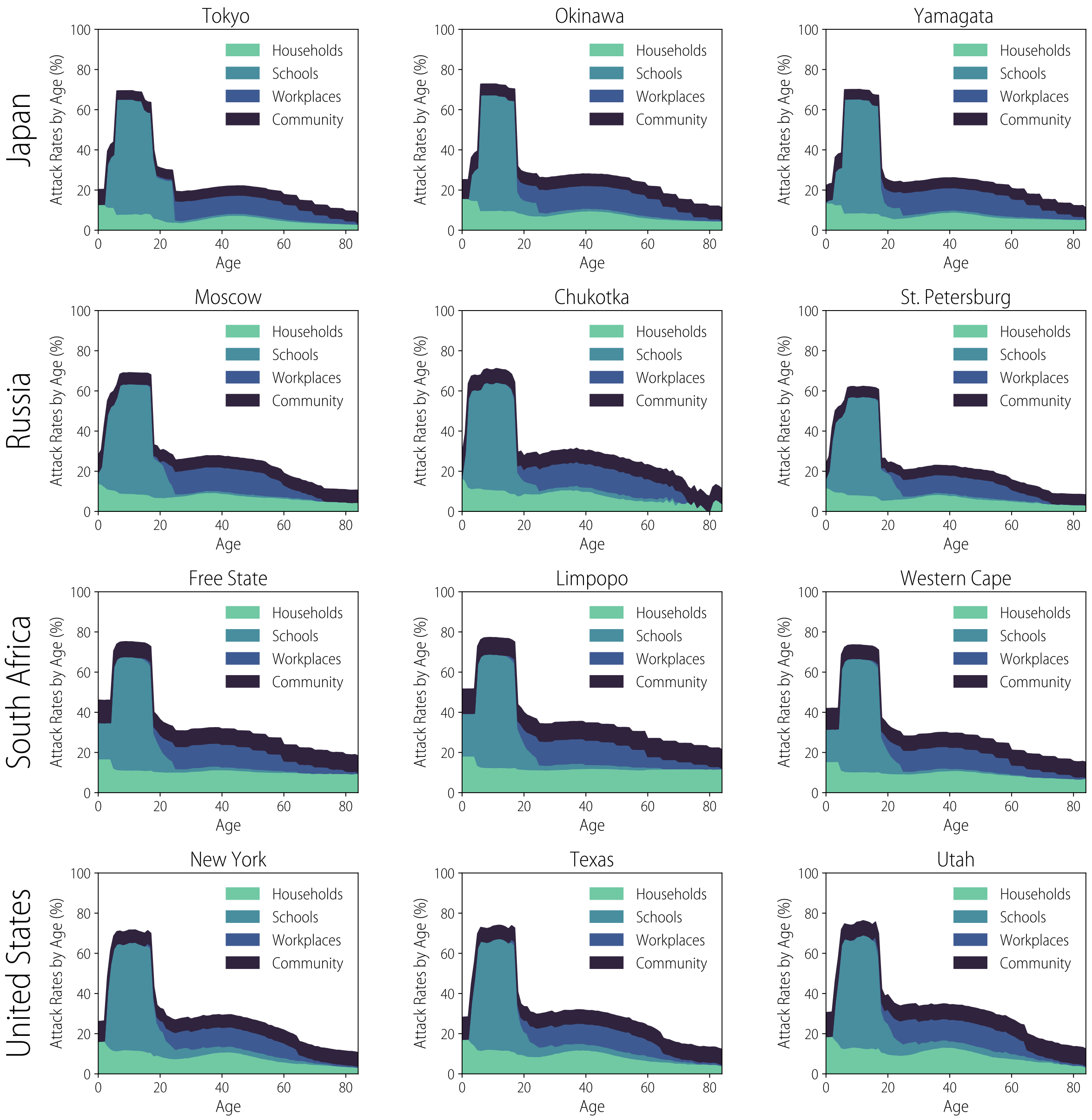

Supplementary Figure 39: Estimated attack rate by age disaggregated by the social setting where the infection took place for a subset of locations. The parameters β and χ are fixed in all locations are equal to the average values computed over the posterior distributions obtained for the five locations included in the analysis of 2009 H1N1 influenza pandemic seroprevalence data.

Figures 40 and 41 show a heatmap of the temporal profiles of the simulated epidemic, i.e. the rate per capita (by age) of new infections as they occur over the course of the epidemic for a sample of locations in our study. In all locations school aged individuals are infected on average earlier than the rest of the population (about 1-2 weeks) and show a larger peak day incidence with respect to all the other age groups. Overall, locations with younger populations show higher per capita incidence rates and quicker epidemics.

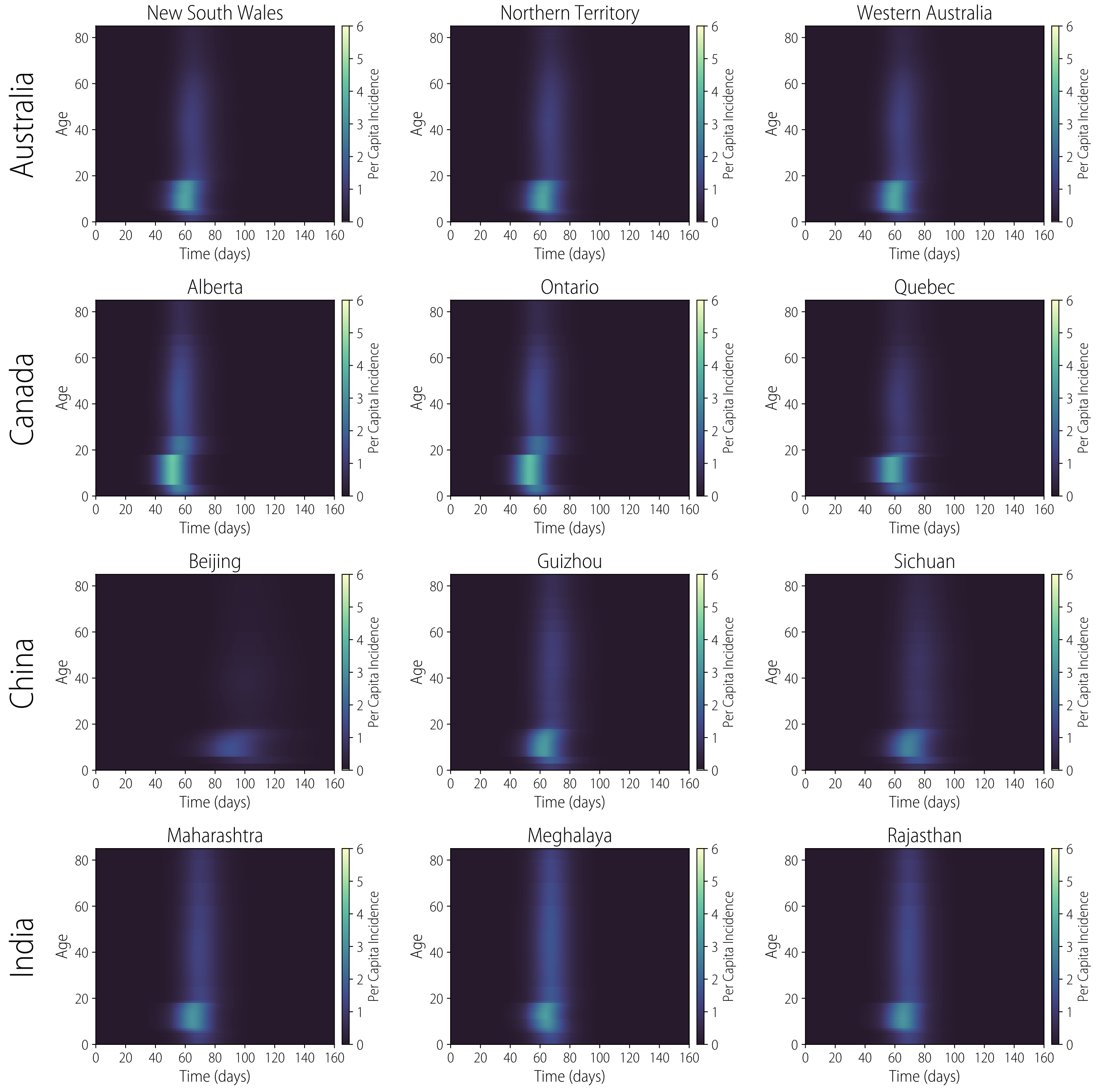

Supplementary Figure 40: Heatmap of the incidence of new infection by age over time for a subset of locations. Given the different population sizes among the locations, in order to allow the comparison of the temporal dynamics of the epidemic, the same initial prevalence of infectious individuals is used in all locations. The parameters β and χ are fixed in all locations are equal to the average values computed over the posterior distributions obtained for the five locations included in the analysis of 2009 H1N1 influenza pandemic seroprevalence data.

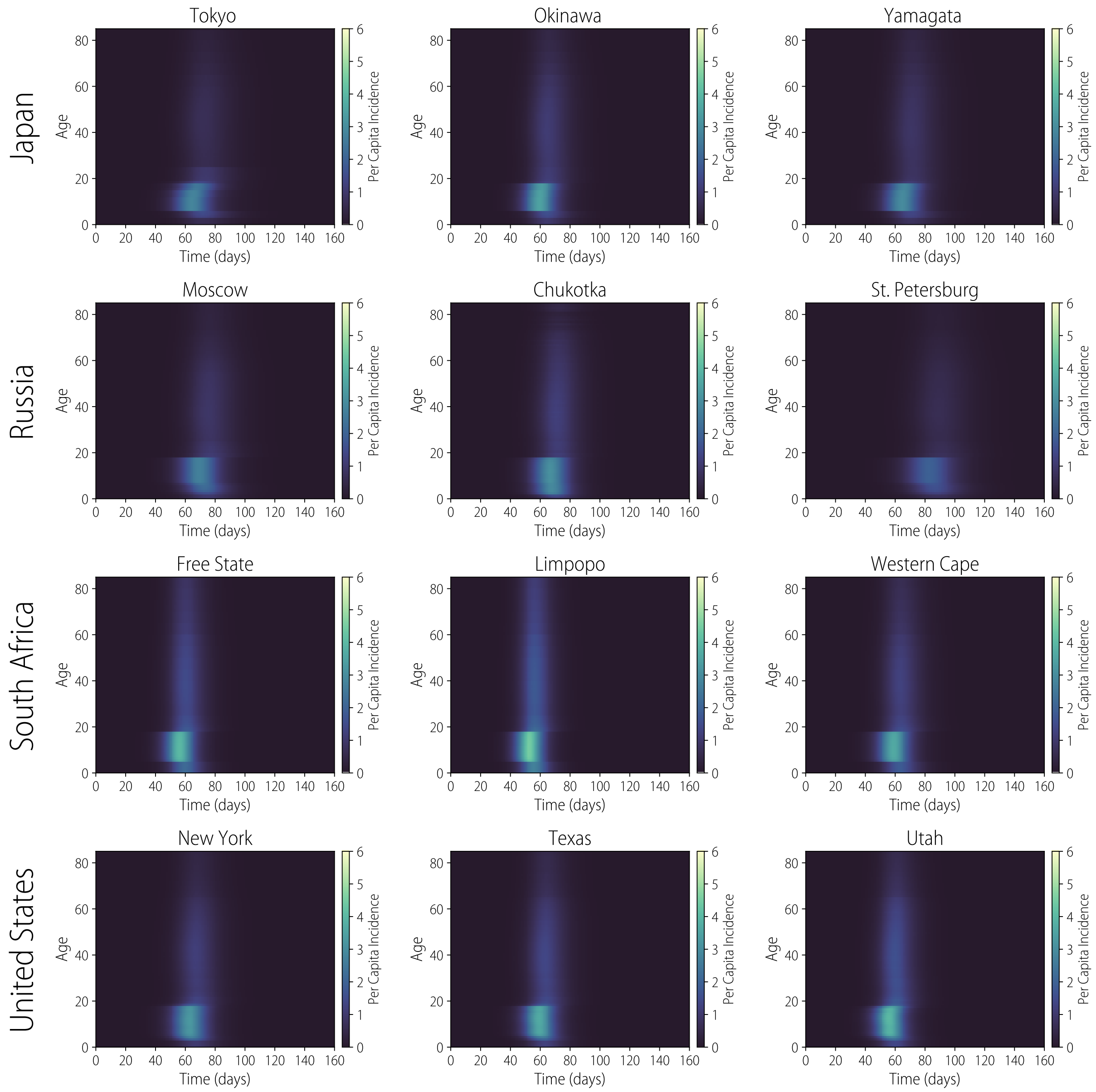

Supplementary Figure 41: Heatmap of the incidence of new infection by age over time for a subset of locations. Given the different population sizes among the locations, in order to allow the comparison of the temporal dynamics of the epidemic, the same initial prevalence of infectious individuals is used in all locations. The parameters β and χ are fixed in all locations are equal to the average values computed over the posterior distributions obtained for the five locations included in the analysis of 2009 H1N1 influenza pandemic seroprevalence data.

Figures 42, 43, 44,45, 46, 47, 48, and 49 shows for each country in our study: i) the estimated attack rates using the location-specific overall contact matrix vs. the national overall matrix as proxy for the subnational patterns, and ii) a map of the percentage variation of the attack rate using the location-specific matrix with respect to using the national contact matrix as a proxy for the subnational patterns. The percent variation is calculated as $(\mathrm{AR}_c - \mathrm{AR}_l)/\mathrm{AR}_c$, where AR_c is the estimated attack rate using the national- or country-level contact matrix, and AR_l is the estimated attack rate using the location-specific attack rate.

This analysis confirms confirms that results presented in the main text, i.e., a much lower variability when using national level contact matrices, a nonlinear relation between the estimated attack rates using the country-level mixing patterns and using location-specific data, and clear geographical trend. However, the patterns observed in the analysis here reported are much more marked than those shown in the main text as, once again, the inclusion of an age-dependent susceptibility to infection enhance the role of the socio-demographic features of the population on the epidemic dynamics.

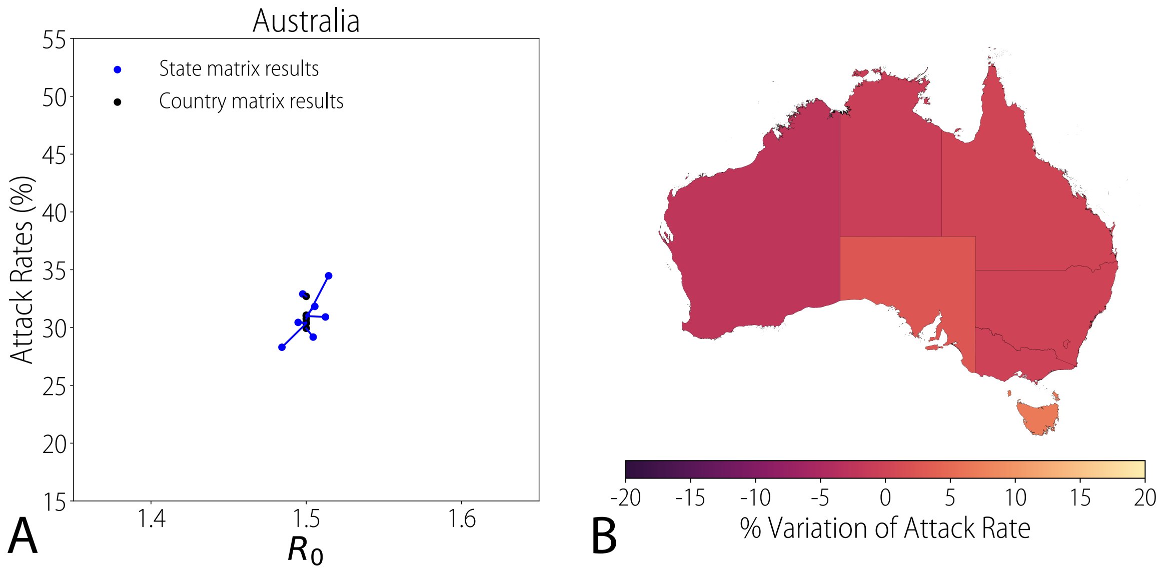

Supplementary Figure 42: **A** The black dots represent the estimated attack rates in each province of Australia by using the country-level contact matrix and the location-specific age structure of the population. Colored dots represent the estimated attack rates in each location by using both location-specific contact matrix and age structure of the population. The colored lines connect to the two estimated values attack rate for each location. The transmission rate is set such that $R_0 = 1.5$ when using the country-level matrix while χ is fixed to the average value computed over the posterior distributions obtained for the five locations included in the analysis of 2009 H1N1 influenza pandemic seroprevalence data. **B** Map showing the percentage variation of the attack rate using the location-specific contact matrix with respect to using the national contact matrix as a proxy for the subnational contact patterns.

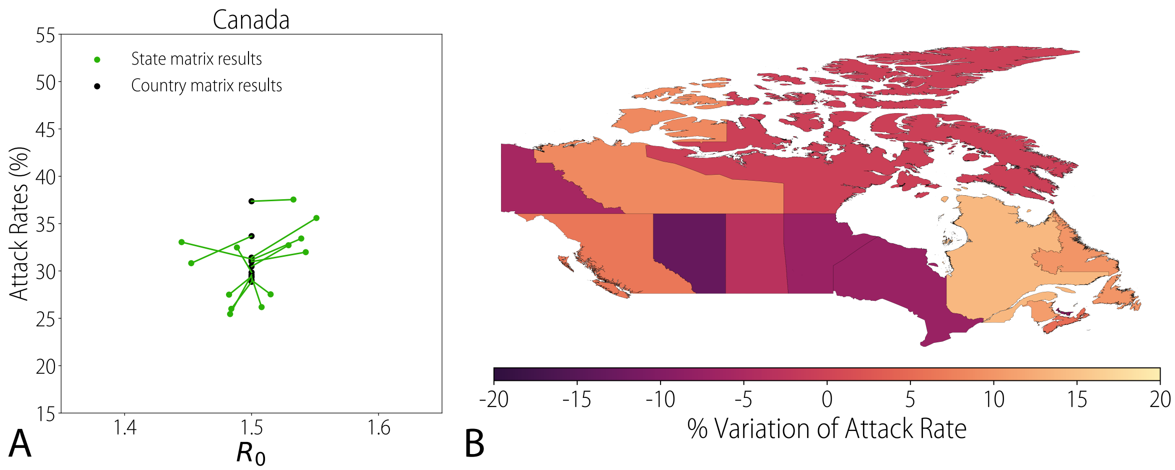

Supplementary Figure 43: As Fig. 42, but for Canada

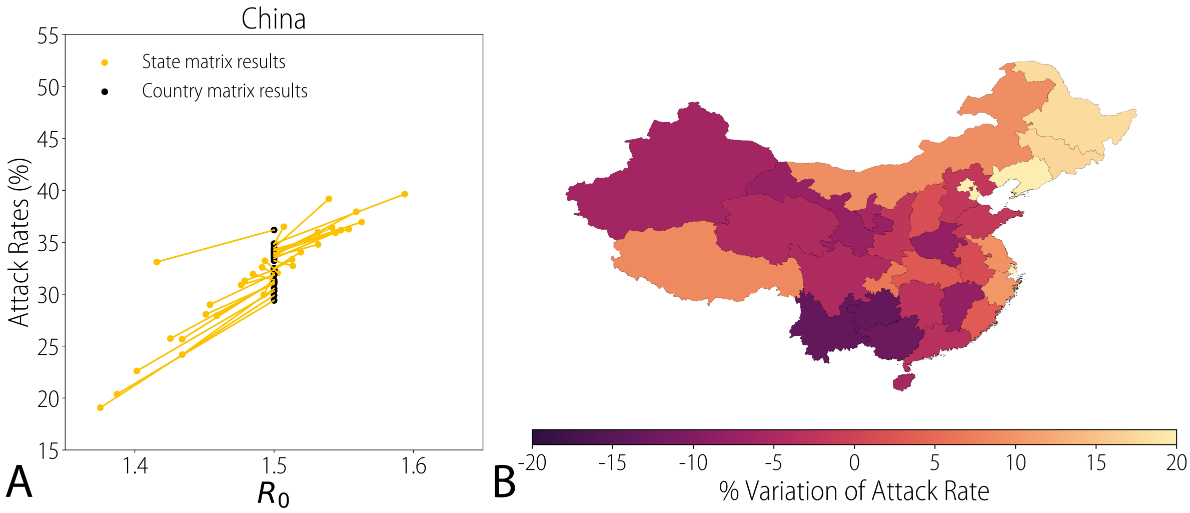

Supplementary Figure 44: As Fig. 42, but for China

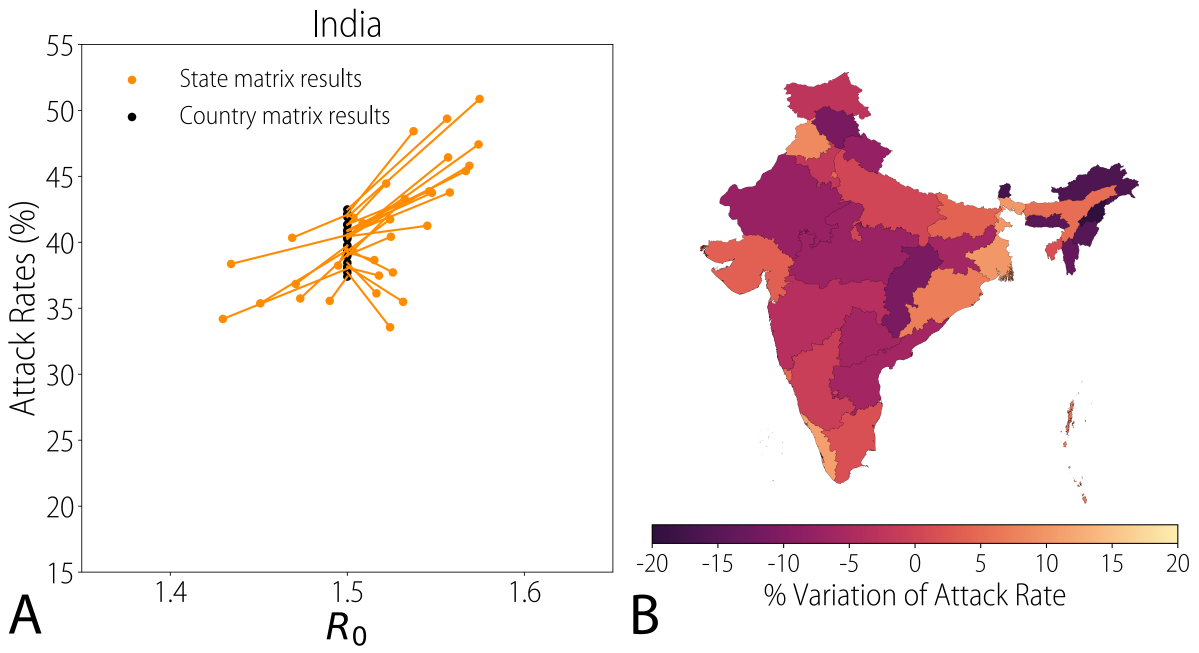

Supplementary Figure 45: As Fig. 42, but for India

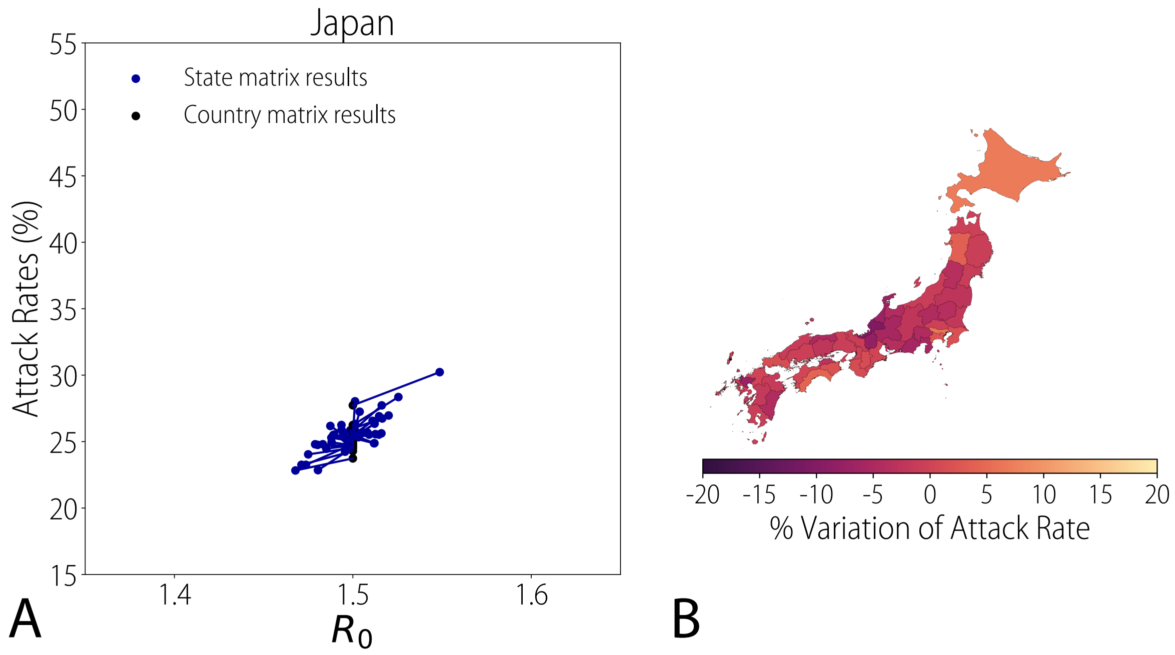

Supplementary Figure 46: As Fig. 42, but for Japan

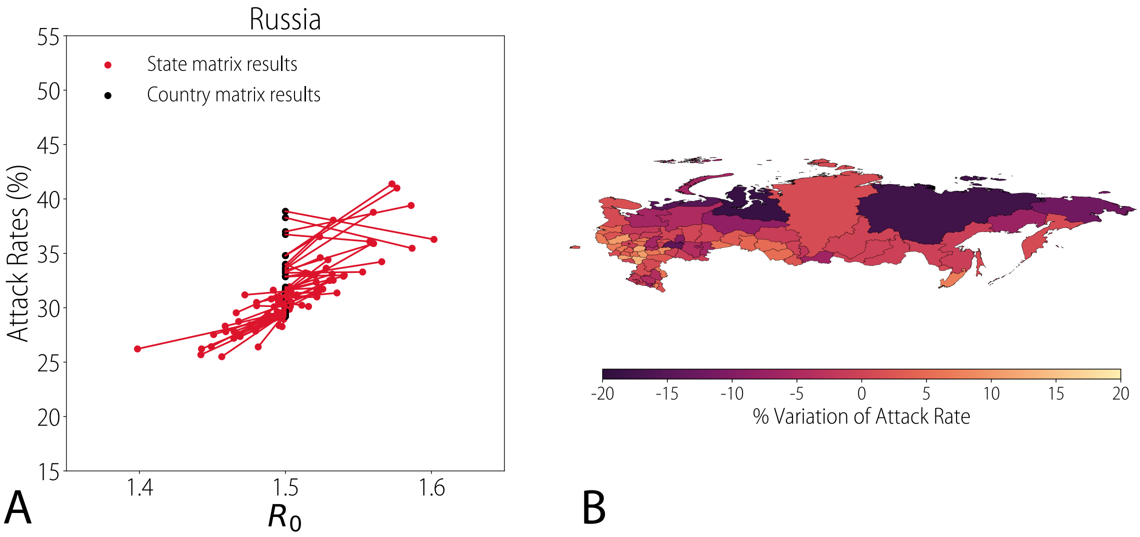

Supplementary Figure 47: As Fig. 42, but for Russia

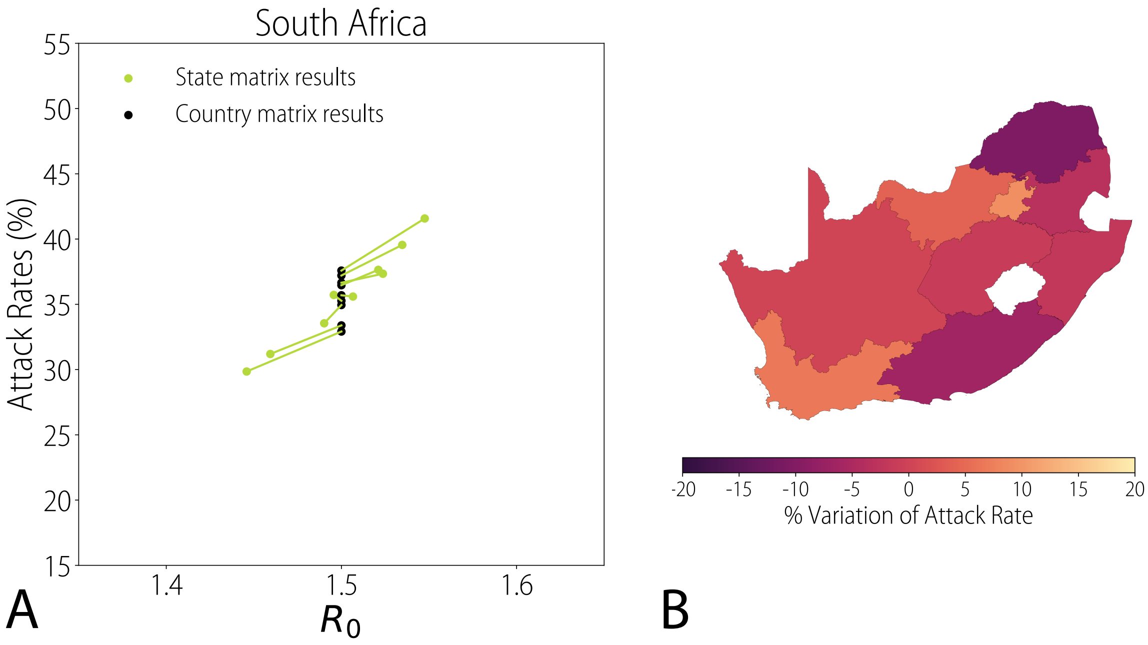

Supplementary Figure 48: As Fig. 42, but for South Africa

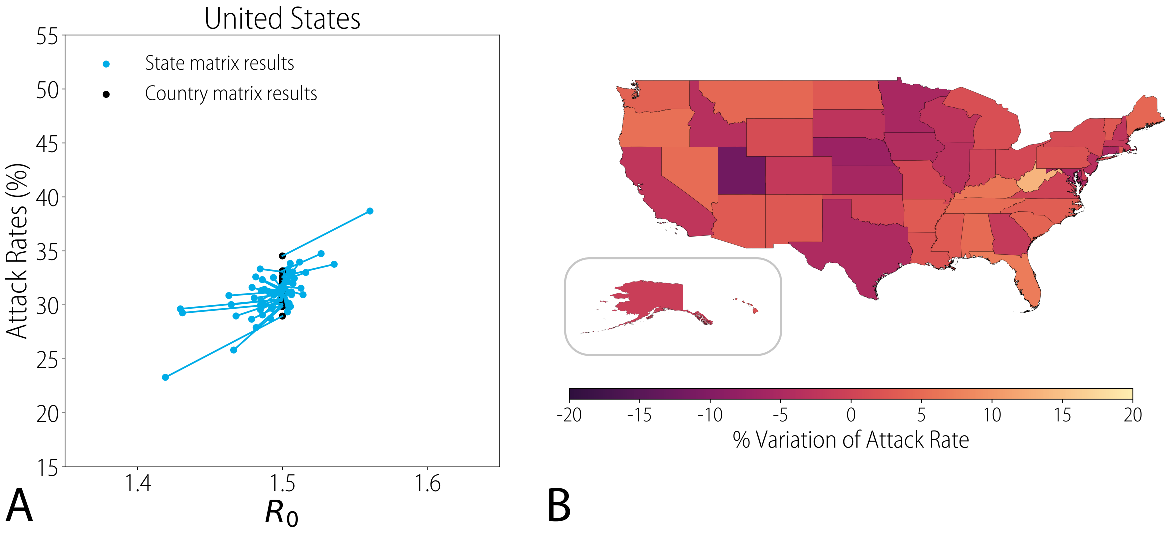

Supplementary Figure 49: As Fig. 42, but for the United States

4 Appendix

Age Structure: Census vs. Synthetic Pop. Statistical Tests

Location	Country	Pearson's r	KS	RMSE
Australian Capital Territory	AUS	1.00	0.05	0.00
New South Wales	AUS	1.00	0.08	0.00
Northern Territory	AUS	1.00	0.06	0.00
Queensland	AUS	1.00	0.06	0.00
South Australia	AUS	1.00	0.07	0.00
Tasmania	AUS	1.00	0.06	0.00
Victoria	AUS	1.00	0.09	0.00
Western Australia	AUS	1.00	0.06	0.00
Alberta	CAN	1.00	0.06	0.00
British Columbia	CAN	0.99	0.06	0.00
Manitoba	CAN	0.98	0.12	0.00
New Brunswick	CAN	0.98	0.06	0.00
Newfoundland & Labrador	CAN	0.98	0.08	0.01
Nova Scotia	CAN	0.98	0.07	0.00
Ontario	CAN	1.00	0.07	0.00
Prince Edward Island	CAN	0.93	0.09	0.02
Quebec	CAN	0.99	0.07	0.00
Saskatchewan	CAN	0.98	0.11	0.00
Yukon	CAN	0.95	0.12	0.03
Anhui	CHN	0.99	0.11	0.11
Beijing	CHN	1.00	0.11	0.12
China	CHN	1.00	0.11	0.06
Chongqing	CHN	0.99	0.11	0.13
Fujian	CHN	1.00	0.11	0.04
Gansu	CHN	1.00	0.11	0.05
Guangdong	CHN	1.00	0.11	0.05
Guangxi	CHN	0.99	0.11	0.07
Guizhou	CHN	0.98	0.11	0.41
Hainan	CHN	1.00	0.05	0.04
Hebei	CHN	1.00	0.16	0.06
Heilongjiang	CHN	1.00	0.05	0.01
Henan	CHN	0.99	0.11	0.10
Hubei	CHN	1.00	0.05	0.02
Hunan	CHN	1.00	0.05	0.03
Inner Mongolia	CHN	1.00	0.05	0.02
Jiangsu	CHN	1.00	0.11	0.02
Jiangxi	CHN	1.00	0.11	0.05
Jilin	CHN	1.00	0.05	0.02
Liaoning	CHN	1.00	0.11	0.02
Ningxia	CHN	0.99	0.11	0.21
Qinghai	CHN	1.00	0.11	0.05
Shaanxi	CHN	1.00	0.11	0.04
Shandong	CHN	1.00	0.11	0.06
Shanghai	CHN	1.00	0.11	0.04
Shanxi	CHN	0.99	0.11	0.13
Sichuan	CHN	1.00	0.11	0.06
Tianjin	CHN	1.00	0.16	0.11
Tibet	CHN	1.00	0.11	0.02
Xinjiang	CHN	1.00	0.11	0.10
Yunnan	CHN	1.00	0.11	0.05
Zhejiang	CHN	1.00	0.11	0.03
Uttar Pradesh	IND	0.97	0.08	0.05
Rajasthan	IND	0.96	0.06	0.05
Haryana	IND	0.97	0.08	0.03
Puducherry	IND	0.99	0.06	0.01
West Bengal	IND	0.98	0.09	0.02
Andhra Pradesh	IND	0.98	0.06	0.02
Arunachal Pradesh	IND	0.99	0.07	0.02
Sikkim	IND	0.99	0.05	0.01
Assam	IND	0.98	0.07	0.02
Uttarakhand	IND	0.98	0.07	0.02
Tamil Nadu	IND	0.99	0.07	0.01
Mizoram	IND	0.99	0.08	0.01
Himachal Pradesh	IND	0.99	0.08	0.01
Meghalaya	IND	0.99	0.06	0.01
Gujarat	IND	0.99	0.08	0.01
Odisha	IND	0.98	0.06	0.02
Maharashtra	IND	0.98	0.06	0.01
Nct of Delhi	IND	0.98	0.06	0.02
Chhattisgarh	IND	0.98	0.07	0.02
Manipur	IND	0.99	0.07	0.01
Bihar	IND	0.97	0.06	0.06
Tripura	IND	0.99	0.07	0.01
Jammu & Kashmir	IND	0.99	0.06	0.02
Punjab	IND	0.98	0.07	0.02
Goa	IND	0.99	0.08	0.01
Madhya Pradesh	IND	0.97	0.06	0.03
Jharkhand	IND	0.97	0.07	0.04
Andaman & Nicobar Islands	IND	0.99	0.05	0.01
Karnataka	IND	0.98	0.06	0.02
Daman & Diu	IND	0.98	0.05	0.04
Nagaland	IND	0.99	0.06	0.01
Kerala	IND	0.98	0.12	0.01
Israel	ISR	0.99	0.08	0.00
Aichi	JPN	1.00	0.06	0.00
Akita	JPN	1.00	0.06	0.00
Aomori	JPN	1.00	0.07	0.00
Chiba	JPN	1.00	0.05	0.00
Ehime	JPN	1.00	0.05	0.00
Fukui	JPN	1.00	0.06	0.00
Fukuoka	JPN	1.00	0.05	0.00
Fukushima	JPN	1.00	0.05	0.00
Gifu	JPN	1.00	0.06	0.00
Gumma	JPN	1.00	0.05	0.00
Hiroshima	JPN	1.00	0.05	0.00
Hokkaido	JPN	1.00	0.08	0.00
Hyogo	JPN	1.00	0.04	0.00
Ibaraki	JPN	1.00	0.04	0.00
Ishikawa	JPN	1.00	0.06	0.00
Iwate	JPN	1.00	0.07	0.00
Kagawa	JPN	1.00	0.05	0.00
Kagoshima	JPN	1.00	0.06	0.00
Kanagawa	JPN	1.00	0.05	0.00
Kochi	JPN	1.00	0.05	0.00
Kumamoto	JPN	1.00	0.05	0.00
Kyoto	JPN	1.00	0.05	0.00
Mie	JPN	1.00	0.05	0.00
Miyagi	JPN	1.00	0.05	0.00
Miyazaki	JPN	1.00	0.06	0.00
Nagano	JPN	1.00	0.04	0.00
Nagasaki	JPN	1.00	0.06	0.00
Nara	JPN	1.00	0.08	0.00
Niigata	JPN	1.00	0.08	0.00
Oita	JPN	1.00	0.05	0.00
Okayama	JPN	1.00	0.06	0.00
Okinawa	JPN	1.00	0.07	0.00
Osaka	JPN	1.00	0.05	0.00
Saga	JPN	1.00	0.07	0.00
Saitama	JPN	1.00	0.04	0.00
Shiga	JPN	1.00	0.05	0.00
Shimane	JPN	1.00	0.07	0.00
Shizuoka	JPN	1.00	0.05	0.00
Tochigi	JPN	1.00	0.06	0.00
Tokushima	JPN	1.00	0.05	0.00
Tokyo	JPN	1.00	0.07	0.00
Tottori	JPN	1.00	0.04	0.00
Toyama	JPN	1.00	0.05	0.00
Wakayama	JPN	1.00	0.06	0.00
Yamagata	JPN	1.00	0.06	0.00
Yamaguchi	JPN	1.00	0.04	0.00
Yamanashi	JPN	1.00	0.06	0.00
Adygea	RUS	0.99	0.11	0.00
Altai Krai	RUS	0.99	0.06	0.00
Altai Republic	RUS	0.99	0.07	0.00
Amur Oblast	RUS	1.00	0.06	0.00
Arkhangelsk Oblast	RUS	0.99	0.07	0.00
Astrakhan Oblast	RUS	0.99	0.08	0.00
Bashkortostan	RUS	0.99	0.09	0.00

Supplementary Table 6: Table of statistical tests comparing the census and synthetic population age distributions. Country codes are used to refer to the country (refer to table 1 for the country codes).

Age Structure: Census vs. Synthetic Pop. Statistical Tests

Location	Country	Pearson's r	KS	RMSE
Belgorod Oblast	RUS	0.98	0.08	0.00
Bryansk Oblast	RUS	0.99	0.07	0.00
Buryatia	RUS	1.00	0.06	0.00
Chechnya	RUS	0.99	0.09	0.01
Chelyabinsk Oblast	RUS	0.99	0.06	0.00
Chukotka	RUS	0.99	0.11	0.01
Chuvashia	RUS	0.99	0.11	0.00
Dagestan	RUS	0.99	0.06	0.00
Ingushetia	RUS	0.99	0.06	0.01
Irkutsk Oblast	RUS	0.99	0.06	0.00
Ivanovo Oblast	RUS	0.99	0.06	0.00
Jewish Auto. Oblast	RUS	0.99	0.06	0.00
Kabardino Balkaria	RUS	1.00	0.07	0.00
Kaliningrad Oblast	RUS	0.99	0.07	0.00
Kalmykia	RUS	1.00	0.08	0.00
Kaluga Oblast	RUS	0.99	0.06	0.00
Kamchatka Krai	RUS	1.00	0.05	0.00
Karachay Cherkessia	RUS	1.00	0.07	0.00
Karelia	RUS	0.99	0.04	0.00
Kemerovo Oblast	RUS	0.99	0.07	0.00
Khabarovsk Krai	RUS	1.00	0.06	0.00
Khakassia	RUS	1.00	0.06	0.00
Khanty Mansi Auto. Okrug	RUS	1.00	0.06	0.00
Kirov Oblast	RUS	0.99	0.05	0.00
Komi Republic	RUS	1.00	0.06	0.00
Kostroma Oblast	RUS	0.99	0.05	0.00
Krasnodar Krai	RUS	0.99	0.05	0.00
Krasnoyarsk Krai	RUS	0.99	0.05	0.00
Kurgan Oblast	RUS	0.99	0.07	0.00
Kursk Oblast	RUS	0.98	0.08	0.00
Leningrad Oblast	RUS	0.99	0.07	0.00
Lipetsk Oblast	RUS	0.99	0.07	0.00
Magadan Oblast	RUS	1.00	0.06	0.00
Mari El	RUS	0.99	0.07	0.00
Mordovia	RUS	0.99	0.06	0.00
Moscow Oblast	RUS	0.99	0.06	0.00
Moscow	RUS	0.99	0.11	0.00
Murmansk Oblast	RUS	1.00	0.07	0.00
Nenets Auto. Okrug	RUS	1.00	0.06	0.00
Nizhny Novgorod Oblast	RUS	0.99	0.07	0.00
North Ossetia Alania	RUS	0.99	0.09	0.00
Novgorod Oblast	RUS	0.99	0.05	0.00
Novosibirsk Oblast	RUS	0.99	0.07	0.00
Omsk Oblast	RUS	0.99	0.06	0.00
Orenburg Oblast	RUS	0.99	0.08	0.00
Oryol Oblast	RUS	0.99	0.08	0.00
Penza Oblast	RUS	0.99	0.07	0.00
Perm Krai	RUS	0.99	0.07	0.00
Primorsky Krai	RUS	0.99	0.07	0.00
Pskov Oblast	RUS	0.99	0.09	0.00
Rostov Oblast	RUS	0.99	0.07	0.00
Russian Federation	RUS	0.99	0.07	0.00
Ryazan Oblast	RUS	0.98	0.09	0.00
Sakha	RUS	1.00	0.06	0.00
Sakhalin Oblast	RUS	1.00	0.06	0.00
Samara Oblast	RUS	0.99	0.06	0.00
Saratov Oblast	RUS	0.99	0.06	0.00
Smolensk Oblast	RUS	0.99	0.07	0.00
St. Petersburg	RUS	0.99	0.08	0.00
Stavropol Krai	RUS	0.99	0.06	0.00
Sverdlovsk Oblast	RUS	0.99	0.06	0.00
Tambov Oblast	RUS	0.99	0.11	0.00
Tatarstan	RUS	0.99	0.07	0.00
Tomsk Oblast	RUS	1.00	0.05	0.00
Tula Oblast	RUS	0.99	0.11	0.00
Tuva	RUS	1.00	0.05	0.00
Tver Oblast	RUS	0.99	0.08	0.00
Tyumen Oblast	RUS	1.00	0.07	0.00
Udmurtia	RUS	0.99	0.06	0.00
Ulyanovsk Oblast	RUS	0.99	0.09	0.00
Vladimir Oblast	RUS	0.99	0.07	0.00
Volgograd Oblast	RUS	0.99	0.07	0.00
Vologda Oblast	RUS	0.99	0.07	0.00
Voronezh Oblast	RUS	0.99	0.07	0.00
Yamalo Nenets Autonomous Okrug	RUS	1.00	0.07	0.00
Yaroslavl Oblast	RUS	0.99	0.08	0.00
Zabaykalsky Krai	RUS	1.00	0.06	0.00
Eastern Cape	ZAF	1.00	0.06	0.00
Free State	ZAF	1.00	0.06	0.00
Gauteng	ZAF	1.00	0.05	0.00
KwaZulu Natal	ZAF	1.00	0.06	0.00
Limpopo	ZAF	1.00	0.06	0.00
Mpumalanga	ZAF	1.00	0.05	0.00
Northern Cape	ZAF	1.00	0.07	0.00
North West	ZAF	1.00	0.05	0.00
Western Cape	ZAF	1.00	0.06	0.00
Alabama	USA	1.00	0.11	0.00
Alaska	USA	1.00	0.11	0.00
Arizona	USA	1.00	0.06	0.01
Arkansas	USA	1.00	0.17	0.01
California	USA	1.00	0.11	0.00
Colorado	USA	1.00	0.11	0.00
Connecticut	USA	1.00	0.06	0.00
Delaware	USA	1.00	0.11	0.01
District of Columbia	USA	1.00	0.11	0.05
Florida	USA	1.00	0.11	0.00
Georgia	USA	1.00	0.11	0.00
Hawaii	USA	1.00	0.11	0.00
Idaho	USA	1.00	0.11	0.01
Illinois	USA	1.00	0.11	0.00
Indiana	USA	1.00	0.11	0.01
Iowa	USA	1.00	0.11	0.01
Kansas	USA	1.00	0.17	0.01
Kentucky	USA	1.00	0.11	0.00
Louisiana	USA	1.00	0.11	0.00
Maine	USA	1.00	0.11	0.01
Maryland	USA	1.00	0.11	0.01
Massachusetts	USA	1.00	0.11	0.00
Michigan	USA	1.00	0.11	0.01
Minnesota	USA	1.00	0.11	0.01
Mississippi	USA	1.00	0.17	0.00
Missouri	USA	1.00	0.11	0.00
Montana	USA	1.00	0.28	0.03
Nebraska	USA	1.00	0.11	0.02
Nevada	USA	1.00	0.11	0.01
New Hampshire	USA	1.00	0.17	0.02
New Jersey	USA	1.00	0.06	0.00
New Mexico	USA	1.00	0.11	0.01
New York	USA	1.00	0.11	0.01
North Carolina	USA	1.00	0.17	0.00
North Dakota	USA	1.00	0.11	0.03
Ohio	USA	1.00	0.11	0.01
Oklahoma	USA	1.00	0.11	0.00
Oregon	USA	1.00	0.17	0.01
Pennsylvania	USA	1.00	0.06	0.00
Puerto Rico	USA	1.00	0.17	0.01
Rhode Island	USA	1.00	0.11	0.03
South Carolina	USA	1.00	0.17	0.00
South Dakota	USA	1.00	0.11	0.01
Tennessee	USA	1.00	0.17	0.01
Texas	USA	1.00	0.11	0.00
Utah	USA	1.00	0.17	0.01
Vermont	USA	1.00	0.11	0.01
Virginia	USA	1.00	0.11	0.00
Washington	USA	1.00	0.11	0.01
West Virginia	USA	1.00	0.11	0.01
Wisconsin	USA	1.00	0.11	0.01
Wyoming	USA	1.00	0.11	0.02

Supplementary Table 7: Table of statistical tests comparing the census and synthetic population age distributions. Country codes are used to refer to the country (refer to table 1 for the country codes).

Household Size Distributions: Census vs. Synthetic Pop. Statistical Tests

Location	Country	Pearson's r	KS	RMSE	Location	Country	Pearson's r	KS	RMSE
Australian Capital Territory	AUS	1.00	0.12	0.00	Chhattisgarh	IND	1.00	0.11	0.00
New South Wales	AUS	1.00	0.12	0.01	Manipur	IND	1.00	0.11	0.00
Northern Territory	AUS	1.00	0.25	0.83	Bihar	IND	1.00	0.22	0.00
Queensland	AUS	1.00	0.12	0.02	Tripura	IND	1.00	0.11	0.00
South Australia	AUS	1.00	0.12	0.01	Jammu & Kashmir	IND	1.00	0.11	0.00
Tasmania	AUS	1.00	0.12	0.00	Punjab	IND	1.00	0.11	0.00
Victoria	AUS	1.00	0.12	0.00	Goa	IND	1.00	0.11	0.00
Western Australia	AUS	1.00	0.12	0.00	Madhya Pradesh	IND	1.00	0.11	0.00
Alberta	CAN	1.00	0.00	0.00	Jharkhand	IND	1.00	0.11	0.00
British Columbia	CAN	1.00	0.00	0.00	Andaman & Nicobar Islands	IND	1.00	0.11	0.00
Manitoba	CAN	1.00	0.00	0.00	Karnataka	IND	1.00	0.11	0.00
New Brunswick	CAN	1.00	0.00	0.00	Daman & Diu	IND	1.00	0.11	0.00
Newfoundland & Labrador	CAN	1.00	0.00	0.00	Nagaland	IND	1.00	0.11	0.00
Nova Scotia	CAN	1.00	0.00	0.00	Kerala	IND	1.00	0.11	0.00
Ontario	CAN	1.00	0.00	0.00	Israel	ISR	1.00	0.14	0.16
Prince Edward Island	CAN	1.00	0.00	0.00	Aichi	JPN	0.99	0.10	3.35
Quebec	CAN	1.00	0.00	0.00	Akita	JPN	1.00	0.10	0.57
Saskatchewan	CAN	1.00	0.00	0.00	Aomori	JPN	1.00	0.20	0.88
Yukon	CAN	1.00	0.18	0.00	Chiba	JPN	1.00	0.20	2.15
Anhui	CHN	1.00	0.10	0.00	Ehime	JPN	0.99	0.20	4.92
Beijing	CHN	1.00	0.10	0.00	Fukui	JPN	1.00	0.20	1.75
China	CHN	1.00	0.10	0.00	Fukuoka	JPN	0.97	0.10	9.70
Chongqing	CHN	1.00	0.10	0.00	Fukushima	JPN	1.00	0.10	1.08
Fujian	CHN	1.00	0.10	0.00	Gifu	JPN	1.00	0.20	0.91
Gansu	CHN	1.00	0.10	0.00	Gumma	JPN	1.00	0.20	0.09
Guangdong	CHN	1.00	0.10	0.00	Hiroshima	JPN	0.99	0.20	4.84
Guangxi	CHN	1.00	0.10	0.00	Hokkaido	JPN	0.97	0.20	13.44
Guizhou	CHN	1.00	0.10	0.00	Hyogo	JPN	0.99	0.20	2.45
Hainan	CHN	1.00	0.10	0.01	Ibaraki	JPN	1.00	0.10	0.00
Hebei	CHN	1.00	0.10	0.00	Ishikawa	JPN	1.00	0.10	1.28
Heilongjiang	CHN	1.00	0.10	0.00	Iwate	JPN	1.00	0.10	0.93
Henan	CHN	1.00	0.10	0.00	Kagawa	JPN	1.00	0.20	1.72
Hubei	CHN	1.00	0.10	0.00	Kagoshima	JPN	0.98	0.20	10.01
Hunan	CHN	1.00	0.10	0.01	Kanagawa	JPN	0.99	0.20	6.05
Inner Mongolia	CHN	1.00	0.10	0.00	Kochi	JPN	0.98	0.20	9.83
Jiangsu	CHN	1.00	0.10	0.00	Kumamoto	JPN	1.00	0.20	1.14
Jiangxi	CHN	1.00	0.10	0.00	Kyoto	JPN	0.97	0.20	11.50
Jilin	CHN	1.00	0.10	0.01	Mie	JPN	1.00	0.20	0.36
Liaoning	CHN	1.00	0.10	0.00	Miyagi	JPN	0.98	0.10	4.71
Ningxia	CHN	1.00	0.10	0.00	Miyazaki	JPN	0.99	0.20	4.78
Qinghai	CHN	1.00	0.10	0.00	Nagano	JPN	1.00	0.10	0.25
Shaanxi	CHN	1.00	0.10	0.00	Nagasaki	JPN	0.99	0.20	3.06
Shandong	CHN	1.00	0.10	0.00	Nara	JPN	0.99	0.10	1.79
Shanghai	CHN	1.00	0.10	0.00	Niigata	JPN	1.00	0.20	0.51
Shanxi	CHN	1.00	0.10	0.00	Oita	JPN	0.99	0.20	4.18
Sichuan	CHN	1.00	0.10	0.00	Okayama	JPN	0.99	0.10	1.85
Tianjin	CHN	1.00	0.10	0.00	Okinawa	JPN	0.99	0.10	2.79
Tibet	CHN	1.00	0.10	0.01	Osaka	JPN	0.98	0.20	10.03
Xinjiang	CHN	1.00	0.10	0.01	Saga	JPN	1.00	0.20	0.62
Yunnan	CHN	1.00	0.10	0.00	Saitama	JPN	1.00	0.20	0.80
Zhejiang	CHN	1.00	0.10	0.01	Shiga	JPN	1.00	0.10	0.80
Uttar Pradesh	IND	1.00	0.11	0.00	Shimane	JPN	1.00	0.20	1.24
Rajasthan	IND	1.00	0.11	0.00	Shizuoka	JPN	1.00	0.10	0.01
Haryana	IND	1.00	0.11	0.00	Tochigi	JPN	1.00	0.10	0.06
Puducherry	IND	1.00	0.11	0.00	Tokushima	JPN	0.99	0.20	2.03
West Bengal	IND	1.00	0.11	0.00	Tokyo	JPN	0.91	0.20	42.65
Andhra Pradesh	IND	1.00	0.11	0.00	Tottori	JPN	1.00	0.10	0.47
Arunachal Pradesh	IND	1.00	0.11	0.00	Toyama	JPN	1.00	0.20	0.77
Sikkim	IND	1.00	0.11	0.00	Wakayama	JPN	1.00	0.20	1.76
Assam	IND	1.00	0.11	0.00	Yamagata	JPN	1.00	0.20	2.12
Uttarakhand	IND	1.00	0.11	0.00	Yamaguchi	JPN	0.99	0.20	5.84
Tamil Nadu	IND	1.00	0.11	0.00	Yamanashi	JPN	1.00	0.20	0.32
Mizoram	IND	1.00	0.11	0.00	Adygea	RUS	1.00	0.20	0.01
Himachal Pradesh	IND	1.00	0.11	0.00	Altai Krai	RUS	1.00	0.10	0.00
Meghalaya	IND	1.00	0.11	0.00	Altai Republic	RUS	1.00	0.20	0.01
Gujarat	IND	1.00	0.11	0.00	Amur Oblast	RUS	1.00	0.10	0.00
Odisha	IND	1.00	0.11	0.00	Arkhangelsk Oblast	RUS	1.00	0.10	0.00
Maharashtra	IND	1.00	0.11	0.00	Astrakhan Oblast	RUS	1.00	0.20	0.01
Nct of Delhi	IND	1.00	0.11	0.00	Bashkortostan	RUS	1.00	0.10	0.00

Supplementary Table 8: Table of statistical tests comparing the census and synthetic population household size distributions. Country codes are used to refer to the country name (see Table 1 for the country codes).

Household Size Distributions: Census vs. Synthetic Pop. Statistical Tests

Location	Country	Pearson's r	KS	RMSE
Belgorod Oblast	RUS	1.00	0.10	0.00
Bryansk Oblast	RUS	1.00	0.20	0.01
Buryatia	RUS	1.00	0.10	0.01
Chechnya	RUS	1.00	0.10	0.00
Chelyabinsk Oblast	RUS	1.00	0.10	0.01
Chukotka	RUS	1.00	0.20	0.02
Chuvashia	RUS	1.00	0.10	0.00
Dagestan	RUS	1.00	0.10	0.00
Ingushetia	RUS	1.00	0.10	0.01
Irkutsk Oblast	RUS	1.00	0.20	0.00
Ivanovo Oblast	RUS	1.00	0.20	0.00
Jewish Auto. Oblast	RUS	1.00	0.20	0.01
Kabardino Balkaria	RUS	1.00	0.10	0.01
Kaliningrad Oblast	RUS	1.00	0.10	0.00
Kalmykia	RUS	1.00	0.20	0.00
Kaluga Oblast	RUS	1.00	0.10	0.00
Kamchatka Krai	RUS	1.00	0.20	0.00
Karachay Cherkessia	RUS	1.00	0.10	0.01
Karelia	RUS	1.00	0.10	0.00
Kemerovo Oblast	RUS	1.00	0.10	0.00
Khabarovsk Krai	RUS	1.00	0.20	0.00
Khakassia	RUS	1.00	0.20	0.01
Khanty Mansi Auto. Okrug	RUS	1.00	0.10	0.00
Kirov Oblast	RUS	1.00	0.10	0.00
Komi Republic	RUS	1.00	0.10	0.00
Kostroma Oblast	RUS	1.00	0.20	0.00
Krasnodar Krai	RUS	1.00	0.20	0.00
Krasnoyarsk Krai	RUS	1.00	0.10	0.00
Kurgan Oblast	RUS	1.00	0.10	0.00
Kursk Oblast	RUS	1.00	0.10	0.00
Leningrad Oblast	RUS	1.00	0.20	0.00
Lipetsk Oblast	RUS	1.00	0.10	0.01
Magadan Oblast	RUS	1.00	0.20	0.00
Mari El	RUS	1.00	0.10	0.00
Mordovia	RUS	1.00	0.10	0.00
Moscow Oblast	RUS	1.00	0.10	0.00
Moscow	RUS	1.00	0.20	0.00
Murmansk Oblast	RUS	1.00	0.20	0.00
Nenets Auto. Okrug	RUS	1.00	0.20	0.03
Nizhny Novgorod Oblast	RUS	1.00	0.10	0.00
North Ossetia Alania	RUS	1.00	0.20	0.01
Novgorod Oblast	RUS	1.00	0.20	0.01
Novosibirsk Oblast	RUS	1.00	0.10	0.00
Omsk Oblast	RUS	1.00	0.10	0.00
Orenburg Oblast	RUS	1.00	0.10	0.00
Oryol Oblast	RUS	1.00	0.10	0.00
Penza Oblast	RUS	1.00	0.10	0.01
Perm Krai	RUS	1.00	0.10	0.00
Primorsky Krai	RUS	1.00	0.10	0.00
Pskov Oblast	RUS	1.00	0.20	0.01
Rostov Oblast	RUS	1.00	0.20	0.01
Russian Federation	RUS	1.00	0.20	0.01
Ryazan Oblast	RUS	1.00	0.20	0.00
Sakha	RUS	1.00	0.20	0.00
Sakhalin Oblast	RUS	1.00	0.20	0.00
Samara Oblast	RUS	1.00	0.10	0.00
Saratov Oblast	RUS	1.00	0.10	0.00
Smolensk Oblast	RUS	1.00	0.10	0.00
St. Petersburg	RUS	1.00	0.10	0.01
Stavropol Krai	RUS	1.00	0.10	0.01
Sverdlovsk Oblast	RUS	1.00	0.10	0.01
Tambov Oblast	RUS	1.00	0.10	0.00
Tatarstan	RUS	1.00	0.10	0.01
Tomsk Oblast	RUS	1.00	0.10	0.01
Tula Oblast	RUS	1.00	0.10	0.00
Tuva	RUS	1.00	0.10	0.00
Tver Oblast	RUS	1.00	0.10	0.00
Tyumen Oblast	RUS	1.00	0.10	0.00
Udmurtia	RUS	1.00	0.10	0.00
Ulyanovsk Oblast	RUS	1.00	0.20	0.00
Vladimir Oblast	RUS	1.00	0.20	0.00
Volgograd Oblast	RUS	1.00	0.20	0.00
Vologda Oblast	RUS	1.00	0.10	0.01
Voronezh Oblast	RUS	1.00	0.10	0.01
Yamalo Nenets Auto. Okrug	RUS	1.00	0.10	0.01
Yaroslavl Oblast	RUS	1.00	0.10	0.00
Zabaykalsky Krai	RUS	1.00	0.10	0.00
Eastern Cape	ZAF	1.00	0.10	0.12
Free State	ZAF	1.00	0.10	0.01
Gauteng	ZAF	1.00	0.10	0.03
KwaZulu Natal	ZAF	1.00	0.10	0.11
Limpopo	ZAF	1.00	0.10	0.02
Mpumalanga	ZAF	1.00	0.10	0.03
Northern Cape	ZAF	1.00	0.10	0.03
North West	ZAF	1.00	0.10	0.03
Western Cape	ZAF	1.00	0.20	0.03
Alabama	USA	1.00	0.14	0.00
Alaska	USA	1.00	0.14	0.00
Arizona	USA	1.00	0.14	0.00
Arkansas	USA	1.00	0.14	0.00
California	USA	1.00	0.14	0.00
Colorado	USA	1.00	0.14	0.00
Connecticut	USA	1.00	0.14	0.00
Delaware	USA	1.00	0.14	0.00
District of Columbia	USA	1.00	0.14	0.00
Florida	USA	1.00	0.14	0.00
Georgia	USA	1.00	0.14	0.00
Hawaii	USA	1.00	0.14	0.00
Idaho	USA	1.00	0.14	0.00
Illinois	USA	1.00	0.14	0.00
Indiana	USA	1.00	0.14	0.00
Iowa	USA	1.00	0.14	0.00
Kansas	USA	1.00	0.14	0.00
Kentucky	USA	1.00	0.14	0.00
Louisiana	USA	1.00	0.14	0.00
Maine	USA	1.00	0.14	0.00
Maryland	USA	1.00	0.14	0.00
Massachusetts	USA	1.00	0.14	0.00
Michigan	USA	1.00	0.14	0.00
Minnesota	USA	1.00	0.14	0.00
Mississippi	USA	1.00	0.14	0.00
Missouri	USA	1.00	0.14	0.00
Montana	USA	1.00	0.14	0.00
Nebraska	USA	1.00	0.14	0.00
Nevada	USA	1.00	0.14	0.00
New Hampshire	USA	1.00	0.14	0.00
New Jersey	USA	1.00	0.14	0.00
New Mexico	USA	1.00	0.14	0.00
New York	USA	1.00	0.14	0.00
North Carolina	USA	1.00	0.14	0.00
North Dakota	USA	1.00	0.14	0.00
Ohio	USA	1.00	0.14	0.00
Oklahoma	USA	1.00	0.14	0.00
Oregon	USA	1.00	0.14	0.00
Pennsylvania	USA	1.00	0.14	0.00
Puerto Rico	USA	1.00	0.14	0.00
Rhode Island	USA	1.00	0.14	0.00
South Carolina	USA	1.00	0.14	0.00
South Dakota	USA	1.00	0.14	0.00
Tennessee	USA	1.00	0.14	0.00
Texas	USA	1.00	0.14	0.00
Utah	USA	1.00	0.14	0.00
Vermont	USA	1.00	0.14	0.00
Virginia	USA	1.00	0.14	0.00
Washington	USA	1.00	0.14	0.00
West Virginia	USA	1.00	0.14	0.00
Wisconsin	USA	1.00	0.14	0.00
Wyoming	USA	1.00	0.14	0.00

Supplementary Table 9: Table of statistical tests comparing the census and synthetic population household size distributions. Country codes are used to refer to the country name (see Table 1 for the country codes).

Enrollment Rates: Census vs. Synthetic Pop. Statistical Tests

Location	Country	Pearson's r	KS	RMSE
Australian Capital Territory	AUS	1.00	0.04	0.11
New South Wales	AUS	1.00	0.05	0.07
Northern Territory	AUS	1.00	0.05	0.07
Queensland	AUS	1.00	0.05	0.08
South Australia	AUS	1.00	0.04	0.10
Tasmania	AUS	1.00	0.04	0.13
Victoria	AUS	1.00	0.05	0.06
Western Australia	AUS	1.00	0.05	0.07
Alberta	CAN	1.00	0.05	0.00
British Columbia	CAN	1.00	0.05	0.09
Manitoba	CAN	1.00	0.05	0.01
New Brunswick	CAN	1.00	0.05	0.00
Newfoundland & Labrador	CAN	1.00	0.05	0.01
Nova Scotia	CAN	1.00	0.05	0.01
Ontario	CAN	1.00	0.05	0.00
Prince Edward Island	CAN	1.00	0.68	44.81
Quebec	CAN	1.00	0.05	0.00
Saskatchewan	CAN	1.00	0.05	0.00
Yukon	CAN	1.00	0.05	0.03
Anhui	CHN	1.00	0.11	0.26
Beijing	CHN	1.00	0.11	0.09
China	CHN	1.00	0.11	0.06
Chongqing	CHN	1.00	0.11	0.10
Fujian	CHN	1.00	0.13	0.06
Gansu	CHN	1.00	0.11	0.07
Guangdong	CHN	1.00	0.09	0.05
Guangxi	CHN	1.00	0.11	0.10
Guizhou	CHN	1.00	0.13	0.54
Hainan	CHN	1.00	0.07	0.12
Hebei	CHN	1.00	0.11	0.05
Heilongjiang	CHN	1.00	0.11	0.05
Henan	CHN	1.00	0.07	0.04
Hubei	CHN	1.00	0.11	0.05
Hunan	CHN	1.00	0.11	0.05
Inner Mongolia	CHN	1.00	0.09	0.06
Jiangsu	CHN	1.00	0.13	0.07
Jiangxi	CHN	1.00	0.11	0.04
Jilin	CHN	1.00	0.07	0.05
Liaoning	CHN	1.00	0.09	0.07
Ningxia	CHN	1.00	0.09	0.03
Qinghai	CHN	1.00	0.17	8.67
Shaanxi	CHN	1.00	0.13	0.04
Shandong	CHN	1.00	0.17	0.04
Shanghai	CHN	1.00	0.20	0.08
Shanxi	CHN	1.00	0.11	0.04
Sichuan	CHN	1.00	0.09	0.06
Tianjin	CHN	1.00	0.17	0.08
Tibet	CHN	1.00	0.22	62.48
Xinjiang	CHN	1.00	0.17	0.04
Yunnan	CHN	1.00	0.15	3.34
Zhejiang	CHN	1.00	0.13	0.15
Uttar Pradesh	IND	1.00	0.10	0.18
Rajasthan	IND	1.00	0.05	0.36
Haryana	IND	1.00	0.10	0.52
Puducherry	IND	1.00	0.10	2.79
West Bengal	IND	1.00	0.05	0.28
Andhra Pradesh	IND	1.00	0.05	1.40
Arunachal Pradesh	IND	1.00	0.10	1.09
Sikkim	IND	1.00	0.24	22.51
Assam	IND	1.00	0.10	1.24
Uttarakhand	IND	1.00	0.10	1.10
Tamil Nadu	IND	1.00	0.05	2.66
Mizoram	IND	1.00	0.10	4.42
Himachal Pradesh	IND	0.99	0.33	49.59
Meghalaya	IND	1.00	0.10	2.06
Gujarat	IND	1.00	0.10	0.24
Odisha	IND	1.00	0.05	0.20
Maharashtra	IND	1.00	0.10	2.11
Nct of Delhi	IND	1.00	0.10	0.60

Location	Country	Pearson's r	KS	RMSE
Chhattisgarh	IND	1.00	0.10	2.99
Manipur	IND	1.00	0.10	3.32
Bihar	IND	1.00	0.10	0.17
Tripura	IND	1.00	0.10	1.23
Jammu & Kashmir	IND	1.00	0.10	0.35
Punjab	IND	1.00	0.05	1.25
Goa	IND	1.00	0.10	4.95
Madhya Pradesh	IND	1.00	0.10	0.79
Jharkhand	IND	1.00	0.10	0.49
Andaman & Nicobar Islands	IND	1.00	0.14	2.82
Karnataka	IND	1.00	0.10	1.69
Daman & Diu	IND	1.00	0.10	10.72
Nagaland	IND	1.00	0.24	14.61
Kerala	IND	1.00	0.10	1.02
Israel	ISR	1.00	0.18	1.00
Aichi	JPN	1.00	0.07	0.00
Akita	JPN	1.00	0.06	0.04
Aomori	JPN	1.00	0.06	0.01
Chiba	JPN	1.00	0.08	0.00
Ehime	JPN	1.00	0.06	0.03
Fukui	JPN	1.00	0.09	0.04
Fukuoka	JPN	1.00	0.09	0.01
Fukushima	JPN	1.00	0.07	0.02
Gifu	JPN	1.00	0.12	0.01
Gumma	JPN	1.00	0.05	0.02
Hiroshima	JPN	1.00	0.05	0.01
Hokkaido	JPN	1.00	0.11	0.01
Hyogo	JPN	1.00	0.06	0.01
Ibaraki	JPN	1.00	0.07	0.01
Ishikawa	JPN	1.00	0.08	0.06
Iwate	JPN	1.00	0.05	0.02
Kagawa	JPN	1.00	0.07	0.02
Kagoshima	JPN	1.00	0.04	0.02
Kanagawa	JPN	1.00	0.07	0.00
Kochi	JPN	1.00	0.06	0.03
Kumamoto	JPN	1.00	0.08	0.01
Kyoto	JPN	1.00	0.06	0.01
Mie	JPN	1.00	0.06	0.02
Miyagi	JPN	1.00	0.07	0.01
Miyazaki	JPN	1.00	0.06	0.10
Nagano	JPN	1.00	0.07	0.02
Nagasaki	JPN	1.00	0.05	0.02
Nara	JPN	1.00	0.06	0.01
Niigata	JPN	1.00	0.06	0.02
Oita	JPN	1.00	0.06	0.04
Okayama	JPN	1.00	0.06	0.01
Okinawa	JPN	1.00	0.07	0.02
Osaka	JPN	1.00	0.09	0.24
Saga	JPN	1.00	0.08	0.03
Saitama	JPN	1.00	0.08	0.00
Shiga	JPN	1.00	0.05	0.02
Shimane	JPN	1.00	0.04	0.07
Shizuoka	JPN	1.00	0.12	0.01
Tochigi	JPN	1.00	0.05	0.01
Tokushima	JPN	1.00	0.05	0.05
Tokyo	JPN	1.00	0.07	0.00
Tottori	JPN	1.00	0.07	0.05
Toyama	JPN	1.00	0.05	0.03
Wakayama	JPN	1.00	0.06	0.04
Yamagata	JPN	1.00	0.05	0.02
Yamaguchi	JPN	1.00	0.06	0.02
Yamanashi	JPN	1.00	0.08	0.02
Adygea	RUS	1.00	0.09	0.05
Altai Krai	RUS	1.00	0.10	0.06
Altai Republic	RUS	1.00	0.06	0.04
Amur Oblast	RUS	1.00	0.07	0.03
Arkhangelsk Oblast	RUS	1.00	0.07	0.05
Astrakhan Oblast	RUS	1.00	0.09	0.04
Bashkortostan	RUS	1.00	0.09	0.04

Supplementary Table 10: Table of statistical tests comparing the census and synthetic population enrollment rates. Country codes are used to refer to the country name (see Table 1 for the country codes).

Enrollment Rates: Census vs. Synthetic Pop. Statistical Tests

Location	Country	Pearson's r	KS	RMSE
Belgorod Oblast	RUS	1.00	0.10	0.05
Bryansk Oblast	RUS	1.00	0.07	0.04
Buryatia	RUS	1.00	0.06	0.05
Chechnya	RUS	1.00	0.06	0.02
Chelyabinsk Oblast	RUS	1.00	0.10	0.05
Chukotka	RUS	1.00	0.09	0.03
Chuvashia	RUS	1.00	0.09	0.05
Dagestan	RUS	1.00	0.07	0.01
Ingushetia	RUS	1.00	0.05	0.02
Irkutsk Oblast	RUS	1.00	0.08	0.03
Ivanovo Oblast	RUS	1.00	0.12	0.06
Jewish Auto. Oblast	RUS	1.00	0.09	0.05
Kabardino Balkaria	RUS	1.00	0.10	0.02
Kaliningrad Oblast	RUS	1.00	0.08	0.04
Kalmykia	RUS	1.00	0.08	0.04
Kaluga Oblast	RUS	1.00	0.10	0.07
Kamchatka Krai	RUS	1.00	0.10	0.04
Karachay Cherkessia	RUS	1.00	0.07	0.01
Karelia	RUS	1.00	0.09	0.08
Kemerovo Oblast	RUS	1.00	0.09	0.07
Khabarovsk Krai	RUS	1.00	0.10	0.05
Khakassia	RUS	1.00	0.09	0.04
Khanty Mansi Auto. Okrug	RUS	1.00	0.10	450.42
Kirov Oblast	RUS	1.00	0.08	0.05
Komi Republic	RUS	1.00	0.12	0.08
Kostroma Oblast	RUS	1.00	0.09	0.07
Krasnodar Krai	RUS	1.00	0.08	0.02
Krasnoyarsk Krai	RUS	1.00	0.09	0.04
Kurgan Oblast	RUS	1.00	0.08	0.08
Kursk Oblast	RUS	1.00	0.08	0.08
Leningrad Oblast	RUS	1.00	0.13	0.07
Lipetsk Oblast	RUS	1.00	0.11	0.04
Magadan Oblast	RUS	1.00	0.10	0.05
Mari El	RUS	1.00	0.09	0.07
Mordovia	RUS	1.00	0.09	0.07
Moscow Oblast	RUS	1.00	0.08	0.07
Moscow	RUS	1.00	0.09	0.04
Murmansk Oblast	RUS	1.00	0.06	0.04
Nenets Auto. Okrug	RUS	1.00	0.07	0.04
Nizhny Novgorod Oblast	RUS	1.00	0.09	0.05
North Ossetia Alania	RUS	1.00	0.10	352.93
Novgorod Oblast	RUS	1.00	0.08	0.09
Novosibirsk Oblast	RUS	1.00	0.08	0.07
Omsk Oblast	RUS	1.00	0.09	0.07
Orenburg Oblast	RUS	1.00	0.09	0.04
Oryol Oblast	RUS	1.00	0.08	0.05
Penza Oblast	RUS	1.00	0.09	0.05
Perm Krai	RUS	1.00	0.09	0.03
Primorsky Krai	RUS	1.00	0.10	0.06
Pskov Oblast	RUS	1.00	0.09	0.07
Rostov Oblast	RUS	1.00	0.09	0.03
Russian Federation	RUS	1.00	0.09	0.04
Ryazan Oblast	RUS	1.00	0.10	0.08
Sakha	RUS	1.00	0.07	0.02
Sakhalin Oblast	RUS	1.00	0.08	0.03
Samara Oblast	RUS	1.00	0.11	0.06
Saratov Oblast	RUS	1.00	0.10	0.05
Smolensk Oblast	RUS	1.00	0.09	0.08
St. Petersburg	RUS	1.00	0.08	652.72
Stavropol Krai	RUS	1.00	0.10	0.04
Sverdlovsk Oblast	RUS	1.00	0.11	0.05
Tambov Oblast	RUS	1.00	0.11	0.04
Tatarstan	RUS	1.00	0.06	0.04
Tomsk Oblast	RUS	1.00	0.09	0.04
Tula Oblast	RUS	1.00	0.10	0.06
Tuva	RUS	1.00	0.10	0.03
Tver Oblast	RUS	1.00	0.10	0.06
Tyumen Oblast	RUS	1.00	0.06	0.07
Udmurtia	RUS	1.00	0.09	0.04

Location	Country	Pearson's r	KS	RMSE
Ulyanovsk Oblast	RUS	1.00	0.11	0.04
Vladimir Oblast	RUS	1.00	0.09	0.06
Volgograd Oblast	RUS	1.00	0.11	0.08
Vologda Oblast	RUS	1.00	0.08	0.04
Voronezh Oblast	RUS	1.00	0.09	0.07
Yamalo Nenets Auto. Okrug	RUS	1.00	0.11	0.03
Yaroslavl Oblast	RUS	1.00	0.09	0.05
Zabaykalsky Krai	RUS	1.00	0.08	0.04
Eastern Cape	ZAF	1.00	0.09	0.00
Free State	ZAF	1.00	0.09	0.00
Gauteng	ZAF	1.00	0.09	0.00
KwaZulu Natal	ZAF	1.00	0.09	0.00
Limpopo	ZAF	1.00	0.09	0.00
Mpumalanga	ZAF	1.00	0.09	0.00
Northern Cape	ZAF	1.00	0.09	0.00
North West	ZAF	1.00	0.09	0.00
Western Cape	ZAF	1.00	0.09	0.00
Alabama	USA	1.00	0.12	0.04
Alaska	USA	1.00	0.12	0.12
Arizona	USA	1.00	0.12	0.09
Arkansas	USA	1.00	0.12	0.32
California	USA	1.00	0.12	0.07
Colorado	USA	1.00	0.12	0.09
Connecticut	USA	1.00	0.12	0.16
Delaware	USA	1.00	0.12	0.20
District of Columbia	USA	1.00	0.12	1.50
Florida	USA	1.00	0.12	0.15
Georgia	USA	1.00	0.12	0.12
Hawaii	USA	1.00	0.12	0.14
Idaho	USA	1.00	0.12	0.04
Illinois	USA	1.00	0.12	0.12
Indiana	USA	1.00	0.12	0.09
Iowa	USA	1.00	0.12	3.48
Kansas	USA	1.00	0.12	1.06
Kentucky	USA	1.00	0.12	0.12
Louisiana	USA	1.00	0.12	0.32
Maine	USA	1.00	0.12	0.10
Maryland	USA	1.00	0.12	0.09
Massachusetts	USA	1.00	0.12	0.17
Michigan	USA	1.00	0.12	0.06
Minnesota	USA	1.00	0.12	1.97
Mississippi	USA	1.00	0.12	0.08
Missouri	USA	1.00	0.12	0.17
Montana	USA	1.00	0.12	0.15
Nebraska	USA	1.00	0.25	6.52
Nevada	USA	1.00	0.12	0.05
New Hampshire	USA	1.00	0.12	0.06
New Jersey	USA	1.00	0.12	0.17
New Mexico	USA	1.00	0.12	0.27
New York	USA	1.00	0.12	0.13
North Carolina	USA	1.00	0.12	0.15
North Dakota	USA	1.00	0.12	13.46
Ohio	USA	1.00	0.12	0.09
Oklahoma	USA	1.00	0.12	0.20
Oregon	USA	1.00	0.12	0.04
Pennsylvania	USA	1.00	0.12	0.05
Puerto Rico	USA	1.00	0.12	0.22
Rhode Island	USA	1.00	0.12	0.16
South Carolina	USA	1.00	0.12	0.13
South Dakota	USA	1.00	0.25	1.73
Tennessee	USA	1.00	0.12	0.05
Texas	USA	1.00	0.12	0.06
Utah	USA	1.00	0.25	2.20
Vermont	USA	1.00	0.25	1.71
Virginia	USA	1.00	0.12	0.16
Washington	USA	1.00	0.12	0.12
West Virginia	USA	1.00	0.12	0.09
Wisconsin	USA	1.00	0.25	1.88
Wyoming	USA	1.00	0.12	0.04

Supplementary Table 11: Table of statistical tests comparing the census and synthetic population enrollment rates. Country codes are used to refer to the country name (see Table 1 for the country codes).

Employment Rates: Census vs. Synthetic Pop. Statistical Tests

Location	Country	Pearson's r	KS	RMSE
Australian Capital Territory	AUS	1.00	0.05	0.40
New South Wales	AUS	1.00	0.05	0.24
Northern Territory	AUS	1.00	0.06	2.27
Queensland	AUS	1.00	0.06	0.17
South Australia	AUS	1.00	0.06	0.29
Tasmania	AUS	1.00	0.05	0.27
Victoria	AUS	1.00	0.06	0.26
Western Australia	AUS	1.00	0.05	0.19
Alberta	CAN	1.00	0.14	0.00
British Columbia	CAN	1.00	0.14	0.00
Manitoba	CAN	1.00	0.14	0.00
New Brunswick	CAN	1.00	0.14	0.00
Newfoundland & Labrador	CAN	1.00	0.14	0.00
Nova Scotia	CAN	1.00	0.14	0.00
Ontario	CAN	1.00	0.14	0.00
Prince Edward Island	CAN	1.00	0.21	0.00
Quebec	CAN	1.00	0.14	0.00
Saskatchewan	CAN	1.00	0.14	0.00
Yukon	CAN	1.00	0.14	0.00
Anhui	CHN	1.00	0.08	0.04
Beijing	CHN	1.00	0.08	0.02
China	CHN	1.00	0.08	0.03
Chongqing	CHN	1.00	0.15	0.05
Fujian	CHN	1.00	0.08	0.04
Gansu	CHN	1.00	0.08	0.05
Guangdong	CHN	1.00	0.08	0.08
Guangxi	CHN	1.00	0.08	0.08
Guizhou	CHN	1.00	0.08	0.16
Hainan	CHN	1.00	0.08	0.05
Hebei	CHN	1.00	0.08	0.03
Heilongjiang	CHN	1.00	0.08	0.11
Henan	CHN	1.00	0.08	0.05
Hubei	CHN	1.00	0.08	0.06
Hunan	CHN	1.00	0.08	0.05
Inner Mongolia	CHN	1.00	0.08	0.09
Jiangsu	CHN	1.00	0.15	0.03
Jiangxi	CHN	1.00	0.08	0.09
Jilin	CHN	1.00	0.08	0.12
Liaoning	CHN	1.00	0.08	0.06
Ningxia	CHN	1.00	0.08	0.07
Qinghai	CHN	1.00	0.15	0.22
Shaanxi	CHN	1.00	0.08	0.05
Shandong	CHN	1.00	0.15	0.06
Shanghai	CHN	1.00	0.15	0.11
Shanxi	CHN	1.00	0.08	0.08
Sichuan	CHN	1.00	0.08	0.04
Tianjin	CHN	1.00	0.08	0.04
Tibet	CHN	1.00	0.31	0.66
Xinjiang	CHN	1.00	0.08	0.10
Yunnan	CHN	1.00	0.08	0.21
Zhejiang	CHN	1.00	0.08	0.04
Uttar Pradesh	IND	0.99	0.15	10.18
Rajasthan	IND	1.00	0.08	2.27
Haryana	IND	1.00	0.08	2.18
Puducherry	IND	1.00	0.08	0.53
West Bengal	IND	1.00	0.08	4.60
Andhra Pradesh	IND	1.00	0.08	3.29
Arunachal Pradesh	IND	0.99	0.08	19.99
Sikkim	IND	1.00	0.08	9.90
Assam	IND	0.99	0.08	11.26
Uttarakhand	IND	1.00	0.15	6.66
Tamil Nadu	IND	1.00	0.08	0.00
Mizoram	IND	1.00	0.08	3.95
Himachal Pradesh	IND	1.00	0.15	9.39
Meghalaya	IND	0.99	0.15	12.96
Gujarat	IND	1.00	0.08	3.49
Odisha	IND	1.00	0.08	2.14
Maharashtra	IND	1.00	0.08	5.42
Nct of Delhi	IND	1.00	0.08	1.32

Location	Country	Pearson's r	KS	RMSE
Chhattisgarh	IND	1.00	0.15	7.11
Manipur	IND	1.00	0.15	7.47
Bihar	IND	0.99	0.15	10.16
Tripura	IND	1.00	0.15	6.00
Jammu & Kashmir	IND	1.00	0.15	4.02
Punjab	IND	1.00	0.08	2.84
Goa	IND	1.00	0.15	1.08
Madhya Pradesh	IND	1.00	0.08	8.61
Jharkhand	IND	1.00	0.08	5.22
Andaman & Nicobar Islands	IND	1.00	0.08	4.05
Karnataka	IND	1.00	0.08	3.78
Daman & Diu	IND	1.00	0.08	6.98
Nagaland	IND	0.98	0.15	48.43
Kerala	IND	1.00	0.08	0.58
Israel	ISR	1.00	0.22	0.19
Aichi	JPN	0.99	0.06	21.18
Akita	JPN	1.00	0.12	4.25
Aomori	JPN	1.00	0.06	6.30
Chiba	JPN	1.00	0.12	10.06
Ehime	JPN	1.00	0.12	5.15
Fukui	JPN	1.00	0.12	9.46
Fukuoka	JPN	1.00	0.06	11.88
Fukushima	JPN	1.00	0.06	6.27
Gifu	JPN	1.00	0.12	4.77
Gumma	JPN	1.00	0.06	7.70
Hiroshima	JPN	0.99	0.12	13.09
Hokkaido	JPN	1.00	0.06	6.19
Hyogo	JPN	1.00	0.06	6.87
Ibaraki	JPN	1.00	0.12	6.83
Ishikawa	JPN	0.99	0.06	19.11
Iwate	JPN	1.00	0.12	8.04
Kagawa	JPN	1.00	0.12	5.98
Kagoshima	JPN	1.00	0.06	7.69
Kanagawa	JPN	1.00	0.12	5.97
Kochi	JPN	1.00	0.06	8.37
Kumamoto	JPN	1.00	0.12	10.40
Kyoto	JPN	0.94	0.18	164.87
Mie	JPN	1.00	0.12	7.06
Miyagi	JPN	1.00	0.06	6.04
Miyazaki	JPN	1.00	0.06	6.75
Nagano	JPN	1.00	0.06	8.41
Nagasaki	JPN	1.00	0.06	5.47
Nara	JPN	1.00	0.12	3.40
Niigata	JPN	1.00	0.12	6.07
Oita	JPN	1.00	0.12	7.81
Okayama	JPN	1.00	0.06	9.54
Okinawa	JPN	1.00	0.12	7.18
Osaka	JPN	0.99	0.12	19.03
Saga	JPN	1.00	0.12	7.34
Saitama	JPN	1.00	0.06	7.09
Shiga	JPN	1.00	0.12	5.58
Shimane	JPN	1.00	0.12	11.30
Shizuoka	JPN	1.00	0.12	10.05
Tochigi	JPN	1.00	0.12	6.48
Tokushima	JPN	1.00	0.12	5.63
Tokyo	JPN	0.94	0.18	186.61
Tottori	JPN	1.00	0.12	6.85
Toyama	JPN	1.00	0.12	3.41
Wakayama	JPN	1.00	0.06	2.16
Yamagata	JPN	1.00	0.06	8.27
Yamaguchi	JPN	1.00	0.06	2.68
Yamanashi	JPN	1.00	0.06	3.23
Adygea	RUS	1.00	0.02	0.14
Altai Krai	RUS	1.00	0.03	0.16
Altai Republic	RUS	1.00	0.03	0.21
Amur Oblast	RUS	1.00	0.02	0.10
Arkhangelsk Oblast	RUS	1.00	0.03	0.14
Astrakhan Oblast	RUS	1.00	0.03	0.13
Bashkortostan	RUS	1.00	0.04	0.14

Supplementary Table 12: Table of statistical tests comparing the census and synthetic population employment rates. Country codes are used to refer to the country name (see Table 1 for the country codes).

Employment Rates: Census vs. Synthetic Pop. Statistical Tests

Location	Country	Pearson's r	KS	RMSE
Belgorod Oblast	RUS	1.00	0.03	0.14
Bryansk Oblast	RUS	1.00	0.03	0.14
Buryatia	RUS	1.00	0.05	0.15
Chechnya	RUS	1.00	0.03	0.10
Chelyabinsk Oblast	RUS	1.00	0.03	0.12
Chukotka	RUS	1.00	0.02	0.28
Chuvashia	RUS	1.00	0.04	0.14
Dagestan	RUS	1.00	0.05	0.14
Ingushetia	RUS	1.00	0.03	0.09
Irkutsk Oblast	RUS	1.00	0.02	0.12
Ivanovo Oblast	RUS	1.00	0.05	0.15
Jewish Auto. Oblast	RUS	1.00	0.02	0.18
Kabardino Balkaria	RUS	1.00	0.03	0.12
Kaliningrad Oblast	RUS	1.00	0.03	0.14
Kalmykia	RUS	1.00	0.04	0.12
Kaluga Oblast	RUS	1.00	0.03	0.21
Kamchatka Krai	RUS	1.00	0.03	0.14
Karachay Cherkessia	RUS	1.00	0.03	0.13
Karelia	RUS	1.00	0.03	0.17
Kemerovo Oblast	RUS	1.00	0.02	0.14
Khabarovsk Krai	RUS	1.00	0.04	0.21
Khakassia	RUS	1.00	0.04	0.10
Khanty Mansi Auto. Okrug	RUS	1.00	0.03	0.19
Kirov Oblast	RUS	1.00	0.02	0.13
Komi Republic	RUS	1.00	0.03	0.18
Kostroma Oblast	RUS	1.00	0.03	0.11
Krasnodar Krai	RUS	1.00	0.02	0.07
Krasnoyarsk Krai	RUS	1.00	0.03	0.22
Kurgan Oblast	RUS	1.00	0.04	0.13
Kursk Oblast	RUS	1.00	0.02	0.12
Leningrad Oblast	RUS	1.00	0.03	0.11
Lipetsk Oblast	RUS	1.00	0.03	0.16
Magadan Oblast	RUS	1.00	0.03	0.15
Mari El	RUS	1.00	0.03	0.15
Mordovia	RUS	1.00	0.03	0.13
Moscow Oblast	RUS	1.00	0.02	0.15
Moscow	RUS	1.00	0.02	0.12
Murmansk Oblast	RUS	1.00	0.04	0.13
Nenets Auto. Okrug	RUS	1.00	0.02	0.13
Nizhny Novgorod Oblast	RUS	1.00	0.04	0.19
North Ossetia Alania	RUS	1.00	0.03	0.17
Novgorod Oblast	RUS	1.00	0.03	0.16
Novosibirsk Oblast	RUS	1.00	0.02	0.13
Omsk Oblast	RUS	1.00	0.03	0.11
Orenburg Oblast	RUS	1.00	0.02	0.10
Oryol Oblast	RUS	1.00	0.02	0.10
Penza Oblast	RUS	1.00	0.03	0.12
Perm Krai	RUS	1.00	0.04	0.10
Primorsky Krai	RUS	1.00	0.04	0.21
Pskov Oblast	RUS	1.00	0.04	0.17
Rostov Oblast	RUS	1.00	0.03	0.12
Russian Federation	RUS	1.00	0.03	0.10
Ryazan Oblast	RUS	1.00	0.03	0.17
Sakha	RUS	1.00	0.04	0.16
Sakhalin Oblast	RUS	1.00	0.02	0.22
Samara Oblast	RUS	1.00	0.03	0.16
Saratov Oblast	RUS	1.00	0.02	0.12
Smolensk Oblast	RUS	1.00	0.02	0.15
St. Petersburg	RUS	1.00	0.03	0.20
Stavropol Krai	RUS	1.00	0.03	0.13
Sverdlovsk Oblast	RUS	1.00	0.02	0.16
Tambov Oblast	RUS	1.00	0.03	0.08
Tatarstan	RUS	1.00	0.04	0.11
Tomsk Oblast	RUS	1.00	0.02	0.18
Tula Oblast	RUS	1.00	0.02	0.12
Tuva	RUS	1.00	0.04	0.13
Tver Oblast	RUS	1.00	0.04	0.17
Tyumen Oblast	RUS	1.00	0.03	0.19
Udmurtia	RUS	1.00	0.03	0.11
Ulyanovsk Oblast	RUS	1.00	0.03	0.17
Vladimir Oblast	RUS	1.00	0.03	0.14
Volgograd Oblast	RUS	1.00	0.02	0.11
Vologda Oblast	RUS	1.00	0.03	0.10
Voronezh Oblast	RUS	1.00	0.04	0.14
Yamalo Nenets Auto. Okrug	RUS	1.00	0.02	0.13
Yaroslavl Oblast	RUS	1.00	0.05	0.15
Zabaykalsky Krai	RUS	1.00	0.03	0.15
Eastern Cape	ZAF	1.00	0.07	0.00
Free State	ZAF	1.00	0.07	0.00
Gauteng	ZAF	1.00	0.07	0.00
KwaZulu Natal	ZAF	1.00	0.07	0.00
Limpopo	ZAF	1.00	0.07	0.00
Mpumalanga	ZAF	1.00	0.07	0.00
Northern Cape	ZAF	1.00	0.07	0.00
North West	ZAF	1.00	0.07	0.00
Western Cape	ZAF	1.00	0.07	0.00
Alabama	USA	1.00	0.14	1.43
Alaska	USA	1.00	0.14	5.23
Arizona	USA	1.00	0.14	0.85
Arkansas	USA	1.00	0.14	1.65
California	USA	1.00	0.14	2.25
Colorado	USA	1.00	0.14	4.56
Connecticut	USA	1.00	0.14	4.09
Delaware	USA	1.00	0.14	2.20
District of Columbia	USA	1.00	0.29	5.16
Florida	USA	1.00	0.14	1.41
Georgia	USA	1.00	0.29	2.20
Hawaii	USA	1.00	0.14	5.23
Idaho	USA	1.00	0.14	1.59
Illinois	USA	1.00	0.29	2.21
Indiana	USA	1.00	0.14	1.62
Iowa	USA	1.00	0.14	6.94
Kansas	USA	1.00	0.14	3.53
Kentucky	USA	1.00	0.14	1.25
Louisiana	USA	1.00	0.14	2.18
Maine	USA	1.00	0.14	2.24
Maryland	USA	1.00	0.14	4.98
Massachusetts	USA	1.00	0.14	4.08
Michigan	USA	1.00	0.14	1.18
Minnesota	USA	1.00	0.14	4.46
Mississippi	USA	1.00	0.14	1.75
Missouri	USA	1.00	0.14	2.09
Montana	USA	1.00	0.14	3.43
Nebraska	USA	0.99	0.14	12.52
Nevada	USA	1.00	0.14	2.16
New Hampshire	USA	1.00	0.14	3.04
New Jersey	USA	1.00	0.14	4.56
New Mexico	USA	1.00	0.29	1.73
New York	USA	1.00	0.14	2.06
North Carolina	USA	1.00	0.14	1.46
North Dakota	USA	0.99	0.14	16.54
Ohio	USA	1.00	0.14	2.16
Oklahoma	USA	1.00	0.14	2.25
Oregon	USA	1.00	0.14	1.45
Pennsylvania	USA	1.00	0.14	2.63
Puerto Rico	USA	1.00	0.14	0.37
Rhode Island	USA	1.00	0.14	2.88
South Carolina	USA	1.00	0.14	1.18
South Dakota	USA	1.00	0.14	5.98
Tennessee	USA	1.00	0.14	1.67
Texas	USA	1.00	0.29	3.30
Utah	USA	1.00	0.14	2.95
Vermont	USA	0.99	0.14	10.32
Virginia	USA	1.00	0.14	3.29
Washington	USA	1.00	0.29	1.89
West Virginia	USA	1.00	0.14	0.57
Wisconsin	USA	1.00	0.14	3.52
Wyoming	USA	1.00	0.14	3.48

Supplementary Table 13: Table of statistical tests comparing the census and synthetic population employment rates. Country codes are used to refer to the country name (see Table 1 for the country codes).

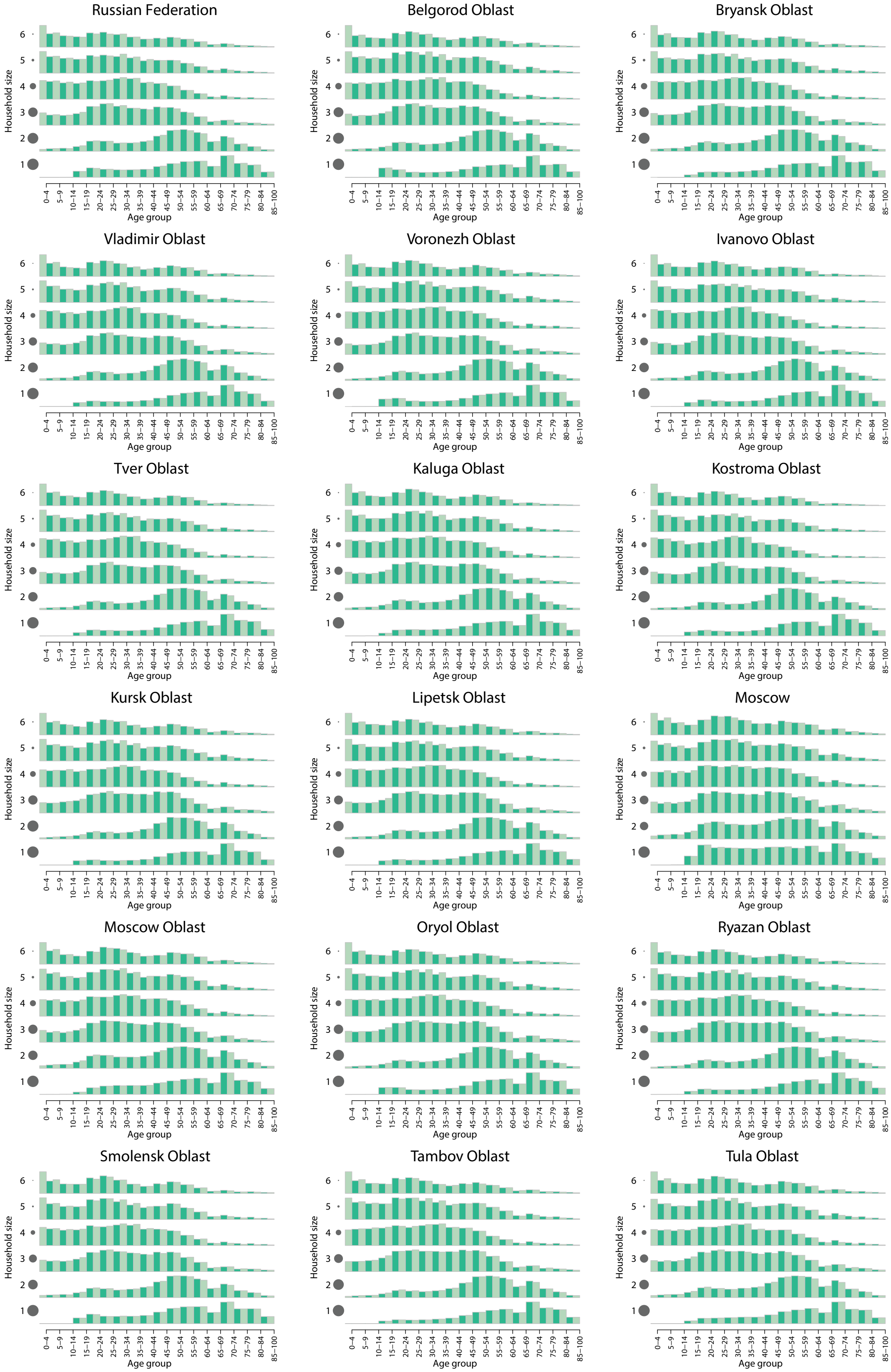

Supplementary Figure 50: **Age structure by household size in Russia and its region.** Dark bars refer to the data and light bars to the synthetic population. The area of the grey circles is proportional to the share of household of a given size according to the data.

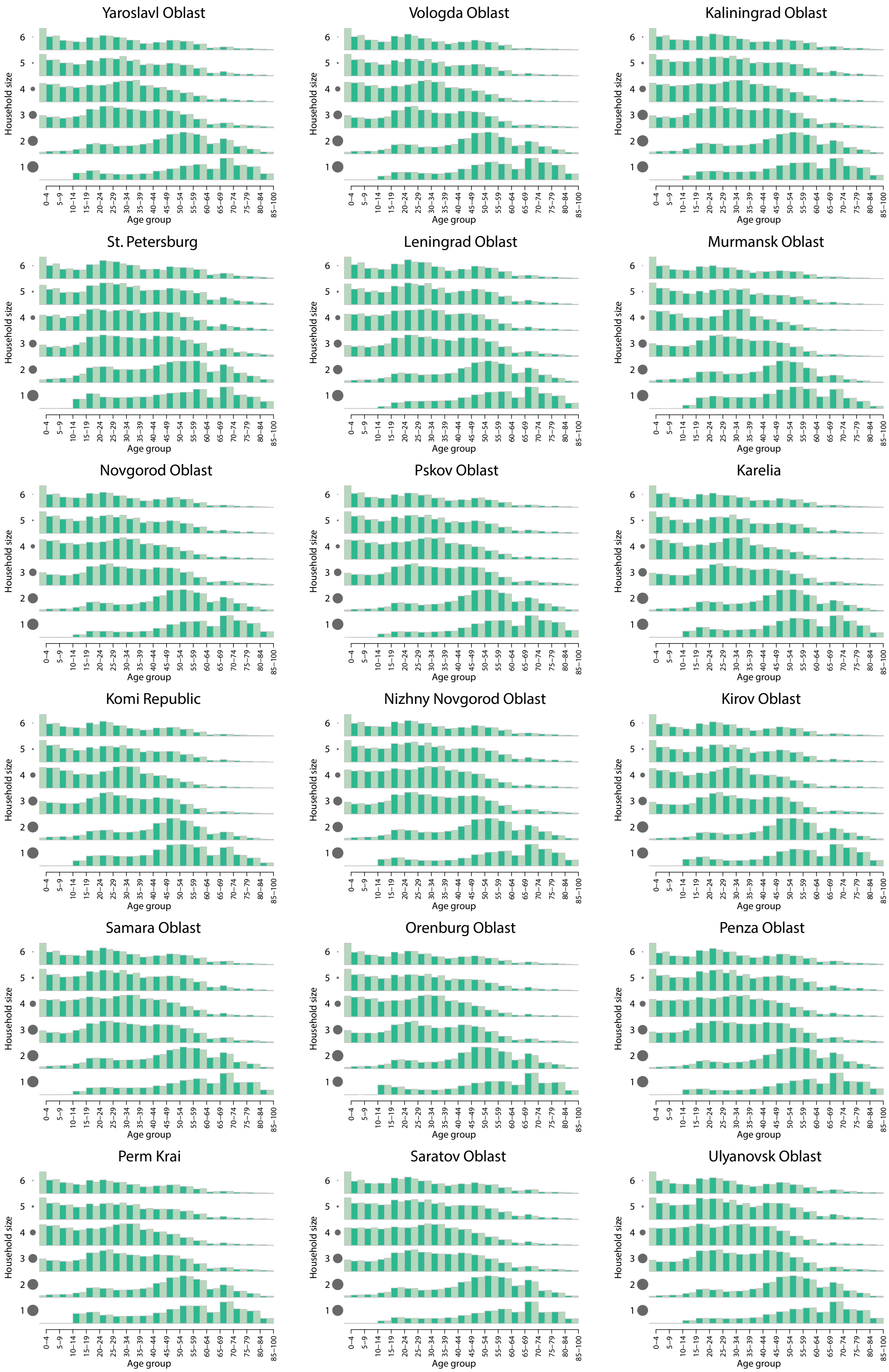

Supplementary Figure 51: **Age structure by household size in Russia and its region.** Continuation.

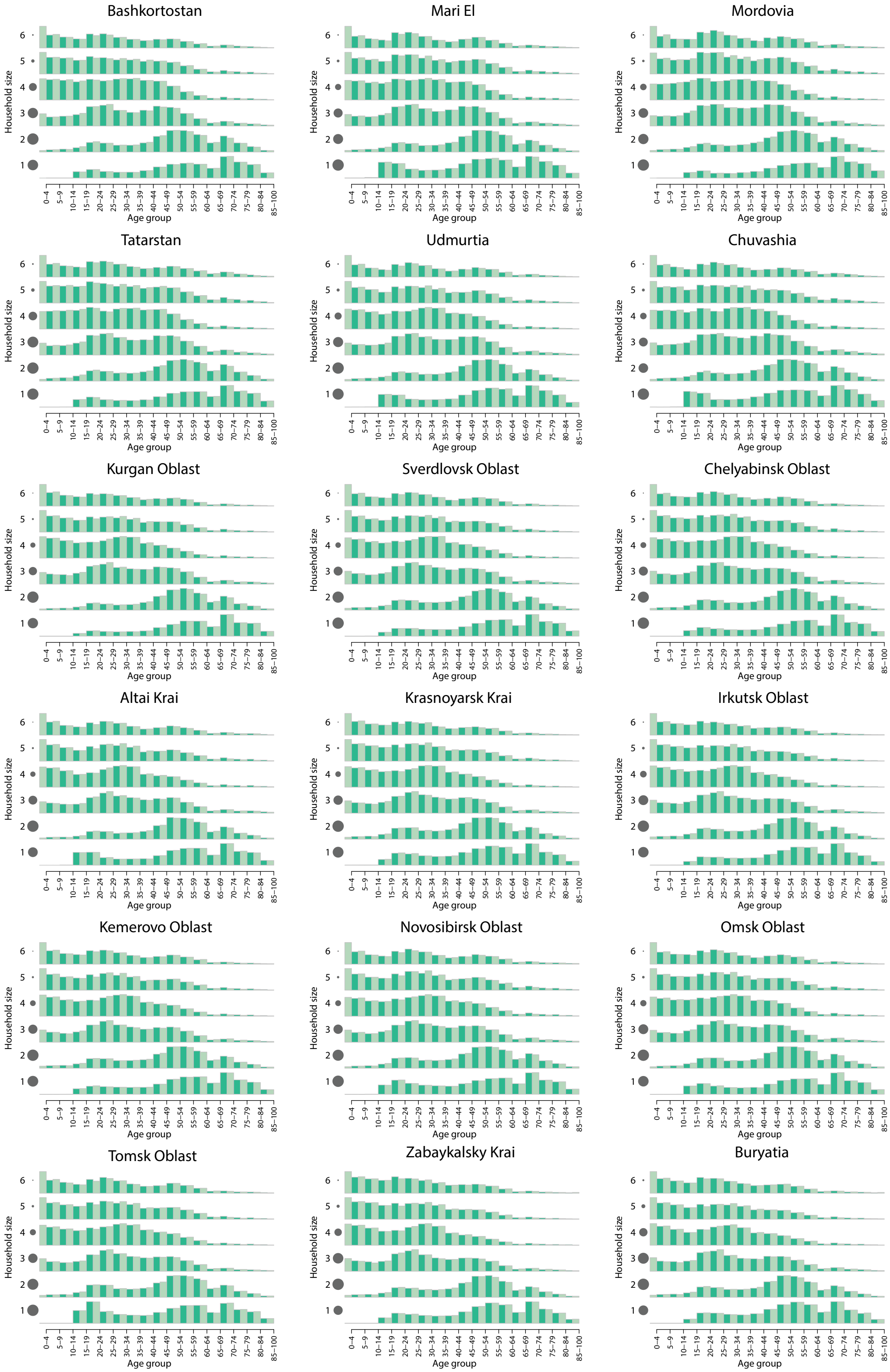

Supplementary Figure 52: **Age structure by household size in Russia and its region.** Continuation.

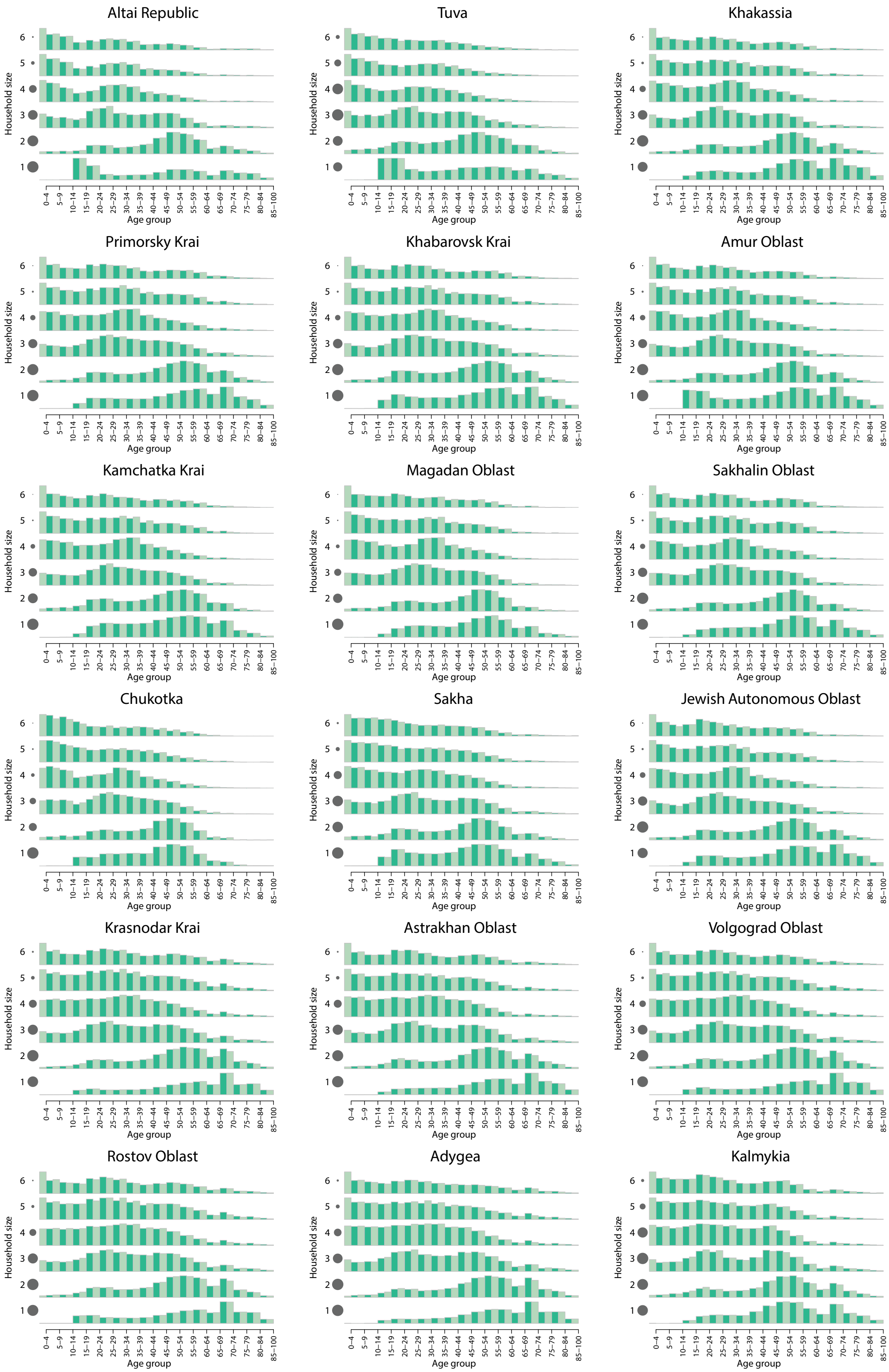

Supplementary Figure 53: **Age structure by household size in Russia and its region.** Continuation.

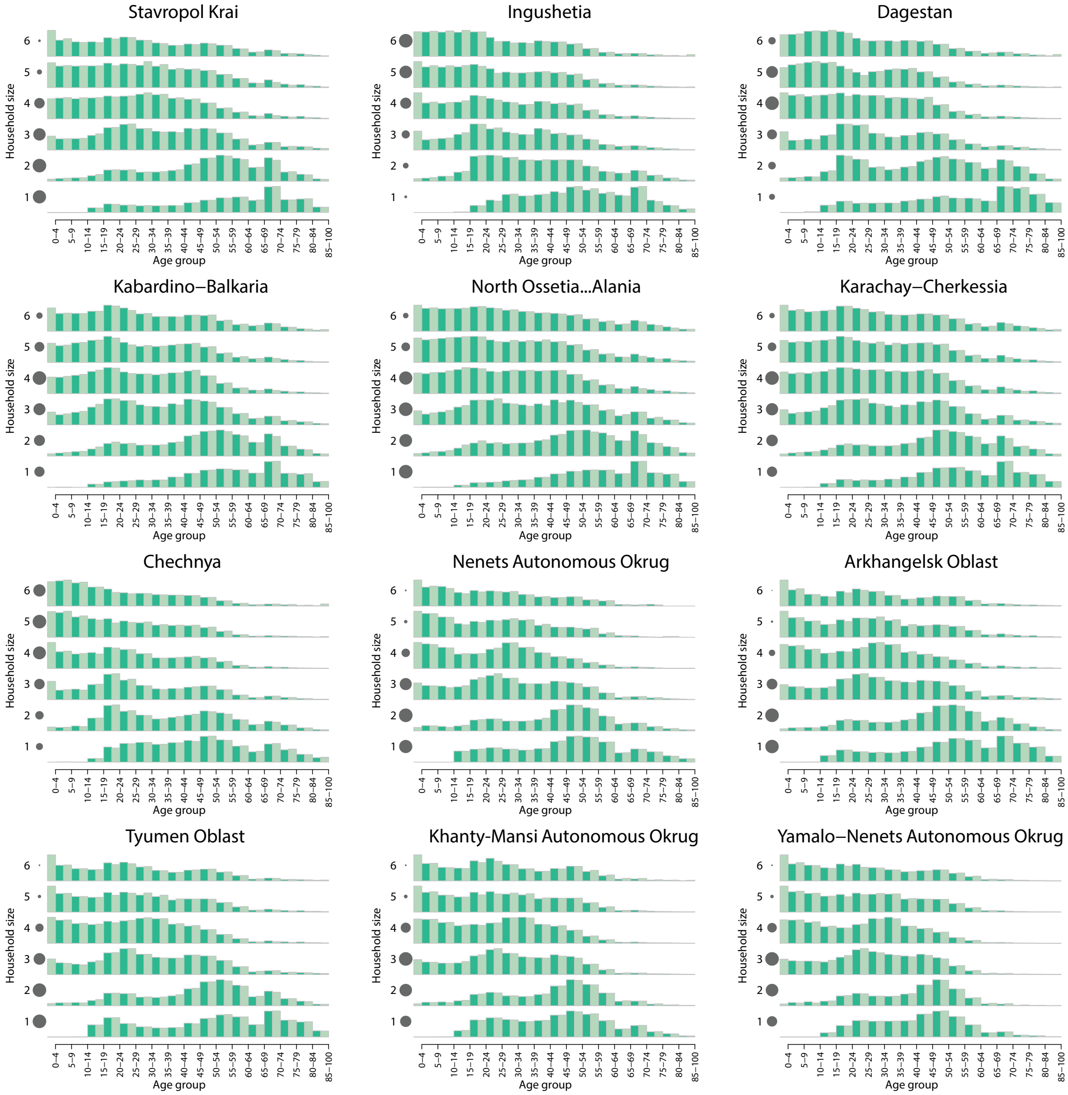

Supplementary Figure 54: **Age structure by household size in Russia and its region.** Continuation.

References

[1] Australian Bureau of Statistics (2011) Australian Bureau of Statistics (http://www.abs.gov.au/). Online; accessed May 27, 2016.

[2] Statistics Canada (2011) Statistics Canada (https://www.statcan.gc.ca/eng/start). Online; accessed Oct. 15, 2015.

[3] Government of British Columbia (2011) BC Stats (https://www2.gov.bc.ca/). Online; accessed Sept. 8, 2016.

[4] Childcare Resource and Research Unit, Canadian Union of Postal Workers (2014) Finding Quality Child Care: A guide for parents in Canada (https://findingqualitychildcare.ca/). Online; accessed Sept. 10, 2016.

[5] National Institute for Nutrition and Health, China Center for Disease Control and Prevention, Carolina Population Center, University of North Carolina at Chapel Hill (2009) China Health and Nutrition Survey (CHNS) (https://www.cpc.unc.edu/projects/china/data/datasets). Online; accessed Feb. 27, 2017.

[6] China Statistics Press (2010) Census 2010 (http://www.stats.gov.cn/tjsj/pcsj/rkpc/6rp/indexch.htm). Online; accessed Feb. 22, 2017.

[7] China Statistics Press (2010) China Statistical Yearbook 2010 (http://www.stats.gov.cn/tjsj/ndsj/2010/indexeh.htm). Online; accessed Feb. 22, 2017.

[8] Office of the Registrar General and Census Commissioner, Ministry of Home Affairs, Government of India (2011) 2011 Census Data (http://www.censusindia.gov.in/pca/Searchdata.aspx). Online; accessed Oct. 14, 2015.

[9] Demographic and Health Surveys (2005-2006) India: Standard DHS 2005-06 (https://dhsprogram.com/data/dataset/India_Standard-DHS_2006.cfm?flag=0). Online; accessed July 18, 2016.

[10] Unified District Information System for Education (UDISE), National Institute of Educational Planning and Adminstration (2011-2012) Elementary Education in India (http://udise.in/src.htm). Online; accessed June 26, 2016.

[11] Unified District Information System for Education (UDISE), National Institute of Educational Planning and Adminstration (2012-2013) Secondary Education in India: State Report Cards 2012-13 (http://udise.in/src.htm). Online; accessed June 26, 2016.

[12] Department of Higher Education, Ministry of Human Resource Development, Government of India (2013) All India Survey on Higher Education (http://mhrd.gov.in/sites/upload_files/mhrd/files/statistics/AISHE2011-12P_1.pdf). Online; accessed June 26, 2016.

[13] Israel Central Bureau of Statistics (2008) Israel Census 2008 (http://www.cbs.gov.il/census/census/pnimi_page_e.html?id_topic=2). Online; accessed June 3, 2016.

[14] Japanese Government Statistics (2010) e-Stat, Portal Site of Official Statistics of Japan (https://www.e-stat.go.jp/en/). Online; accessed July 18, 2016.

[15] Popkin, Barry M. and Peter, Klara and Zohoori, Namvar and Entwisle, Barbara and Mroz, Tom and Kohlmeier, Lenore and Bardsley, Phil and Bender, Ekaterina and Blanchette, Dan and Bontch-Osmolovskii, Mikhail and Cadwell, Suzanne and Cross, Catherine and Gallagher,

Kelly and Gleiter, Karin and Glinskaya, Elena and Henderson, Laura and Kier, Lauren and Kitsul, Tamara and Kline, Laura and Kowalsky, Sharon and Lokshin, Michael and Lukashov, Andrey and Mancini, Dominic and Miles, Donna and O'Hara, Rick and Murphy, Jennifer S. and Robinson, David and Watterson, Loren and Young, Anthony (2010) The Russia Longitudinal Monitoring Survey (RLMS) (https://www.cpc.unc.edu/projects/china/data/datasets). Online; accessed Feb. 27, 2017.

[16] Federal State Statistics Service (2010) 2010 All-Russian Population Census (http://www.gks.ru/free_doc/new_site/perepis2010/croc/perepis_itogi1612.htm). Online; accessed Feb. 21, 2017.

[17] Federal State Statistics Service (2010) Labor market, employment and wages (http://www.gks.ru/wps/wcm/connect/rosstat_main/rosstat/ru/statistics/wages/labour_force/#). Online; accessed Feb. 21, 2017.

[18] Statistics South Africa (2011) Census 2011 (http://www.statssa.gov.za/). Online; accessed June 6, 2016.

[19] Department: Higher Education and Training, Republic of South Africa (2008) Statistics on Post-School Education and Training in South Africa: 2011 (http://www.cbs.gov.il/census/census/pnimi_page_e.html?id_topic=2). Online; accessed June 3, 2016.

[20] World Health Organization (WHO) (2003-2005) World Health Survey (http://apps.who.int/healthinfo/systems/surveydata/index.php/catalog). Online; accessed June 3, 2017.

[21] South African Revenue Service, National Treasury (2013) 2013 Tax Statistics (http://www.sars.gov.za/About/SATaxSystem/Pages/Tax-Statistics.aspx). Online; accessed June 7, 2017.

[22] United States Census Bureau (2010) Decennial Census of Population and Housing (https://www.census.gov/programs-surveys/decennial-census/decade.2010.html). Online; accessed Apr. 3, 2017.

[23] United States Census Bureau (2010) Current Population Survey (https://www.census.gov/programs-surveys/cps/data-detail.html). Online; accessed Apr. 3, 2017.

[24] United States Census Bureau (2010) American Community Survey (https://www.census.gov/programs-surveys/acs/data.html). Online; accessed Apr. 3, 2017.

[25] Ruggles, Steven and Flood, Sarah and Goeken, Ronald and Grover, Josiah and Meyer, Erin and Pacas, Jose and Sobek, Matthew (2010) IPUMS USA: Version 8.0 [dataset] (https://doi.org/10.18128/D010.V8.0). Online; accessed Oct. 30, 2016.

[26] Mossong J et al. (2008) Social contacts and mixing patterns relevant to the spread of infectious diseases. *PLOS Med* 5(3):e74.

[27] Ajelli M, Litvinova M (2017) Estimating contact patterns relevant to the spread of infectious diseases in Russia. *J Theor Biol* 419:1–7.

[28] Greenhalgh D, Dietz K (1994) Some bounds on estimates for reproductive ratios derived from the age-specific force of infection. *Math Biosci* 124(1):9–57.

[29] Fumanelli L, Ajelli M, Manfredi P, Vespignani A, Merler S (2012) Inferring the structure of social contacts from demographic data in the analysis of infectious diseases spread. *PLOS Comput Biol* 8(9):e1002673.

[30] Zhang J et al. (2020) Changes in contact patterns shape the dynamics of the COVID-19 outbreak in China. *Science* 368(6498):1481–1486.

[31] Fraser C et al. (2009) Pandemic potential of a strain of influenza A (H1N1): early findings. *Science* 324(5934):1557–1561.

[32] Cauchemez S et al. (2009) Household transmission of 2009 pandemic influenza A (H1N1) virus in the United States. *N Engl J Med* 2009(361):2619–2627.

[33] Ajelli M, Poletti P, Melegaro A, Merler S (2014) The role of different social contexts in shaping influenza transmission during the 2009 pandemic. *Sci Rep* 4:7218.

[34] Anderson RM, May RM, Anderson B (1991) *Infectious diseases of humans: dynamics and control.* (Oxford University Press; Oxford, UK).

[35] Diekmann O, Heesterbeek JAP, Metz JA (1990) On the definition and the computation of the basic reproduction ratio R0 in models for infectious diseases in heterogeneous populations. *J Math Biol* 28(4):365–382.

[36] Weil M et al. (2013) The dynamics of infection and the persistence of immunity to A (H1N1) pdm09 virus in Israel. *Influenza Other Respir Viruses* 7(5):838–846.

[37] Merler S et al. (2013) Pandemic influenza A/H1N1pdm in Italy: age, risk and population susceptibility. *PLOS One* 8(10):e74785.

[38] Japanese Infectious Disease Surveillance Center (2009) Survey on the possession of influenza antibodies in FY 2009 - First Report. Online; accessed May 2, 2017.

[39] Japanese Infectious Disease Surveillance Center (2010) Influenza antibody holding status survey in FY 2010 - First Report. Online; accessed June 14, 2018.

[40] Hardelid P et al. (2010) Assessment of baseline age-specific antibody prevalence and incidence of infection to novel influenza A/H1N1 2009. *Health Technol Assess* 14(55):115–92.

[41] Reed C, Katz JM, Hancock K, Balish A, Fry AM (2012) Prevalence of seropositivity to pandemic influenza A/H1N1 virus in the United States following the 2009 pandemic. *PLOS One* 7(10):e48187.

[42] Cauchemez S, Carrat F, Viboud C, Valleron A, Boelle P (2004) A Bayesian MCMC approach to study transmission of influenza: application to household longitudinal data. *Stat Med* 23(22):3469–3487.

[43] Merler S, Ajelli M, Pugliese A, Ferguson NM (2011) Determinants of the spatiotemporal dynamics of the 2009 H1N1 pandemic in Europe: implications for real-time modelling. *PLOS Comput Biol* 7(9):e1002205.

[44] Dorigatti I, Cauchemez S, Ferguson NM (2013) Increased transmissibility explains the third wave of infection by the 2009 H1N1 pandemic virus in England. *Proc Natl Acad Sci USA* 110(33):13422–13427.

[45] Marziano V, Pugliese A, Merler S, Ajelli M (2017) Detecting a Surprisingly Low Transmission Distance in the Early Phase of the 2009 Influenza Pandemic. *Sci Rep* 7(1):12324.

[46] Biggerstaff M, Cauchemez S, Reed C, Gambhir M, Finelli L (2014) Estimates of the reproduction number for seasonal, pandemic, and zoonotic influenza: a systematic review of the literature. *BMC Infect Dis* 14(1):480.

[47] Wallinga J, Teunis P, Kretzschmar M (2006) Using data on social contacts to estimate age-specific transmission parameters for respiratory-spread infectious agents. *Am J Epidemiol* 164(10):936–944.
