# Supplementary material for: Inferring high-resolution human mixing patterns for disease modeling
Source: Nat Commun. 2021 Jan 12;12:323. doi: 10.1038/s41467-020-20544-y (PMC7803761; doi:10.1038/s41467-020-20544-y)
Supplement: Supplementary file 2 — Reporting Summary [file 41467_2020_20544_MOESM2_ESM.pdf]

nature research

Corresponding author(s): Marco Ajelli, Alessandro Vespignani

Last updated by author(s): Nov 23, 2020

# Reporting Summary

Nature Research wishes to improve the reproducibility of the work that we publish. This form provides structure for consistency and transparency in reporting. For further information on Nature Research policies, see our Editorial Policies and the Editorial Policy Checklist.

## Statistics

For all statistical analyses, confirm that the following items are present in the figure legend, table legend, main text, or Methods section.

| n/a | Confirmed | |
|---|---|---|
| ☐ | ☒ | The exact sample size (*n*) for each experimental group/condition, given as a discrete number and unit of measurement |
| ☐ | ☒ | A statement on whether measurements were taken from distinct samples or whether the same sample was measured repeatedly |
| ☐ | ☒ | The statistical test(s) used AND whether they are one- or two-sided<br>*Only common tests should be described solely by name; describe more complex techniques in the Methods section.* |
| ☒ | ☐ | A description of all covariates tested |
| ☐ | ☒ | A description of any assumptions or corrections, such as tests of normality and adjustment for multiple comparisons |
| ☐ | ☒ | A full description of the statistical parameters including central tendency (e.g. means) or other basic estimates (e.g. regression coefficient) AND variation (e.g. standard deviation) or associated estimates of uncertainty (e.g. confidence intervals) |
| ☐ | ☒ | For null hypothesis testing, the test statistic (e.g. *F*, *t*, *r*) with confidence intervals, effect sizes, degrees of freedom and *P* value noted<br>*Give P values as exact values whenever suitable.* |
| ☐ | ☒ | For Bayesian analysis, information on the choice of priors and Markov chain Monte Carlo settings |
| ☒ | ☐ | For hierarchical and complex designs, identification of the appropriate level for tests and full reporting of outcomes |
| ☐ | ☒ | Estimates of effect sizes (e.g. Cohen's *d*, Pearson's *r*), indicating how they were calculated |

*Our web collection on statistics for biologists contains articles on many of the points above.*

## Software and code

Policy information about availability of computer code

| | |
|---|---|
| Data collection | The data sources are provided as references in the manuscript and supplementary material. No specific software was used for the data collection. |
| Data analysis | Python V3.8.3 and R V3.6.2 |

For manuscripts utilizing custom algorithms or software that are central to the research but not yet described in published literature, software must be made available to editors and reviewers. We strongly encourage code deposition in a community repository (e.g. GitHub). See the Nature Research guidelines for submitting code & software for further information.

## Data

Policy information about availability of data

All manuscripts must include a data availability statement. This statement should provide the following information, where applicable:

- Accession codes, unique identifiers, or web links for publicly available datasets
- A list of figures that have associated raw data
- A description of any restrictions on data availability

A database containing the inferred setting-specific matrices as well as the contact matrices for influenza transmission for all locations (and countries) is publicly available on the dedicated online repository: https://github.com/mobs-lab/mixing-patterns. DOI:10.5281/zenodo.4287574

Data Sources:
Australia: Australian Bureau of Statistics; Canada: Statistics Canada, BC Stats, Finding Quality Childcare: A guide for parents in Canada; China: China Health and Nutrition Survey (CHNS), China Census 2010, China Statistical Yearbook; India: The 15th Indian Census Demographic and Health Surveys (2005), Unified District Information System for Education, All India Survey on Higher Education; Israel: Israel Census 2008; Japan: JPN Official Statistics of Japan; Russia: Russia Longitudinal

Monitoring Survey (RLMS-HSE), All-Russian Population Census (2010), Federal State Statistic Service; South Africa: Statistics South Africa, Statistics on Post-School Education and Training in South Africa, World Health Survey (2003), South African Revenue Service; United States: Decennial Census of Population and Housing , Current Population Survey, American Community Survey , IPUMS USA

# Field-specific reporting

Please select the one below that is the best fit for your research. If you are not sure, read the appropriate sections before making your selection.

☐ Life sciences ☒ Behavioural & social sciences ☐ Ecological, evolutionary & environmental sciences

For a reference copy of the document with all sections, see nature.com/documents/nr-reporting-summary-flat.pdf

# Behavioural & social sciences study design

All studies must disclose on these points even when the disclosure is negative.

| | |
|---|---|
| Study description | We quantify and model human contact patterns from aggregated survey data. We are not generating primary data and the data that we used in the modeling were not collected in this study. |
| Research sample | We use publicly available data combining census and survey data. The different sources included: Australia: Australian Bureau of Statistics; Canada: Statistics Canada, BC Stats, Finding Quality Childcare: A guide for parents in Canada; China: China Health and Nutrition Survey (CHNS), China Census 2010, China Statistical Yearbook; India: The 15th Indian Census Demographic and Health Surveys (2005), Unified District Information System for Education, All India Survey on Higher Education; Israel: Israel Census 2008; Japan: JPN Official Statistics of Japan; Russia: Russia Longitudinal Monitoring Survey (RLMS-HSE), All-Russian Population Census (2010), Federal State Statistics Service; South Africa: Statistics South Africa, Statistics on Post-School Education and Training in South Africa, World Health Survey (2003), South African Revenue Service; United States: Decennial Census of Population and Housing, Current Population Survey, American Community Survey, IPUMS USA |
| Sampling strategy | NA. We use all the available data in the databases. Micro data was re-sampled proportional to strata defined by the characteristics from census/official statistics as described in the manuscript. |
| Data collection | We referred to official census databases, as listed above. The data was in a variety of formats, always digital, from csv, stata, sas files to pdf files. |
| Timing | NA. Timing of the data collection is not applicable. The data for each individual country was gathered for relatively similar reference periods. |
| Data exclusions | No data were excluded. |
| Non-participation | NA. We work directly with surveys and census data provided through the websites and publications listed in the manuscript. We did not survey the individuals. |
| Randomization | NA. We did not consider individuals participants. |

# Reporting for specific materials, systems and methods

We require information from authors about some types of materials, experimental systems and methods used in many studies. Here, indicate whether each material, system or method listed is relevant to your study. If you are not sure if a list item applies to your research, read the appropriate section before selecting a response.

## Materials & experimental systems

| n/a | Involved in the study | |
|---|---|---|
| ☒ | ☐ | Antibodies |
| ☒ | ☐ | Eukaryotic cell lines |
| ☒ | ☐ | Palaeontology and archaeology |
| ☒ | ☐ | Animals and other organisms |
| ☒ | ☐ | Human research participants |
| ☒ | ☐ | Clinical data |
| ☒ | ☐ | Dual use research of concern |

## Methods

| n/a | Involved in the study | |
|---|---|---|
| ☒ | ☐ | ChIP-seq |
| ☒ | ☐ | Flow cytometry |
| ☒ | ☐ | MRI-based neuroimaging |
